# Linking oxidative and reductive clusters to prepare crystalline porous catalysts for photocatalytic CO_2_ reduction with H_2_O

**DOI:** 10.1038/s41467-022-32449-z

**Published:** 2022-08-10

**Authors:** Jie Zhou, Jie Li, Liang Kan, Lei Zhang, Qing Huang, Yong Yan, Yifa Chen, Jiang Liu, Shun-Li Li, Ya-Qian Lan

**Affiliations:** School of Chemistry, South China Normal University, Guangzhou, 510006 P.R. China

**Keywords:** Photocatalysis, Coordination chemistry, Porous materials

## Abstract

Mimicking natural photosynthesis to convert CO_2_ with H_2_O into value-added fuels achieving overall reaction is a promising way to reduce the atmospheric CO_2_ level. Casting the catalyst of two or more catalytic sites with rapid electron transfer and interaction may be an effective strategy for coupling photocatalytic CO_2_ reduction and H_2_O oxidation. Herein, based on the MOF **∪** COF collaboration, we have carefully designed and synthesized a crystalline hetero-metallic cluster catalyst denoted MCOF-Ti_6_Cu_3_ with spatial separation and functional cooperation between oxidative and reductive clusters. It utilizes dynamic covalent bonds between clusters to promote photo-induced charge separation and transfer efficiency, to drive both the photocatalytic oxidative and reductive reactions. MCOF-Ti_6_Cu_3_ exhibits fine activity in the conversion of CO_2_ with water into HCOOH (169.8 μmol g^−1^h^−1^). Remarkably, experiments and theoretical calculations reveal that photo-excited electrons are transferred from Ti to Cu, indicating that the Cu cluster is the catalytic reduction center.

## Abstract (web summary)

A crystalline hetero-metallic cluster catalyst based on a covalent organic framework strategy is reported. The catalyst can facilitate both photocatalytic oxidative and reductive reactions leading to efficient production of HCOOH from CO2 and H2O.

## Introduction

Converting CO_2_ and H_2_O with solar energy into carbon-based fuels is a potential solution that could solve the problems of global warming and energy supply, which is in line with the global green and low-carbon development strategy^1–4^. However, there is still a long way to realize photocatalytic overall oxidative and reductive reactions, due to the inability of fast photogenerated electron-hole pairs recombination to satisfy both the kinetics of overcoming the high chemical inertness of CO_2_ molecules and the slow oxidation of water^5–9^. In nature, the chlorophyll aggregate P680 of green plants captures light energy for charge separation, then pheophytin pigment rapidly captures electrons to produce the radical cation P680^+^, providing separated electron-hole to complete the conversion of CO_2_ and H_2_O into carbon-based fuels^10–13^. Up to date, tremendous efforts have been made to mimic green plants, including type II and Z-scheme heterojunctions for the artificial photosynthesis systems^14–17^. Therefore, combining two or more catalytic sites to accomplish spatial separation of oxidative and reductive centers, rapid transfer of electrons, and functional interactions may be a promising strategy for realizing the overall photoreaction^18–20^. Nevertheless, cumbersome synthetic procedures and loss of atomic-scale control are the serious flaws of these strategies, which hinder the understanding of photocatalysis at the atomic level^21,22^.

The reticular crystalline materials, metal organic frameworks (MOFs) and covalent organic frameworks (COFs), have made great potential in photocatalytic CO_2_ conversion, a frontier in the study of structure-property correlations constructed on the atomic level^23–27^. However, the difficulty to accurately control the structure of the framework material with dual active sites in the in-situ synthesis of crystalline materials may restrict their further development in the conversion of CO_2_. Exploring metal clusters or molecular complexes with defined composition and directionality linked with other metal complexes by coordination linkages or even dynamic covalent linkages might be a promising avenue to overcome these shortcomings^28^. To date, the active sites of oxidative or reductive reactions are only found on a single species (oxidative or reductive homo-metallic clusters) in the reported examples, which is unfavorable for completing the artificial photosynthetic overall reaction^29–32^. If the clusters with water oxidative ability and CO_2_ reductive ability can be connected, it is possible to realize the overall reaction of artificial photosynthesis without additional photosensitizers and sacrificial agents^9,14^. Currently, the mainstream methods to achieve the incorporation of dual active sites with different functions in one single material are by integrating photoactive centers into the structure^33^ or post synthetic modification^20,34^, albeit with some issues, such as cumbersome synthesis and product uncertainty. Therefore, directionally assembling two different clusters with oxidative and reductive abilities via covalent bonds for preparing crystalline porous catalysts may be a brand-new strategy for artificial photosynthesis^35–39^. Due to the stability and solubility issues of different clusters during synthesis and crystallization, almost no reports have been published to realize the construction of cluster-based crystalline porous catalysts through the linkage by covalent bonds.

With the above in mind, to complete the overall reaction directly, a hetero-metallic cluster catalyst, namely MCOF-Ti_6_Cu_3_, has been intentionally constructed with Ti-O clusters with oxidation ability^40,41^ and Cu clusters with reduction capacity^42,43^ for effective coupling photocatalytic CO_2_ reduction and H_2_O oxidation. MCOF-Ti_6_Cu_3_ was prepared by directed assembly through relatively mild dynamic covalent linkages, enabling both rapid electron transfer and synergistic interactions between clusters, without damaging the intrinsic individual components and preserving their oxidative and reductive properties. Under simulated sunlight irradiation, MCOF-Ti_6_Cu_3_ exhibited excellent photocatalytic activity in the generation of HCOOH with a yield of 20.39 μmol in 12 h with O_2_ release, at a high level among the reported non-noble metal photocatalysts with overall reaction. The existence of an internal electric field in MCOF-Ti_6_Cu_3_ with the Ti cluster pointing to the Cu cluster was confirmed by X-ray photoelectron spectroscopy (XPS). Notably, the in-situ XPS and density functional theory (DFT) calculations proved that electrons reach to the Cu cluster under light excitation conditions, and finally the reductive reaction occurs at the Cu cluster and the oxidative reaction at the Ti cluster, which is consistent with the oxidative and reductive centers of the pre-designed material. Finally, in-situ diffuse reflectance infrared Fourier transform spectroscopy (DRIFTS) and DFT calculations unambiguously unveiled the intermediates and mechanism of the photoreaction. This work not only provides a rational strategy for constructing hetero-metallic cluster catalyst, but also an insight into photocatalytic overall reaction by using accurate structural models. Overall, our findings may shed light on the application of crystalline materials in the field of photocatalytic overall reaction for CO_2_ and H_2_O conversion.

## Results and discussion

**Fig. 1:**
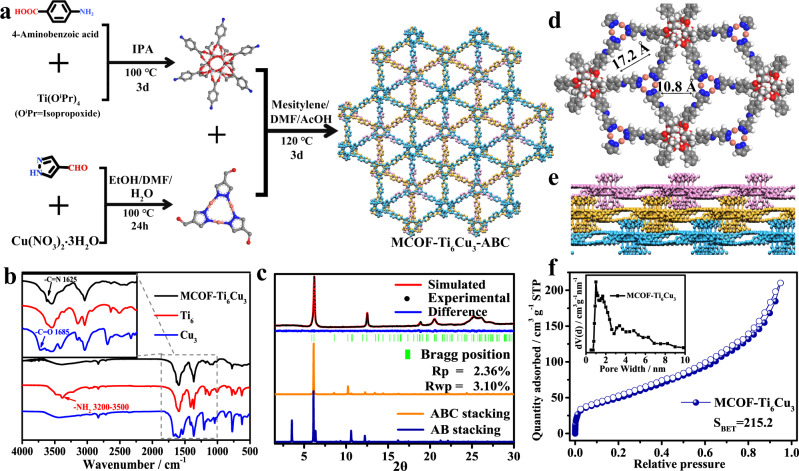
The preparation and characterization. **a** Schematic of the synthesis of MCOF-Ti_6_Cu_3_ via the condensation of **Ti**_**6**_ and **Cu**_**3**_. **b** IR spectra of MCOF-Ti_6_Cu_3_, **Ti**_**6**_ and **Cu**_**3**_. **c** Experimental and simulated PXRD patterns of MCOF-Ti_6_Cu_3_. **d** Top and **e** side views of the structure of MCOF-Ti_6_Cu_3_. Atomic color: Carbon (gray), Nitrogen (blue), Oxygen (red), Copper (orange), Titanium (silver) and Hydrogen (white) **f** N_2_ adsorption curve of MCOF-Ti_6_Cu_3_ at 77 K (inset shows the pore-size distribution profile).

Two precursors [Ti_6_O_6_(O^i^Pr)_6_(AB)_6_] (AB = 4-aminobenzoate; HO^i^Pr = isopropoxide, denoted **Ti**_**6**_)^44^ and [Cu_3_(PyCA)_3_] (1H-PyCA = pyrazolate-4-carboxaldehyde, denoted **Cu**_**3**_)^32^ were prepared according to the previously reported methods. The synthesis of MCOF-Ti_6_Cu_3_ was carried out from the condensation of **Ti**_**6**_ and **Cu**_**3**_ in a mixture of mesitylene and *N, N*-Dimethylformamide (DMF) under the vacuum solvothermal conditions (Fig. 1a). Fourier-transform infrared (FT-IR) spectra confirmed the condensation reaction between the two starting materials by the obvious disappearance of the C = O (1685 cm^−1^) and N-H (3200–3500 cm^−1^) stretching vibration peaks found in **Cu**_**3**_ and **Ti**_**6**_, respectively (Fig. 1b). In addition, a new characteristic peak at 1625 cm^−1^ attributed to the C = N bond was observed, indicating the successful formation of an imine-linked network^45^. Powder X-ray diffraction (PXRD) pattern of MCOF-Ti_6_Cu_3_ displayed the strongest diffraction peak at 2θ 6.1° corresponding to (110) reflection plane, along with minor peaks at 12.4°, 18.8°, 20.4°, and 25.3° that are attributed to the (220), (330), (502), and (440) crystal planes, respectively (Fig. 1c). To elucidate the crystal structure of MCOF-Ti_6_Cu_3_, the staggered stacking model in the *R*_3_ and *P*_63_ space groups corresponding to ABC and AB stacking were built using Materials Studio^28,31,46^. The experimental PXRD pattern matches well with the simulated one for the ABC stacking model. Pawley refinement was applied to provide the refined unit cell parameters of *a* = *b* = 29.14 Å, *c* = 18.97 Å, with good residual factors of *R*_p_ = 2.36% and *R*_wp_ = 3.10%, suggesting the validity of the computational model. The structural information for the AB stacking model is in the Supplementary Information.

These structural analyses reveal that a rhombic channel along *c* axis with a theoretical pore size of 1.08 nm × 1.72 nm and interlayer distance of 5.6 Å (Fig. 1d, e). The porosity and surface area of MCOF-Ti_6_Cu_3_ were examined by N_2_ adsorption-desorption measurements at 77 K (Fig. 1f). The Brunauer-Emmett-Teller (BET) surface area was calculated to be 215.2 m^2^ g^−1^. Besides, the pore size distribution (∼1.01 and ∼1.68 nm) of MCOF-Ti_6_Cu_3_ were in good agreement with the results predicted from the theoretical structure model. The crystallinity and structure of MCOF-Ti_6_Cu_3_ remained by treating it with various solvents at room temperature, which was confirmed by PXRD analyses (Supplementary Fig. 9). The thermal stability of MCOF-Ti_6_Cu_3_ was confirmed by thermogravimetric analysis (TGA) (Supplementary Figs. 10, 11, and 12), which showed no noticeable weight loss up to 300 °C. The morphology of MCOF-Ti_6_Cu_3_ was characterized by scanning electron microscopy (SEM) and transmission electron microscopy (TEM). The SEM and TEM images of MCOF-Ti_6_Cu_3_ shown in Supplementary Figs. 13 and 14 display a sheet-like morphology, which is apparently different from the regular octahedral structure of **Ti**_**6**_ (Supplementary Fig. 15) and the needle-shaped particles of **Cu**_**3**_ (Supplementary Fig. 16). In addition, the high-resolution TEM (HRTEM) image distinctly reveals the high crystallinity of MCOF-Ti_6_Cu_3_ (Supplementary Fig. 17). The corresponding energy-dispersive X-ray spectroscopy (EDS) mapping spectra (Supplementary Fig. 18) indicate that Ti and Cu elements are uniformly distributed in MCOF-Ti_6_Cu_3_.

**Fig. 2:**
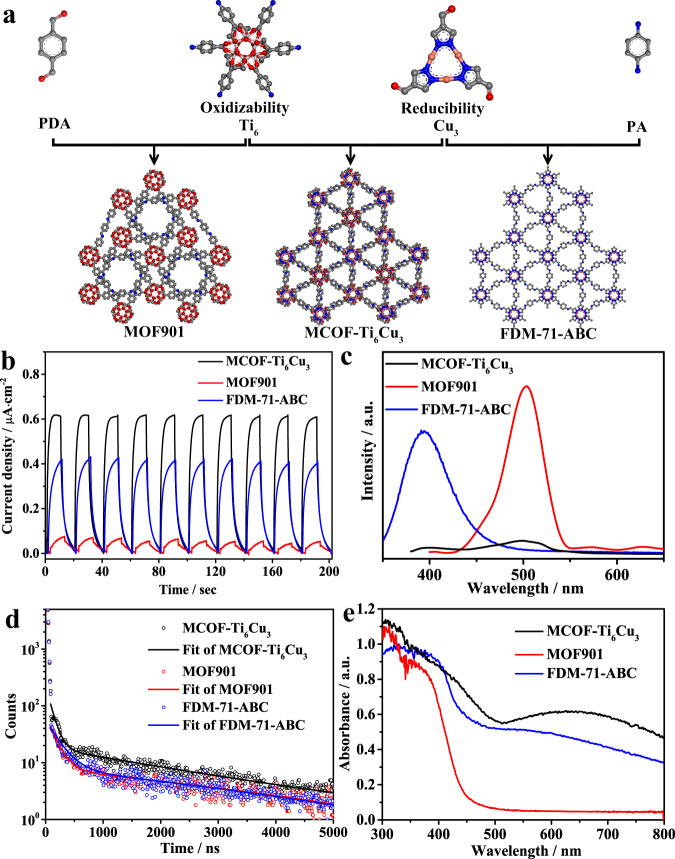
Comparisons for photochemical properties. **a** Schematic of the synthesis of MOF901, FDM-71-ABC, and MCOF-Ti_6_Cu_3_. Photoelectrical Properties of MOF901, FDM-71-ABC, and MCOF-Ti_6_Cu_3_: **b** Transient photocurrent responses. **c** Steady-state PL spectra. **d** Transient PL decay. **e** The solid-state UV-vis DRS.

In order to conduct a more in-depth study on the hetero-metallic cluster catalyst of MCOF-Ti_6_Cu_3_, we also analyzed two monometallic cluster framework materials named MOF901^28^ and FDM-71-ABC^32^ as control samples, which were synthesized from **Ti**_**6**_ with *p*-benzaldehyde and **Cu**_**3**_ with *p*-phenylenediamine, respectively (Fig. 2a, see Supplementary Information for details). For photocatalytic reduction of CO_2_, the separation and migration behavior of photogenerated electron-hole pairs is one of the critical factors. The photoelectro-chemical properties of the samples were performed with the combination of transient photocurrent measurements, electrochemical impedance spectroscopy (EIS) and photoluminescence (PL) spectra. Figure 2b shows that all samples exhibit obvious photocurrent signals and have an excellent reproducibility of the response intensity in the process of on-off cycles. The transient photocurrent intensity of MCOF-Ti_6_Cu_3_ is ∼1.5 times that of FDM-71-ABC and much higher than that of MOF901. Moreover, the photocurrent intensity of **Cu**_**3**_ is about three times as high as **Ti**_**6**_, indicating that the **Cu**_**3**_ cluster may have better photosensitivity (Supplementary Fig. 19). The EIS spectra reveal that compared with MOF901 and FDM-71-ABC, MCOF-Ti_6_Cu_3_ exhibits a smaller semicircle radius in the high-frequency region in Nyquist plots, suggesting a higher separation and transfer efficiency of charge carriers in MCOF-Ti_6_Cu_3_ (Supplementary Fig. 20). As presented in Fig. 2c, the spectral peak position of FDM-71-ABC which originates from **Cu**_**3**_ cluster (Supplementary Fig. 21) is about 400 nm^32^, while that of MOF901 which originates from **Ti**_**6**_ cluster (Supplementary Fig. 22) is about 500 nm^28^. In contrast, MCOF-Ti_6_Cu_3_ exhibits two shifted peaks with the weakest PL intensity, indicating the existence of electron transfer and the lowest recombination efficiency of photoinduced electron-hole pairs. Meanwhile, time-resolved fluorescence decay spectra were measured to determine the specific charge carrier dynamics (Fig. 2d). The results showed that the average lifetime of the photogenerated charge carriers in MCOF-Ti_6_Cu_3_ was 1.90 ± 0.26 μs, which is comparable to that of FDM-71-ABC (1.67 ± 0.09 μs) and longer than that of MOF901 (1.28 ± 0.15 μs). The difference of fluorescence lifetime between MCOF-Ti_6_Cu_3_ and FDM-71-ABC can also be directly evident from the fluorescence decay data (Fig. 2d), which shows that the fluorescence decays with a lower rate in MCOF-Ti_6_Cu_3_ compared with those in the other two samples, illustrating that the photogenerated charge carriers survived longer on the surface of MCOF-Ti_6_Cu_3_. These results further demonstrate that the spatially separated hetero-metallic cluster structures can effectively capture photogenerated electrons and holes respectively, reduce the change of recombination of charge carriers and facilitate their separation and migration in the framework in favor of the higher photocatalytic activity.

**Fig. 3:**
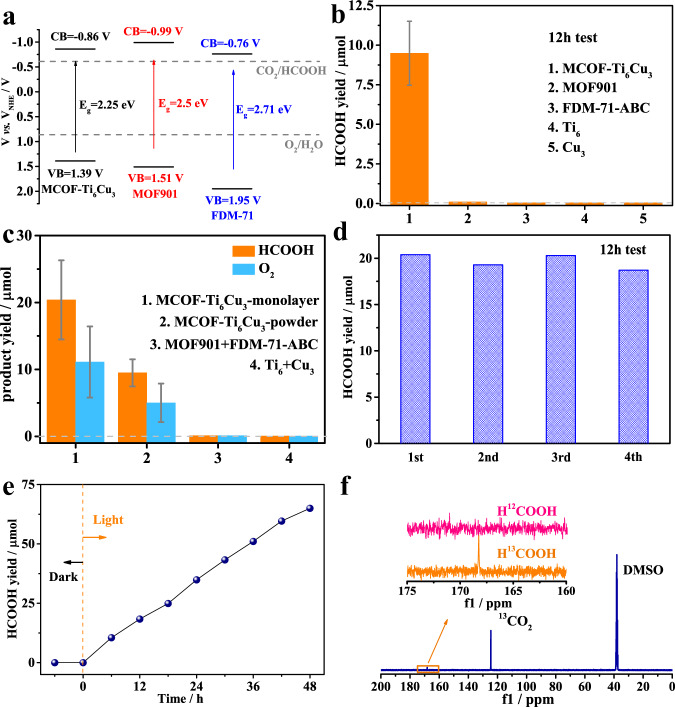
The CO_2_RR performance for various photocatalysts. **a** Band structure diagram of MOF901, FDM-71-ABC and MCOF-Ti_6_Cu_3_. Photocatalysis performances: **b** The comparison of HCOOH yields for different samples. **c** The photocatalytic products yields for samples with different morphology and physical mixtures. **d** Durability measurements (12 h test per cycle). **e** Time-dependent HCOOH production. **f** ^13^C NMR spectra for the photocatalytic reaction solution from ^13^CO_2_ atmosphere. The error bar represents the standard deviation of the measurements.

The solid-state UV-vis diffuse reflectance spectra (UV-vis DRS) of the samples were recorded. As shown in Fig. 2e, FDM-71-ABC and MCOF-Ti_6_Cu_3_ exhibited considerable absorption in the UV and visible regions, whereas the absorption edge of MOF901 was only located near 650 nm. Evaluated by the Tauc plots, the corresponding band gap energies (*E*_g_) of MOF901, FDM-71-ABC, and MOF-Ti_6_Cu_3_ were calculated to be 2.5, 2.71, and 2.25 eV, respectively (Supplementary Figs. 23, 24 and 25). Furthermore, Mott-Schottky measurements were performed to elucidate the semiconductor character of the samples (Supplementary Figs. 26, 27 and 28). The flat band potentials of MOF901, FDM-71-ABC, and MOF-Ti_6_Cu_3_ were determined to be −1.06, −1.19, and −0.96 V *vs*. Ag/AgCl (i.e., −0.86, −0.99, and −0.76 V *vs*. NHE), respectively, which were equal to their conduction band (CB) potentials^47^. Based on the equation *E*_g_ = *E*_VB_ − *E*_CB_, their valence band (VB) positions were accordingly calculated to be 1.39, 1.51, and 1.95 V *vs*. NHE, respectively (Fig. 3a). Considering the CB/VB and LUMO/HOMO, the energy of CB/VB is equivalent to the electrochemical energy potentials of LUMO/HOMO. Obviously, they are thermodynamically suitable for photocatalytic reduction of CO_2_ together with oxidation of H_2_O to various fuels.

The simultaneous completion of CO_2_ reduction and H_2_O oxidation is a great challenge. Therefore, the performance of photocatalytic reduction of CO_2_ with H_2_O over the samples was systematically investigated under simulated sunlight irradiation. As shown in Fig. 3b, the main product of the CO_2_ photocatalytic reduction with MCOF-Ti_6_Cu_3_ was HCOOH, of which the yield reached 9.28 μmol (77.3 μmol g^−1^ h^−1^) after 12 h illumination, while no gaseous products were detected, resulting in a high selectivity. In sharp contrast, MOF901 or FDM-71-ABC showed inactive for CO_2_ reduction, indicating that neither Ti nor Cu cluster could complete the CO_2_ reduction coupled with H_2_O oxidation. In addition, control experiments with **Ti**_**6**_ and **Cu**_**3**_ as the photocatalysts for CO_2_ reduction have also been evaluated under identical conditions. It cloud be found that no HCOOH was observed, indicating that neither the precursor nor homo-metallic cluster catalyst can complete the overall reaction alone. The PXRD pattern and FT-IR suggested structural integrity and no noticeable loss of crystallinity for MCOF-Ti_6_Cu_3_ after reactions, demonstrating its excellent stability (Supplementary Figs. 29 and 30).

The structural units of MCOF-Ti_6_Cu_3_ are clusters, in which interlayer spacing is larger than that of the traditional two-dimensional COFs. Therefore, stripping MCOF-Ti_6_Cu_3_ into single layers is feasible employing high-frequency ultrasound^48^. As shown in Supplementary Fig. 31, after dispersing 10 mg of MCOF-Ti_6_Cu_3_ into 30 mL of deionized water, the nanosheets with the thickness of 4.83 nm were obtained by high-frequency ultrasound for 30 min. The resulting nanosheet structure exhibited a dramatically enhanced yield of HCOOH to 20.39 μmol in 12 h (169.8 μmol g^−1^ h^−1^) (Fig. 3c), which was superior to most of the previously reported noble-metal-free photocatalysts in overall reactions (Supplementary Table 5). Exfoliation has little effects on transient photocurrent responses and electrochemical impedance, but the gas adsorption and specific surface area were greatly improved (Supplementary Figs. 32, 33 and 34). The exfoliation exposes more adsorption sites and active centers, which may be the reason for the excellent photocatalytic performance. The physically mixed control experiments with **Ti**_**6**_ + **Cu**_**3**_ or MOF901 + FDM-71-ABC composites were performed. No products were detected, which demonstrates the necessity of the assembly of MCOF-Ti_6_Cu_3_ in the framework. In addition, in order to evaluate the catalytic stability and reusability of MCOF-Ti_6_Cu_3_, recycling experiments and long-term tests were carried out. As shown in Fig. 3d, MCOF-Ti_6_Cu_3_ retained the original photocatalytic efficiency for the HCOOH production during four consecutive cycles. MCOF-Ti_6_Cu_3_ nanosheets possessed excellent durability during the photocatalytic process, which was further confirmed by the time-dependent conversion yields of CO_2_ into HCOOH, which gradually increased with time (Fig. 3e, Supplementary Fig. 35). The yield of products no longer increased after the removal of catalyst from the reaction, further ruling out the possibility of leaching of Ti and Cu (Supplementary Fig. 36).

Additional control experiments demonstrated that negligible reduction products could be detected without the photocatalyst, CO_2_, or light irradiation. These results suggested that those factors were all indispensable for photocatalytic CO_2_ reduction, and the generated HCOOH originated from CO_2_ rather than the photolysis from other carbon-containing species in this photocatalytic system. In order to further verify the origin of HCOOH, the ^13^CO_2_ isotope trace experiment was performed. The ^13^C NMR spectrum gave the corresponding isotope H^13^COOH signal with a peak at 168.4 ppm, and there was no such signal under the same condition as for ^12^CO_2_ (Fig. 3f), unambiguously verifying that the carbon source of HCOOH indeed originated from the CO_2_ reduction. Alternatively, when H_2_O was replaced with H_2_^18^O, ^18^O_2_ (*m* / *z* = 36), ^18^O^16^O (*m* / *z* = 34) and ^16^O_2_ (*m* / *z* = 32) were detected after the reaction, confirming that the generated ^18^O_2_ or ^16^O_2_ was from H_2_^18^O or H_2_^16^O (Supplementary Fig. 37)^45,49^.

**Fig. 4:**
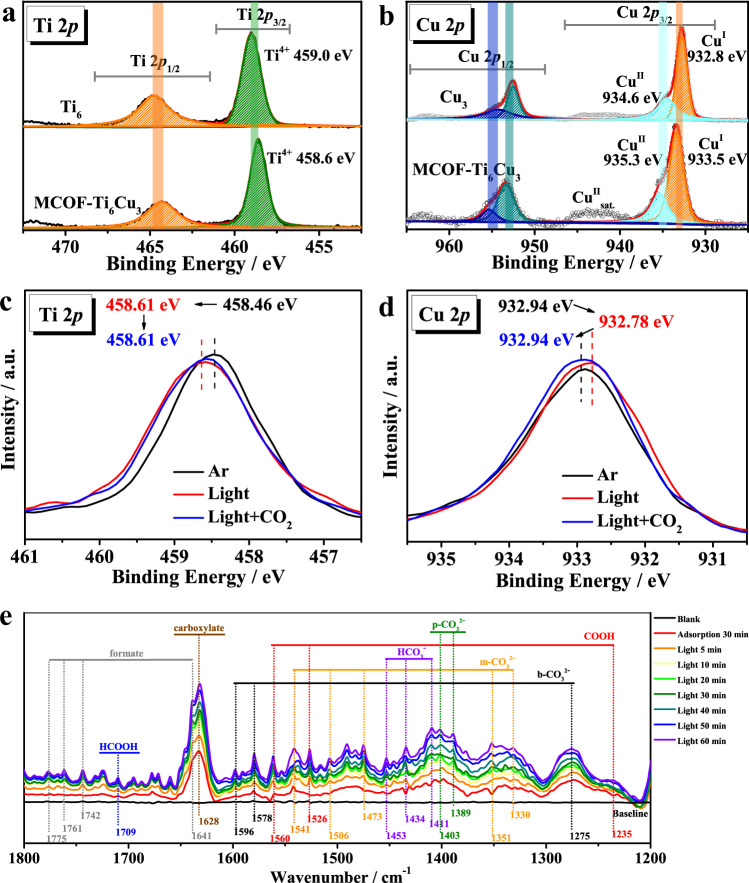
XPS and In-situ measurements. XPS spectra for **a** Ti 2*p* of MCOF-Ti_6_Cu_3_, **Ti**_**6**_ and **b** Cu 2*p* of MCOF-Ti_6_Cu_3_, **Cu**_**3**_. In-situ XPS spectra of **c** Ti 2*p* and **d** Cu 2*p* for the simulated solar-driven CO_2_ reduction process over MCOF-Ti_6_Cu_3._ **e** In-situ DRIFTS spectra for the simulated solar-driven CO_2_ reduction process over MCOF-Ti_6_Cu_3_.

To investigate the mechanism of the photocatalytic overall reaction XPS and in-situ XPS measurements combined with DFT calculations (method in Supplementary Information) were carried out. The calculation model was constructed based on the finite cluster structure of MCOF-Ti_6_Cu_3_ (Supplementary Fig. 38). XPS showed that the observed Ti 2*p* binding energy at 459.0 eV in **Ti**_**6**_ was consistent with Ti^4+^, while the Cu 2*p* binding energy at 932.8 eV and 934.6 eV in **Cu**_**3**_ were assigned to Cu^1+^ and Cu^2+^, respectively (Fig. 4a, b)^18,30^. Previous studies found that the production of Cu^2+^ was due to the surface oxidation, and the Cu valence could be switched between Cu^1+^ and Cu^2+^ without altering the backbone of Cu_3_(pyrazolate)_3_ triangular SBUs^32,50^. For MCOF-Ti_6_Cu_3_, the negative shift of the Ti 2*p* binding energy by 0.4 eV and the positive shift of the Cu 2*p* binding energy by 0.7 eV indicated a successful covalent-bond connection between the two precursors with the formation of an internal electric field, and the possible migration direction of electrons was from Cu to Ti. This interaction from cluster-to-cluster was conducive to the transfer of electrons. The XPS spectra of C, N and O elements are shown in Supplementary Figs. 39, 40 and 41.

The electron transfer process in the photocatalytic reaction could be distinctly revealed by the valence change using in-situ XPS measurements. As shown in Fig. 4c, d, under irradiation (black and red line), the Ti 2*p* binding energy increased from 458.46 eV to 458.61 eV. In comparison, the Cu 2*p* binding energy decreased from 932.94 eV to 932.78 eV, indicating that some electrons returned to Cu cluster with photoexcitation. After CO_2_ was introduced into the system (red and blue line), the binding energy of Cu 2*p* increased to 932.94 eV while that of Ti 2*p* was unchanged, indicating that the transfer direction of the photogenerated electrons was from the Cu cluster to CO_2_. Consequently, it could be inferred that the active center of the photocatalytic reduction of CO_2_ to HCOOH over MCOF-Ti_6_Cu_3_ was the Cu cluster. Finally, as shown in the Supplementary Figs. 42, 43 and 44, the in-situ XPS and in-situ DRIFTS spectra before and after the reaction were consistent, which proves the good stability of the catalyst. As shown in electrostatic potential (ESP) analyses (Supplementary Fig. 45), the positive charge was concentrated on the Cu-N cluster and benzene moieties, while the negative charge was concentrated on the Ti-O cluster.

The DFT and time-dependent DFT (TDDFT) calculation results could also prove that the photocatalytic CO_2_ reduction reaction on the Cu cluster in MCOF-Ti_6_Cu_3_^51,52^. The TDDFT calculation results (Supplementary Fig. 46) show that the HOMO and LUMO of MCOF-Ti_6_Cu_3_ are close to each other and can be excitated by the excitation energy of infrared light (1704.16 nm). However, visible and ultraviolet lights correspond to multiple excitation modes. The visible lights with wavelengths of 487.42 nm and 453.25 nm corresponded to the excitation modes of HOMO → LUMO + 27, + 29, + 31, and HOMO → LUMO + 31, + 32, while the wavelengths of 352.79 nm and 351.89 nm in ultraviolet region correspond to the excitation modes of HOMO − 3 → LUMO + 1 and HOMO  1 → LUMO + 1, respectively. The orbital composition analysis displayes that the occupied orbitals of HOMO, − 1, and − 3 are located on the Cu cluster fragments, and the unoccupied orbitals of LUMO, + 27, + 29, + 31, and + 32 are also located on the Cu cluster fragments, other unoccupied orbitals are located on the Ti cluster fragments. It demonstrates that the electron cloud does not transfer in the process of infrared excitation, and indeed transfers from the Cu cluster to the Ti cluster under ultraviolet excitation. The electron cloud is distributed on the Cu cluster under visible excitation, which corresponds to the result that the Cu cluster receives electrons under optical excitation from the in-situ XPS measurement.

To further clarify the reasons for the excellent photocatalytic activity and HCOOH conversion selectivity, in-situ DRIFTS spectroscopy was performed to probe the intermediates^53–58^. As shown in Fig. 4e, new peaks obviously appeared and their intensities gradually increased with the illumination time from 0 to 60 min. The absorption peaks located at 1596, 1578, and 1275 cm^−1^ belong to bidentate carbonate (b-CO_3_^2−^), and the peaks at 1541, 1506, 1473, 1351, and 1330 cm^−1^ are attributed to monodentate carbonate (m-CO_3_^2−^). Additionally, the peaks at 1389 and 1403 cm^−1^ are assigned to polydentate carbonate (p-CO_3_^2−^), and the peaks at 1453, 1434, and 1411 cm^−1^ are ascribed to bicarbonate (HCO_3_^−^). The peak intensities of these carbonates increase significantly, which suggests that MCOF-Ti_6_Cu_3_ can chemically adsorb and interact with CO_2_ and H_2_O molecules. The peak at 1641 cm^−1^ and the emerging peaks at 1742, 1761, and 1775 cm^−1^ maybe result from a formate, and the peak at 1628 cm^−1^ could be attributed to carboxylate. Meanwhile, the intensities of the peaks at 1560, 1526, and 1235 cm^−1^ corresponding to *COOH increased with the prolonged illumination time, which might be possibly caused by the favorable proton capture capability of CO_2_^•−^ radicals. Most importantly, the intensity of the peak at 1709 cm^−1^ gradually increased with light progresses^53,57^. This peak is ascribed to *HCOOH which are important intermediates for the formation of HCOOH.

**Fig. 5:**
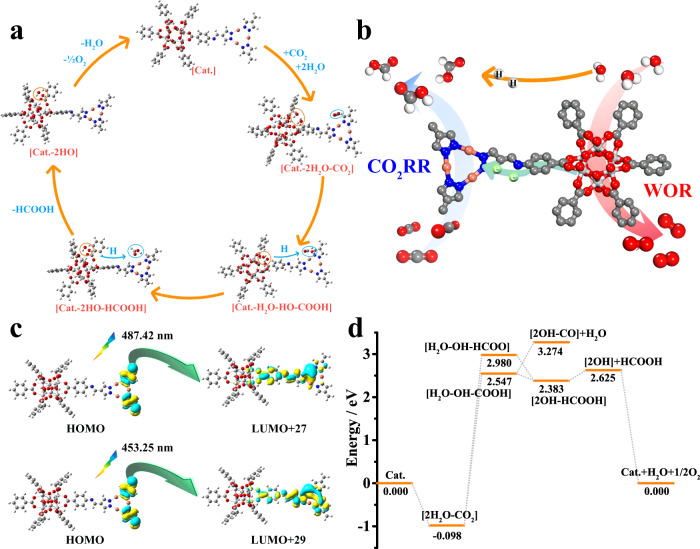
Mechanism and DFT calculations. **a** A proposed reaction pathway for the photocatalytic CO_2_ reduction over MCOF-Ti_6_Cu_3_. **b** Proposed mechanism of CO_2_ reduction and H_2_O oxidation. **c** Transitions of electron cloud distribution under solar irradiation. **d** Free-energy profile for the CO_2_ reduction and H_2_O oxidation pathway over MCOF-Ti_6_Cu_3_.

According to the results of in-situ DRIFTS analysis and DFT calculation, the possible mechanism of CO_2_ reduction and H_2_O oxidation over the hetero-metallic cluster catalyst MCOF-Ti_6_Cu_3_ can be reasonably proposed (Fig. 5a). The specific calculation models are in Supplementary Figs. 47, 48 and 49. We conjectured that the Cu cluster unit could be the active site for CO_2_ reduction, while the adsorption and activation of H_2_O were at the Ti cluster (Fig. 5b). To be specific, under irradiation, the photogenerated electrons and holes separated and transferred to the Cu cluster and Ti cluster, respectively. Then reduction and oxidation reactions took place simultaneously on these two clusters (Fig. 5c). On the Cu cluster, the conversion of CO_2_ to HCOOH requires two consecutive hydrogenation steps. The first step is the conversion of CO_2_ into *COOH, and then the formed *COOH undergoes a second reaction to generate HCOOH. Correspondingly, the oxidation of H_2_O at the Ti cluster involves twice the conversion processes of H_2_O into OH•. Finally, when HCOOH is desorbed from the system, the two OH• radicals combine with each other to generate O_2_ and H_2_O, thus maintaining the charge balance of the catalytic system. As shown in Fig. 5d, the formation of *COOH and OH• from CO_2_ and H_2_O is the rate determining step in the process of the photocatalytic overall reaction. In addition, two intermediates, *COOH and HCOO*, are generated in the first hydrogenation reaction. The *COOH is preferentially generated since the energy of the formation of *COOH is lower than that of HCOO*. The energy of *COOH dehydration into CO is much higher than that of *COOH hydrogenation into HCOOH, accounting for the preferential generation of HCOOH among the reduction products, which is consistent with the experimental results.

In summary, in order to realize an effective coupled photocatalytic oxidative and reductive overall reaction, we have elaborately designed and constructed a hetero-metallic cluster catalyst via linkage of dynamic covalent bonds, namely MCOF-Ti_6_Cu_3_, in which functional clusters with reductive and oxidative activities can cooperate for photocatalytic CO_2_ reduction and water oxidation, respectively. In addition, hetero-metallic cluster catalyst obtained by directed assembly inherits the oxidative and reductive properties of the clusters and achieves spatial separation. Also, the dynamic covalent bonds have more efficient electron transfer, enabling MCOF-Ti_6_Cu_3_ to exhibit excellent photocatalytic performance with a high HCOOH yield of 169.8 μmol g^−1^ h^−1^ in crystalline materials with only H_2_O. More importantly, the in-situ tests and DFT calculations prove that the reduction reaction occurs on the Cu cluster and the oxidation reaction on the Ti cluster, which is consistent with our pre-designed oxidative and reductive centers on hetero-metallic cluster catalyst, as well as distinctly reveals the charge transfer path and overall photocatalytic reaction mechanism on the atomic level. This research provides a strategy for the construction of hetero-metallic cluster catalyst from the chemical cooperation of MOF **∪** COF, and offers a significant guideline for the design of the photocatalytic overall reaction systems.

## Methods

### Materials

The starting materials for COF syntheses were purchased from Shanghai Tensus Bio-tech Co., Ltd. Other reagents and solvents applied in the synthesis and photocatalysis were purchased from Aladdin and Sigma-Aldrich, and used as received without further pretreatments.

### Synthesis of MCOF-Ti_6_Cu_3_

**Ti**_**6**_ (31.1 mg, 0.02 mmol) and **Cu**_**3**_ (19.8 mg, 0.04 mmol) were mixed in a cylindrical glass tube (20 cm of length, ф_in_ = 0.8 cm, ф_out_ = 1.0 cm) to which were added mesitylene, DMF and glacial acetic acid (6 M, 9:1:1, *v*/*v*/*v*). Then the mixture was sonicated for 15 min to form a homogenous dispersion. After degassed by three freeze-pump-thaw cycles, the tube was sealed off and then heated at 120 °C for 3 days. The bright yellow precipitates (turn green in air) were collected by filtration and soaked in DMF for 24 h. The powder was transferred to a Soxhlet extractor and washed with THF (24 h). Finally, the product was evacuated at 120 °C under vacuum overnight to yield the activated sample.

### Photocatalytic experiment

The photocatalytic experiments were carried out following the reported method^45^. In a quartz reactor (50 mL), the catalyst (10 mg) was dispersed in ultra-pure water (30 mL). This mixture system was bubbled with pure CO_2_ gas for 20 min. The temperature of the reaction solution was maintained at 25 °C controlled by an outside flow of water during the reaction. Then, the system was irradiated under simulated sunlight using a PLS-SXE300 Xe lamp with an AM 1.5 cut-off filter.

The liquid products in liquid phase of the reactor were measured using ionic chromatography (IC) to analyze HCOOH. The gaseous products were analysed by the gas chromatography. Detailed methods are in the Supplementary Information.

### Electrochemical measurements

Photocurrent, electrochemical impedance spectroscopy and Mott-Schottky measurements were all tested using the following methods^45,47^. Electrochemical measurements of MOF901, FDM-71-ABC and MCOF-Ti_6_Cu_3_ were tested in a three-electrode electrochemical workstation system (CHI 660e) with the ITO glass substrate (1 cm × 1 cm) as the working electrode, the Pt-wire as the auxiliary electrode and Ag/AgCl electrode as the reference electrode. The samples (2 mg) were dispersed into the solution (1 mL) containing Nafion solution (100 μL, 0.5 wt%), water (450 μL), and ethanol (450 μL). After sonication, the spreading aqueous slurries were drop-cast onto the ITO glass substrate. Then, the working electrode was dried spontaneously under ambient temperature. 0.2 M Na_2_SO_4_ solution was used as the electrolyte. Irradiation was carried out by using a 300 W xenon lamp with AM 1.5 cut-off in the photocurrent measurement.

### Computational methods

The calculation model is constructed by a finite cluster structure, and all calculations were performed with the Gaussian 16 software package. The ground state of MCOF-Ti_6_Cu_3_ was geometrically optimized by using B3LYP method of DFT with Ti, Cu/LanL2dz, C, H, N, O/6-31 G* basis sets^51,52^. ESP analysis, TDDFT calculations, and orbital composition were also performed at the same level. In order to obtain the corresponding excited state energies, TDDFT calculations were performed on the basis of optimized ground state configurations. The energy of [MCOF-Ti_6_Cu_3_] is specified as 0.000 eV, and the ∆*E* (eV) calculations for the other states are shown in Supplementary Tables 6 and 7.

## Supplementary information

Supplementary Information

## Data Availability

The data that support the findings of this study are available within the paper and its supplementary information files or are available from the corresponding authors upon reasonable request. Source data are provided with this paper.

## Source data

Source Data
