# Supplementary material for: Linking oxidative and reductive clusters to prepare crystalline porous catalysts for photocatalytic CO2 reduction with H2O
Source: Nat Commun. 2022 Aug 10;13:4681. doi: 10.1038/s41467-022-32449-z (PMC9365760; doi:10.1038/s41467-022-32449-z)
Supplement: Supplementary file 1 — Supplementary Information [file 41467_2022_32449_MOESM1_ESM.pdf]

Supplementary Information

Linking Oxidative and Reductive Clusters to Prepare Crystalline Porous Catalysts for Photocatalytic CO_2 reduction with H_2O

Jie Zhou[1†], Jie Li[1†], Liang Kan[1], Lei Zhang[1], Qing Huang[1], Yong Yan[1*], Yifa Chen[1], Jiang Liu[1], Shun-Li Li[1], Ya-Qian Lan[1*]

1 School of Chemistry, South China Normal University, Guangzhou, 510006, P.R. China.

†Jie Zhou, Jie Li contributed equally to this work.

*Correspondence and requests for materials should be addressed to Y.-Q. L. (email: yqlan@m.scnu.edu.cn);

Y. Yan (email: Yong.Yan@m.scnu.edu.cn).

Homepage : http://www.yqlangroup.com/

Supplementary Figures

Synthesis of [$Ti_6O_6(OiPr)_6(AB)_6$] (Ti_6)

The hexameric [$Ti_6O_6(OiPr)_6(AB)_6$] (where OiPr = isopropoxide; AB = 4-aminobenzoate) cluster was synthesized according to previously reported procedures[1]. To a 2-propanol solution (6.0 mL) containing 4-aminobenzoic acid (192.1 mg, 1.40 mmol), titanium (IV) isopropoxide (103.6 μL, 0.35 mmol) was added. After stirring for 30 min at RT, the orange-colored slurry was heated to 100 °C for 72 h inside a sealed glass tube. The bright yellow crystalline product was collected, washed with 2-propanol and dried under vacuum for 3 h (61.4 mg, 68%).

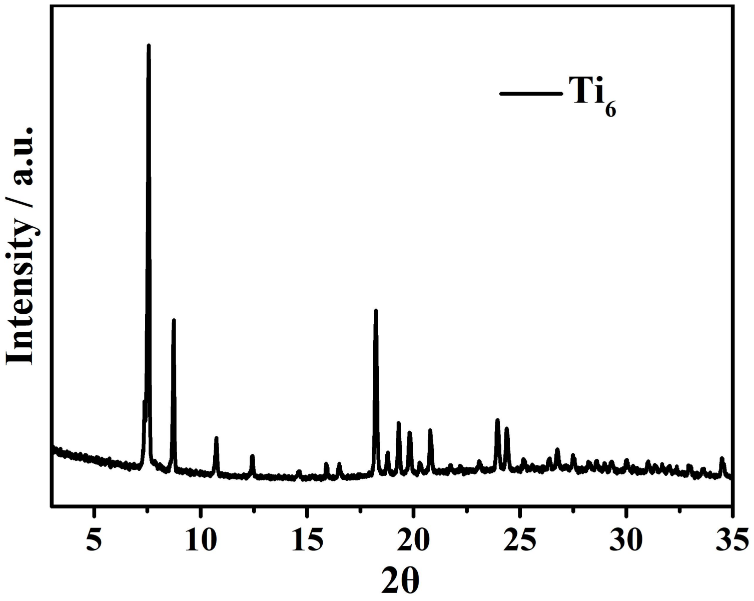

Supplementary Figure 1. PXRD pattern of [$Ti_6O_6(OiPr)_6(AB)_6$] (**Ti_6**).

Synthesis of [$Cu_3(PyCA)_3 \cdot H_2O$] (Cu_3)

The Trimeric [$Cu_3(PyCA)_3 \cdot H_2O$] (where PyCA = 1H-pyrazole-4-carbaldehyde) cluster was synthesized according to previously reported procedures[2]. $Cu(NO_3)_2 \cdot 3H_2O$ (0.20 g, 0.83 mmol) and 1H-pyrazole-4-carbaldehyde (HPyCA, 0.096 g, 1.0 mmol) were dissolved in a mixture of 6.7 mL *N, N*-dimethylformamide (DMF), 5.0 mL H_2O, and 6.7 mL ethanol in a 25 mL vial. The tightly capped vial was placed in an oven at 100 °C for 12 h, and light yellow single crystals were obtained. The crystals were collected and immersed in H_2O for 3 days, during which time H_2O was exchanged three times per day. Afterwards, the crystals were quickly washed with acetone for three times, followed by drying under vacuum at 120 °C for 24 h. Yield: 66%.

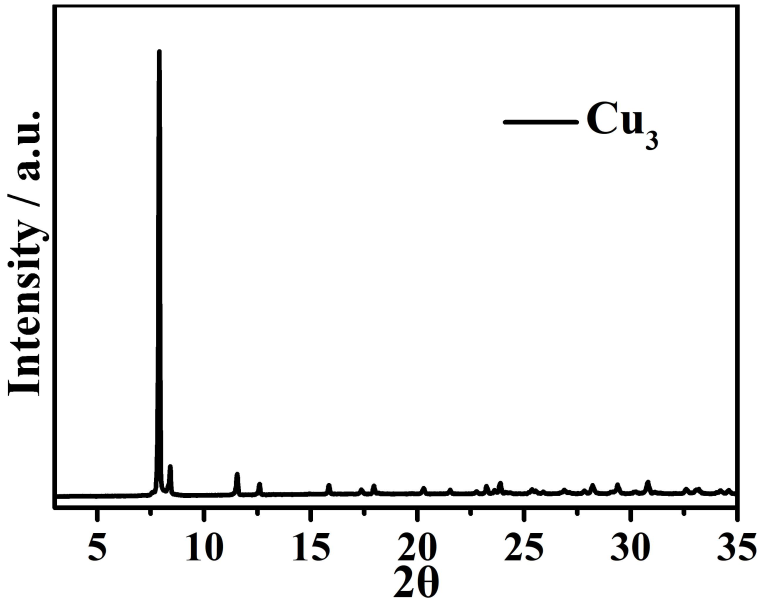

Supplementary Figure 2. PXRD pattern of [$Cu_3(PyCA)_3 \cdot H_2O$] **(Cu_3)**.

Structure information of MCOF-Ti_6Cu_3.

We modeled this structure as AB stacking mode according to MOF901[3] and ABC stacking mode according to refs 4 and 5. By using Materials Studio, the staggered stacking model in the R_3 and P_{63} space groups corresponding to ABC and AB stacking were built. The results show that the PXRD pattern of MCOF-Ti_6Cu_3 matches very well with its ABC stacking. According to the simulated ABC stacking structure and Pawley refinement, the observed peaks at 2θ 6.1°, 12.4°, 18.8° and 25.3° can be indexed to (110), (220), (330) and (440) Miller indices respectively, which further confirms that the MCOF-Ti_6Cu_3 structure adopts the ABC packing mode.

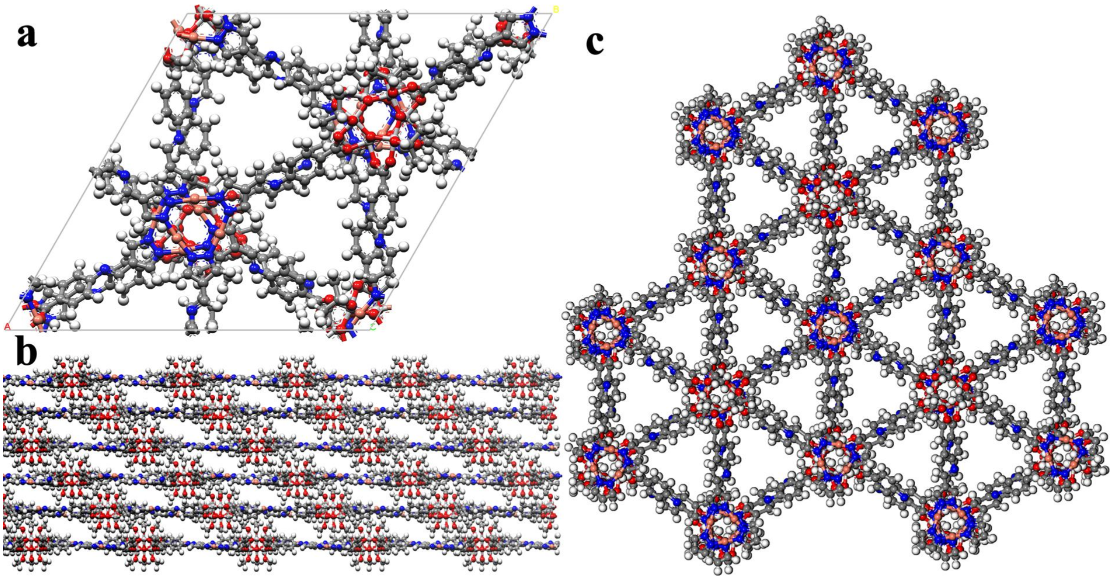

Supplementary Figure 3. Space filling models. The view of ABC stacking of MCOF-Ti_6Cu_3 from the **a** *ab* plane, **b** side view and **c** top view. Carbon (grey), Nitrogen (blue), Oxygen (red), Copper (orange), Titanium (silver) and Hydrogen (white).

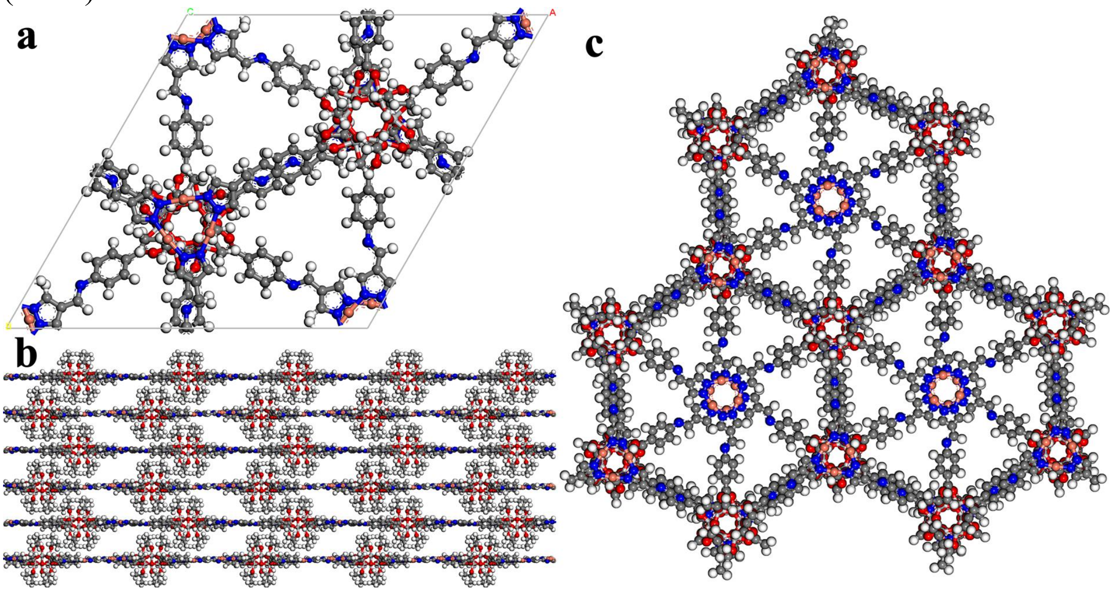

Supplementary Figure 4. Space filling models. The view of AB stacking of MCOF-Ti_6Cu_3 from the **a** *ab* plane, **b** side view and **c** top view. Carbon (grey), Nitrogen (blue), Oxygen (red), Copper (orange), Titanium (silver) and Hydrogen (white).

Synthesis of MOF-901

The MOF-901 was synthesized according to previously reported procedures[3]. Benzene-1,4-dialdehyde (10.0 mg, 0.075 mmol) was dissolved in 3 mL of methanol and then dropped into a mixture of **Ti_6** (38.9 mg, 0.025 mmol). The solution was then introduced to a cylindrical glass tube (20 cm of length, ϕ_{in} = 0.8 cm, ϕ_{out} = 1.0 cm). The tube was flash frozen at 77 K using a liquid nitrogen bath, evacuated for 10 min in order to remove oxygen, and then flamed seal to maintain a vacuum during the reaction process. After degased by three freeze-pump-thaw cycles, the reaction mixture was left at 125 °C for 3 days. The yellow crystalline powder was collected and washed with DMF (5 × 5 mL, each day) for removal of **Ti_6**. The sample was then soaked in anhydrous dichloromethane for 3 d (3 × 20 mL, each day). The sample was collected after evacuating at 120 °C for 24 h.

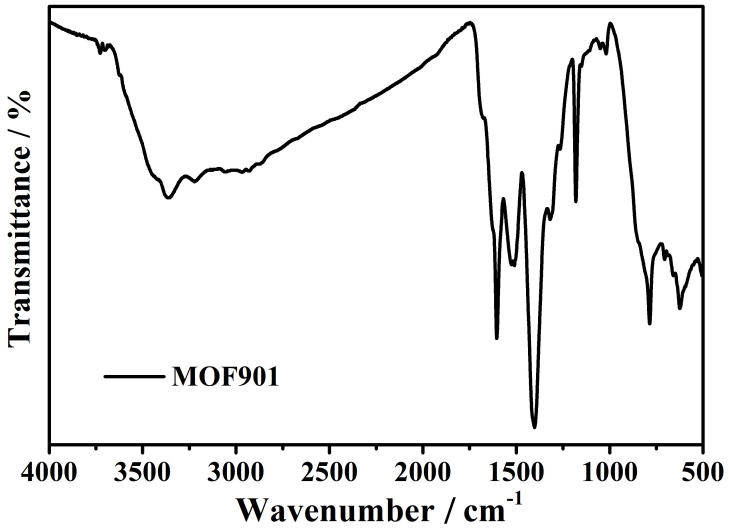

Supplementary Figure 5. FT-IR spectrum of MOF901.

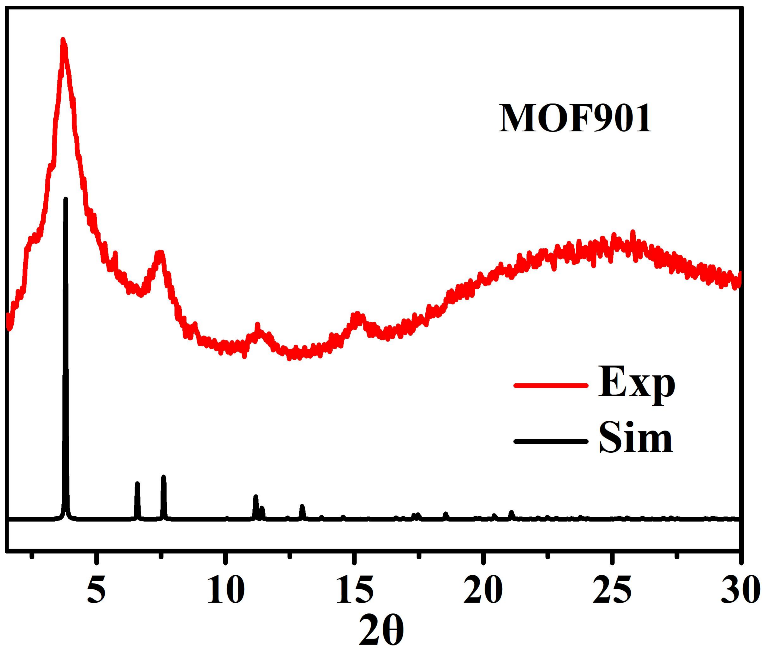

Supplementary Figure 6. PXRD pattern of MOF901.

Synthesis of FDM-71-ABC.

The FDM-71-ABC cluster was synthesized according to previously reported procedures with some modifications.

FDM-71 has been reported to be AA stacking[2]. According to another report with **Cu_3**[4], different reaction systems will directly affect the final stacking structure. The catalytic activity for the ABC stacking will be better than that for the AA stacking, because there are more active sites exposed. Meanwhile, the stacking mode of MCOF-Ti_6Cu_3 is also ABC stacking. Therefore, we optimized FDM-71-ABC according to the synthesis conditions of JNM-3 and obtained its ABC stacking structure. The specific methods are as follows:

Phenylenediamine (PA, 11 mg, 0.1 mmol) and $Cu_3(PyCA)_3{\cdot}H_2O$ crystals (34 mg, 0.07 mmol), 0.5 mL of mesitylene, 0.5 mL of 1,4-dioxane and 0.1 mL of 6 M aqueous acetic acid were added in a cylindrical glass tube (20 cm of length, ϕ_{in} = 0.8 cm, ϕ_{out} = 1.0 cm). The mixture was sonicated for 15 minutes to get a homogenous dispersion. And then the tube was flash frozen at 77 K in liquid nitrogen bath and degassed with three freeze-pump-thaw cycles. Upon warming to room temperature, the mixture was heated at 120 °C for 72 h. The yellow crystalline powder was collected and washed with DMF (5 × 5 mL, each day) for removal of **Cu_3**. And then, the powder was transferred to a Soxhlet extractor and washed with THF (24 hours). Finally, the sample was collected after evacuating at 120 °C for 12 h.

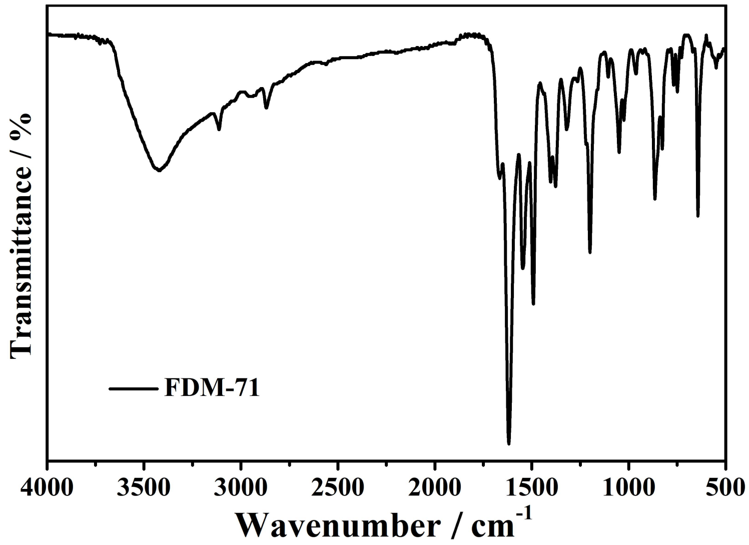

Supplementary Figure 7. FT-IR spectrum of FDM-71-ABC.

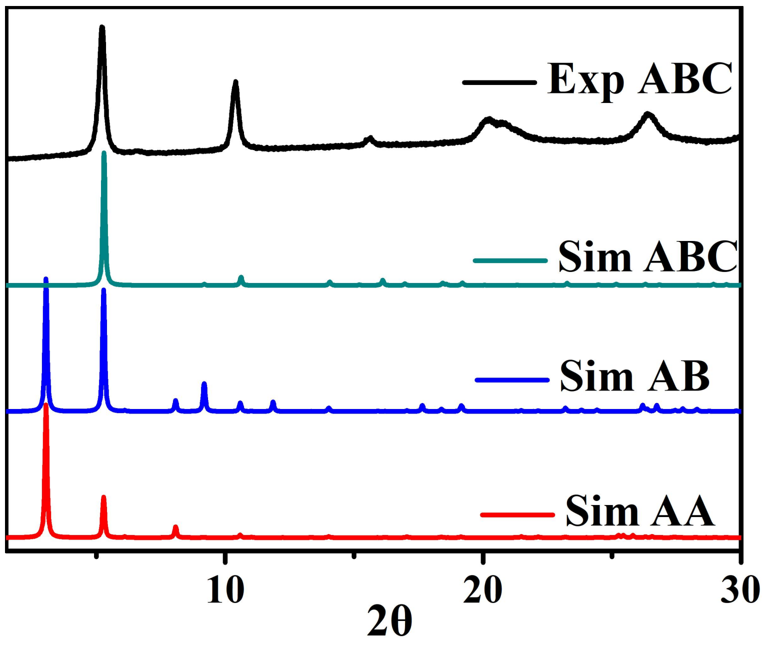

Supplementary Figure 8. PXRD pattern of FDM-71-ABC.

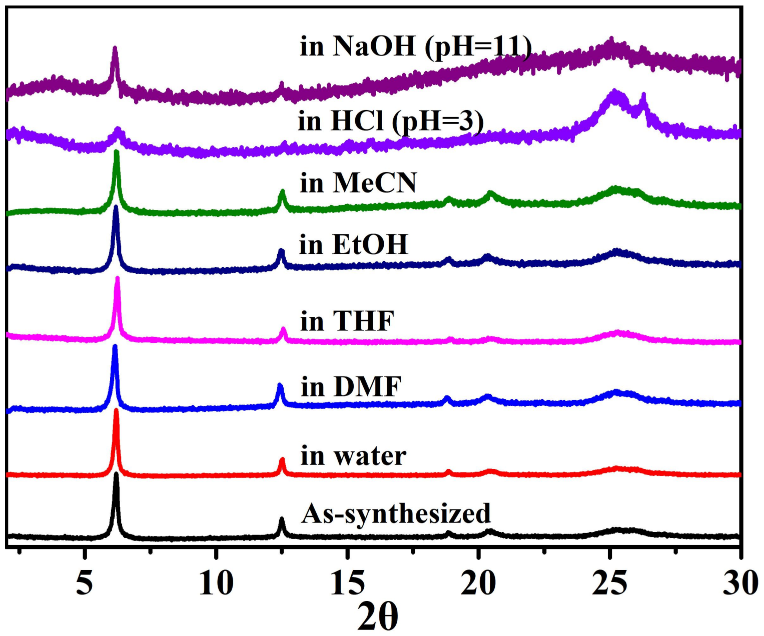

Supplementary Figure 9. PXRD pattern of MCOF-Ti_6Cu_3 after immersed in different solvents for 3 days. The pH value of photocatalytic system was 4.18 ± 0.05.

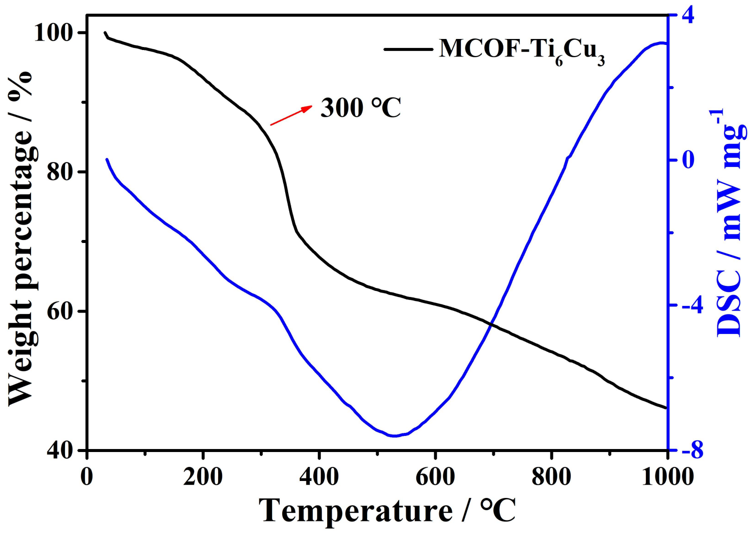

Supplementary Figure 10. TGA and DSC curves of MCOF-Ti_6Cu_3 under N_2 atmosphere.

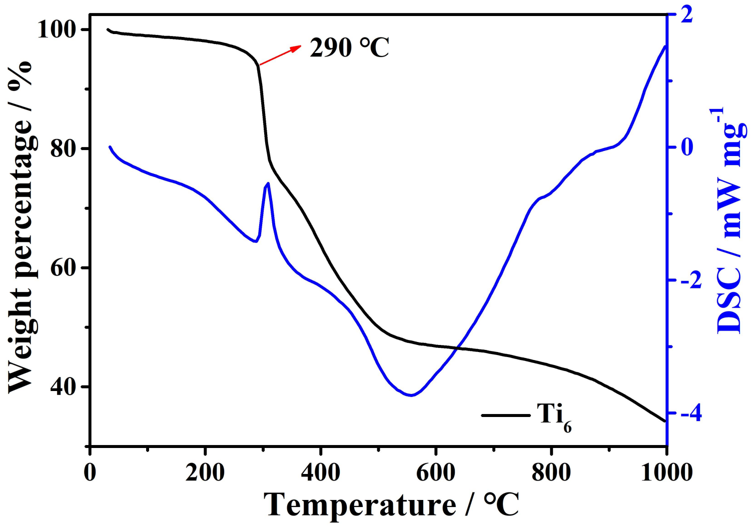

Supplementary Figure 11. TGA and DSC curves of **Ti_6** under N_2 atmosphere.

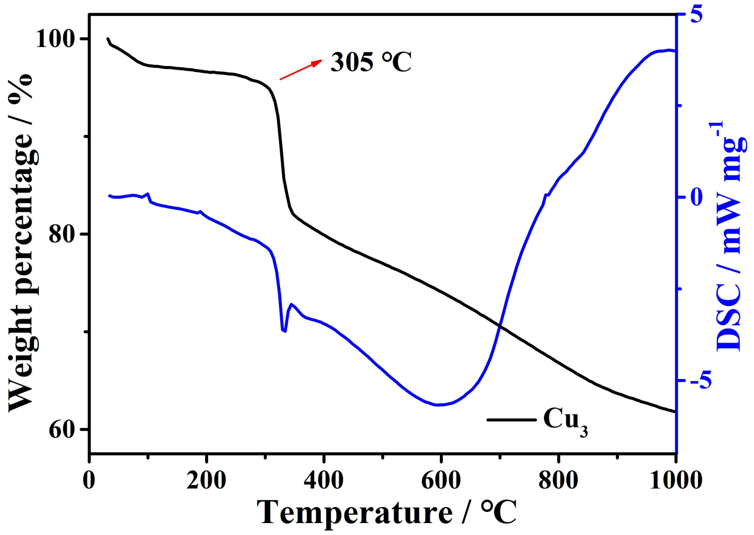

Supplementary Figure 12. TGA and DSC curves of **Cu_3** under N_2 atmosphere.

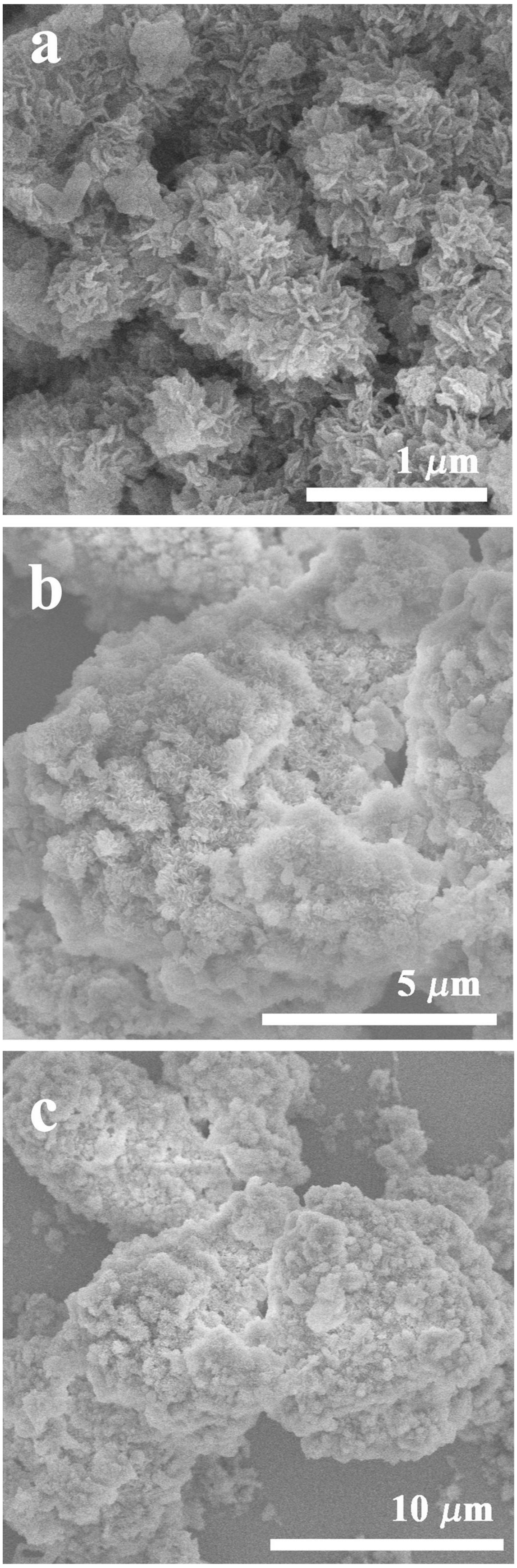

Supplementary Figure 13. SEM images of MCOF-Ti_6Cu_3. **a** 1 μm, **b** 5 μm and **c** 10 μm.

stripping.

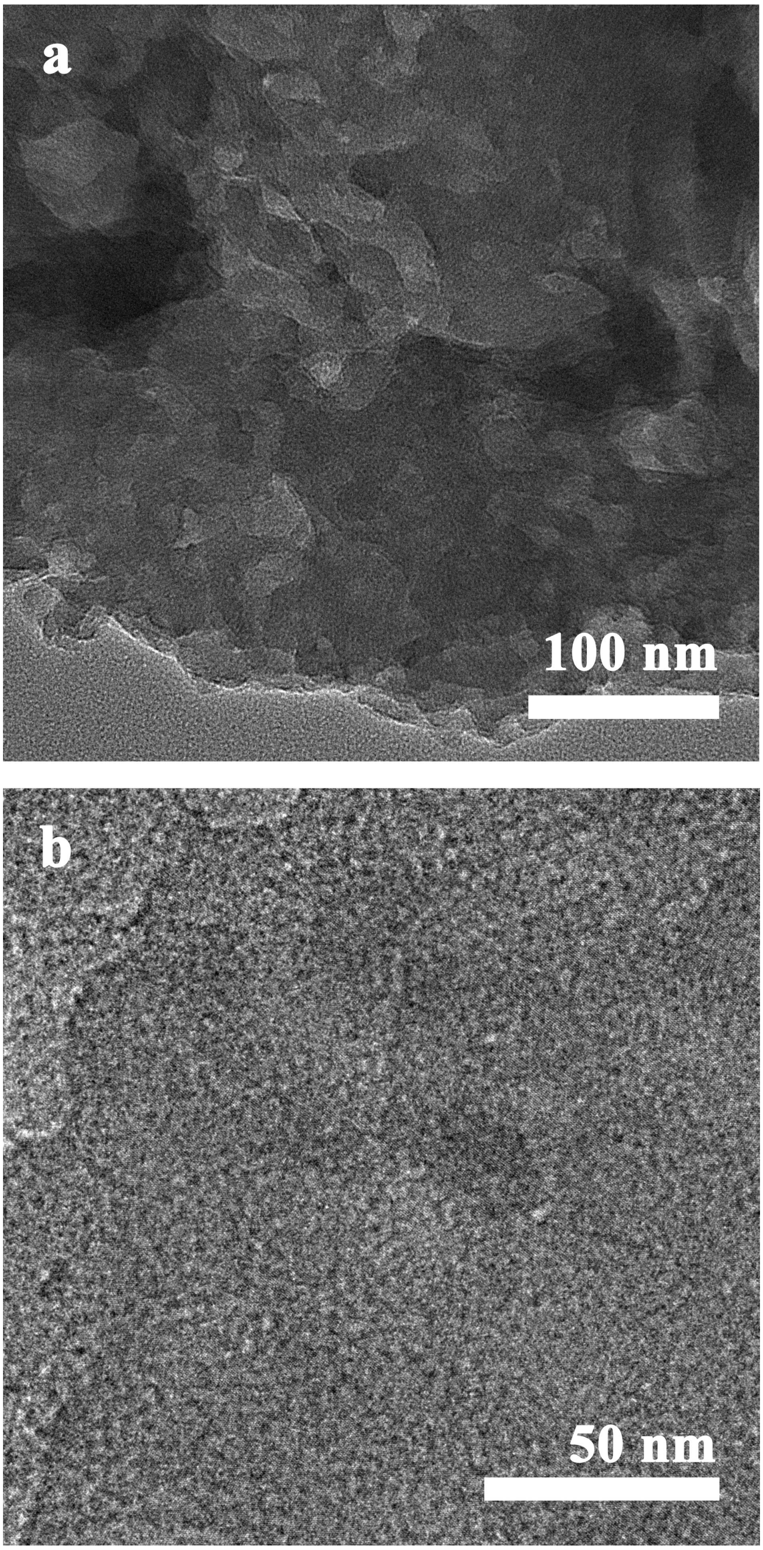

Supplementary Figure 14. TEM images of MCOF-Ti_6Cu_3. **a** Before and **b** after stripping.

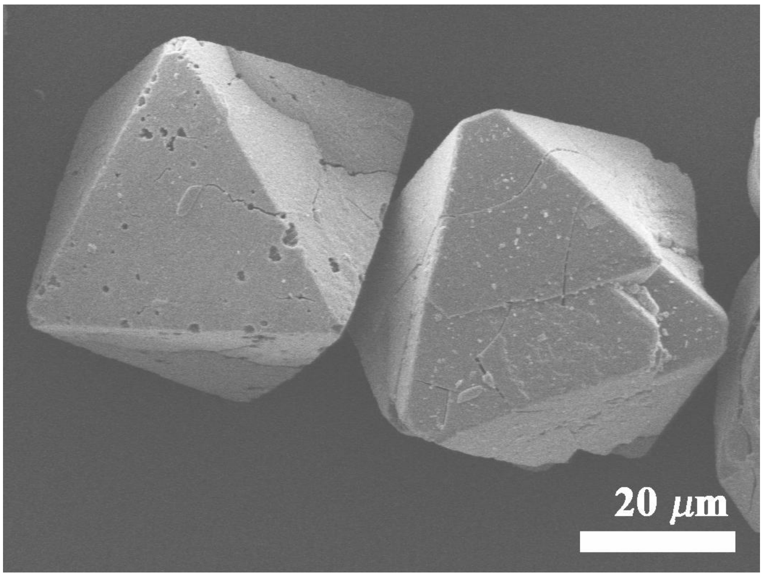

Supplementary Figure 15. SEM image of **Ti_6**.

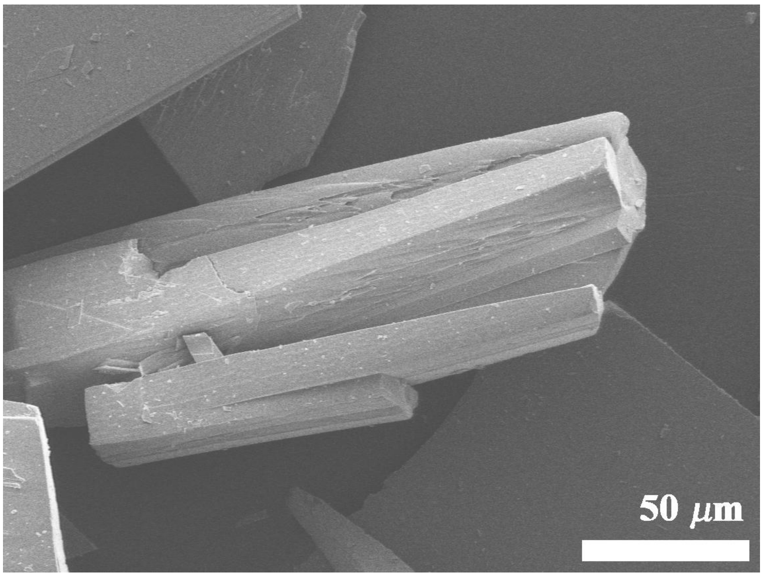

Supplementary Figure 16. SEM image of **Cu_3**.

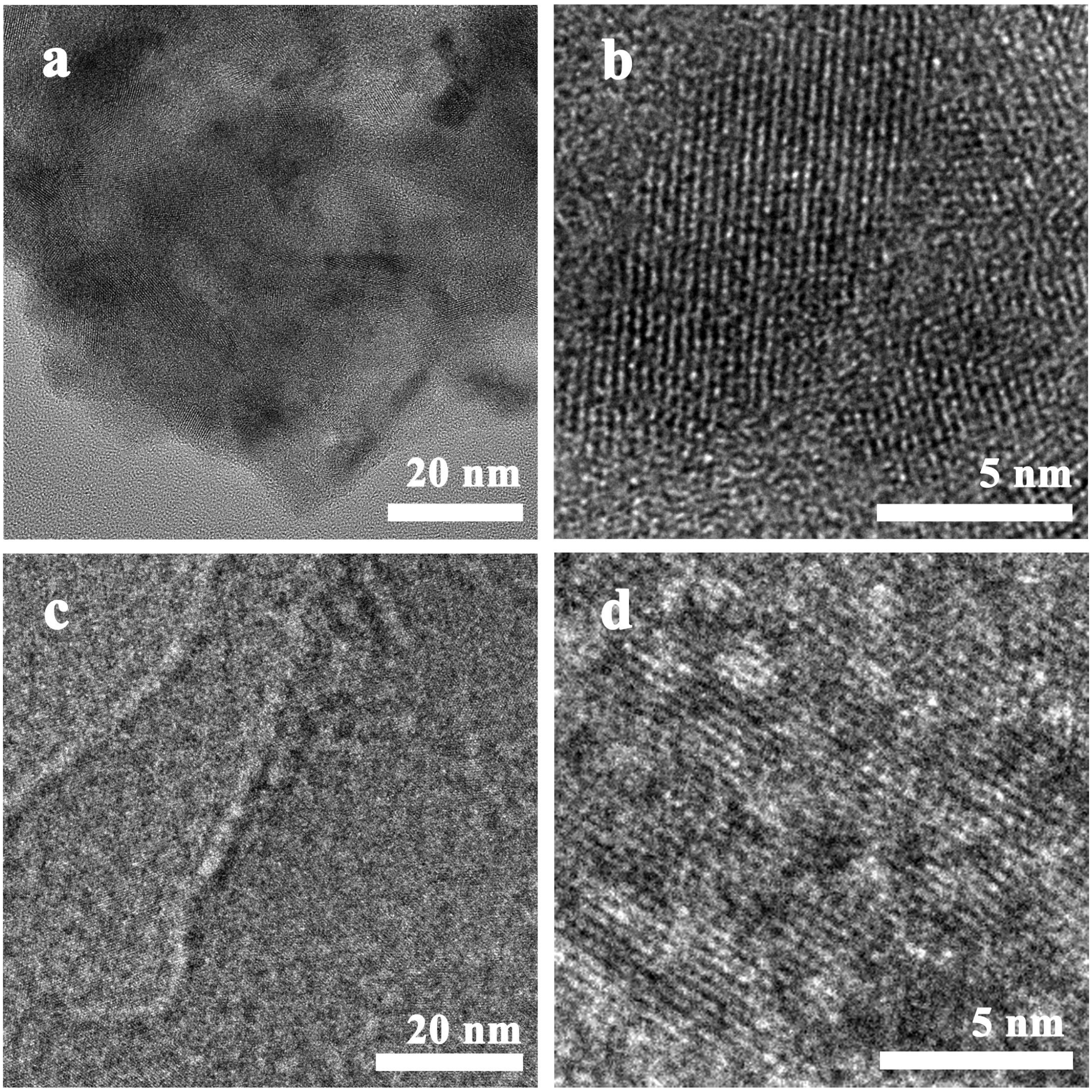

Supplementary Figure 17. HRTEM images of MCOF-Ti_6Cu_3. **a, b** Before and **c, d** after stripping. Among them, **b** and **d** are the enlarged views of **a** and **c**, respectively.

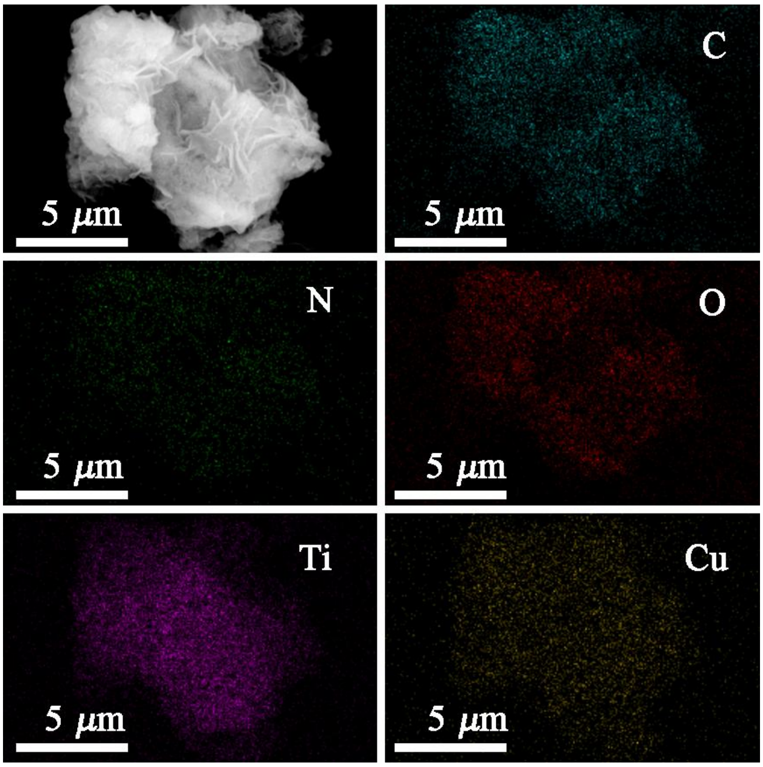

Supplementary Figure 18. EDS image of MCOF-Ti_6Cu_3.

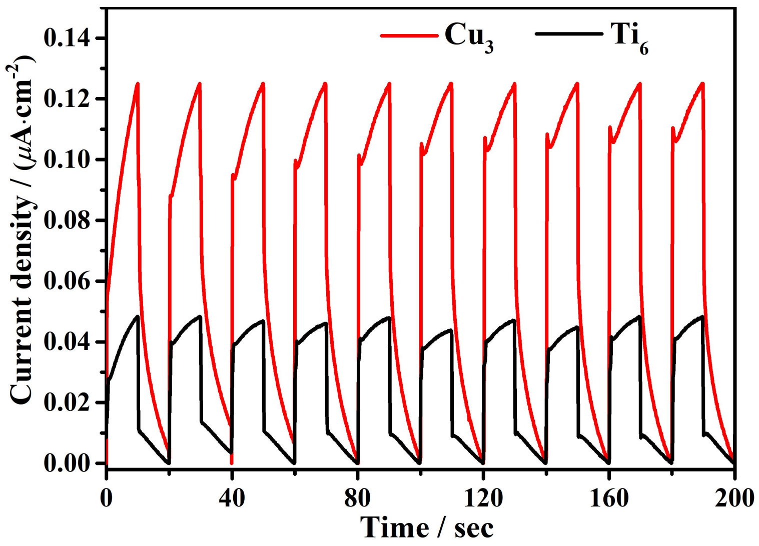

Supplementary Figure 19. Transient photocurrent responses image of **Ti_6** and **Cu_3**.

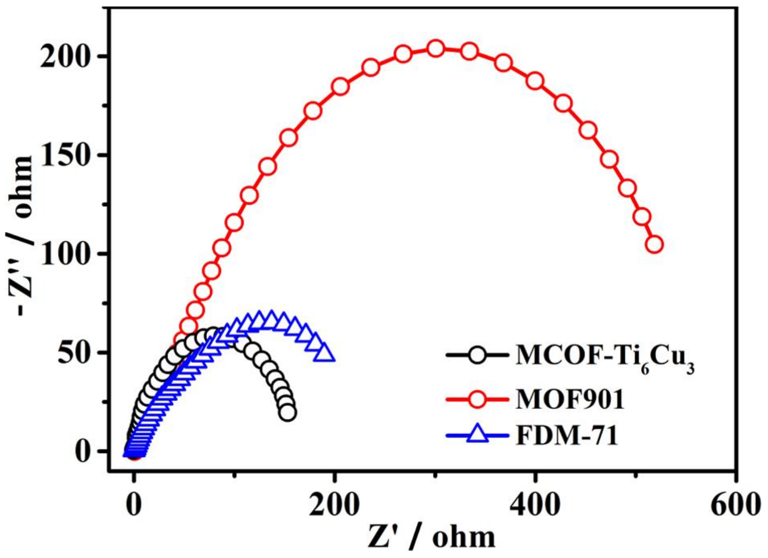

Supplementary Figure 20. Electrochemical impedance spectroscopy of MOF901, FDM-71-ABC and MCOF-Ti_6Cu_3.

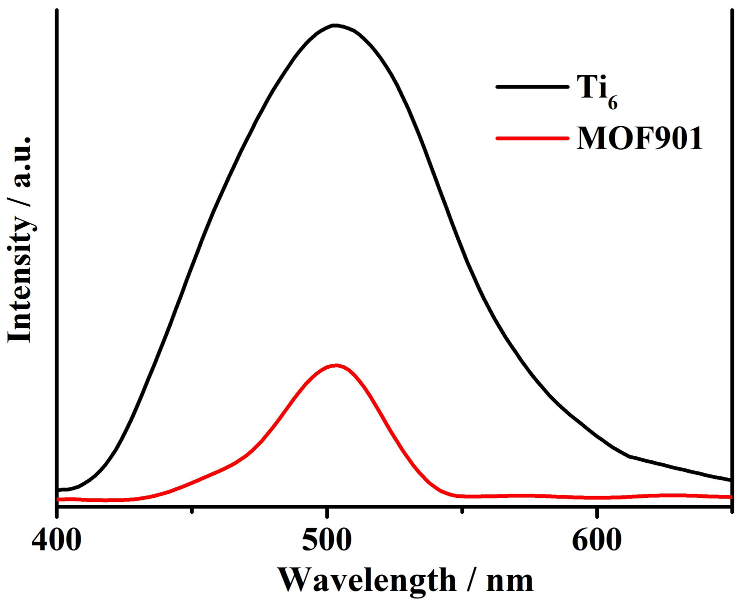

Supplementary Figure 21. Steady-state PL spectra of $\mathbf{Ti_6}$ and MOF901 by using 360 nm laser irradiation. Other test conditions are the same.

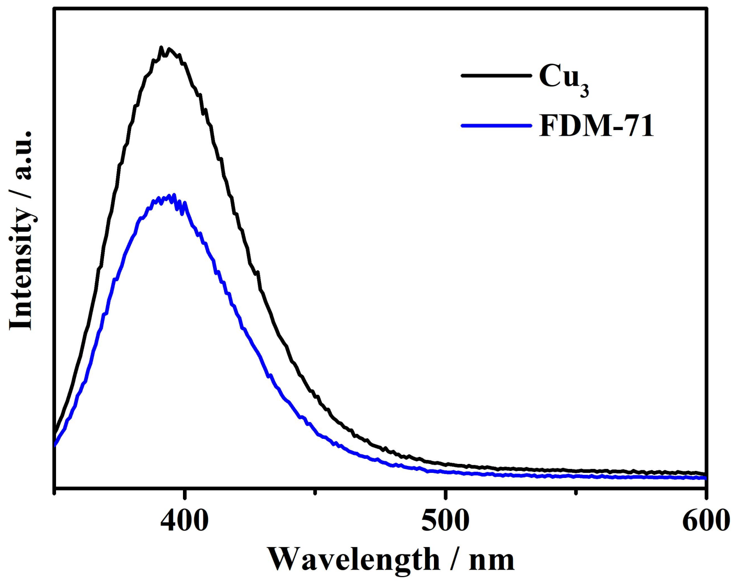

Supplementary Figure 22. Steady-state PL spectra of $\mathbf{Cu_3}$ and FDM-71-ABC by using 250 nm laser irradiation. Other test conditions are the same.

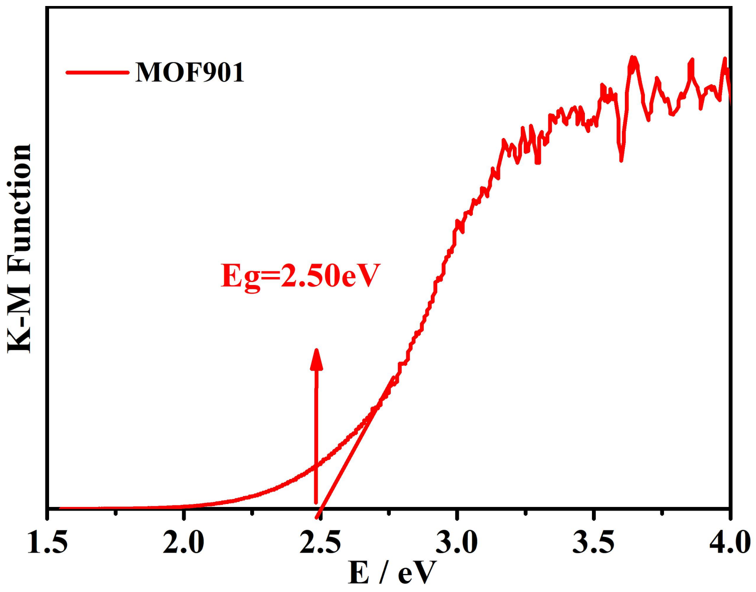

Supplementary Figure 23. Tauc plot of MOF901.

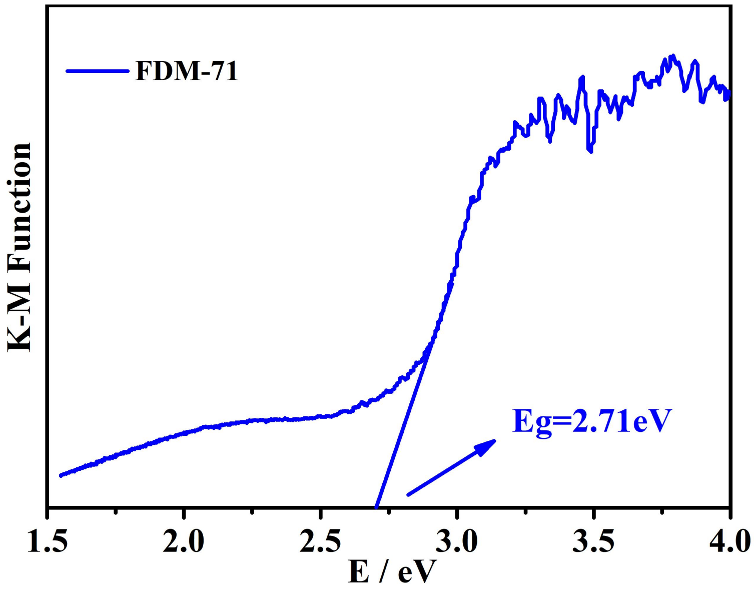

Supplementary Figure 24. Tauc plot of FDM-71-ABC.

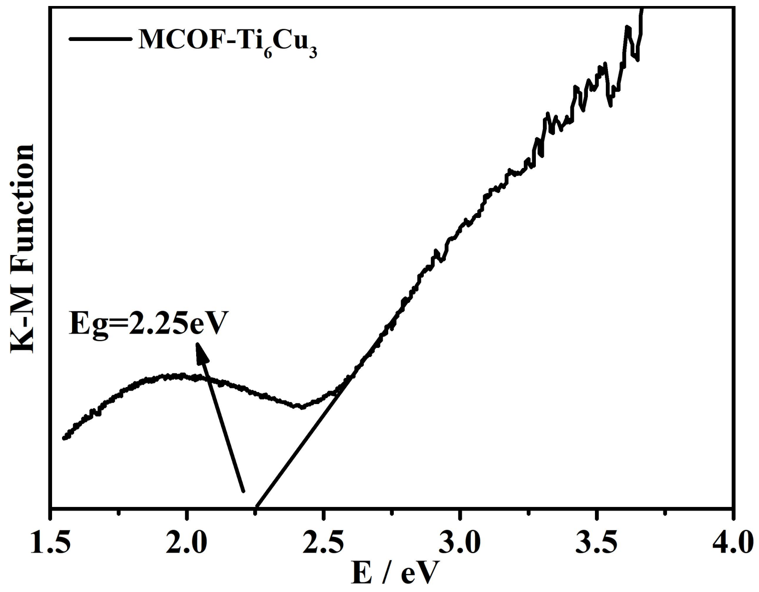

Supplementary Figure 25. Tauc plot of MCOF-Ti_6Cu_3.

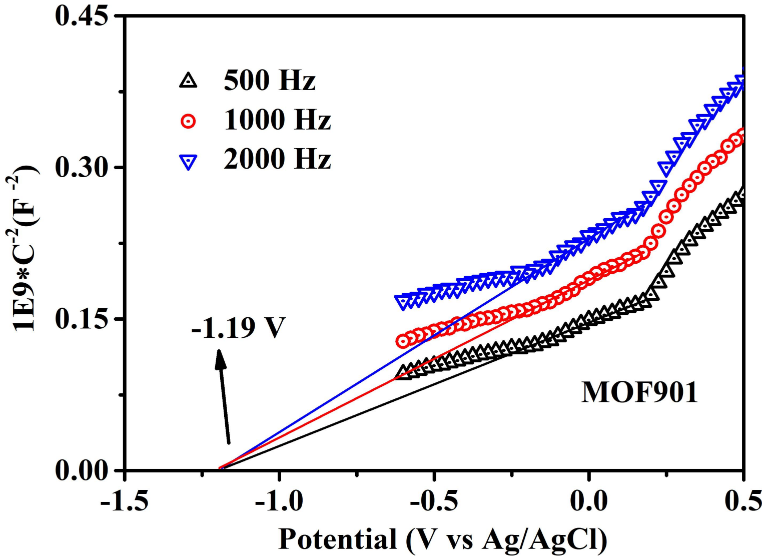

Supplementary Figure 26. Mott-Schottky plots of MOF901 in 0.1 M Na_2SO_4 aqueous solution.

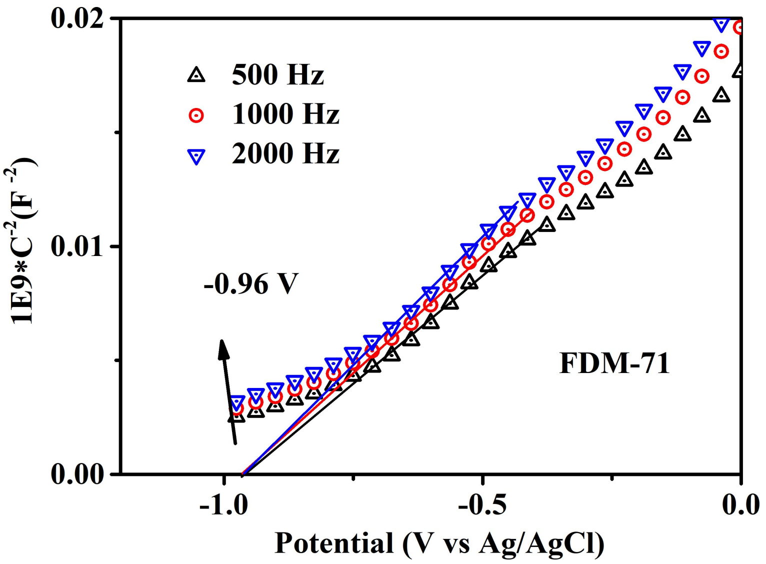

Supplementary Figure 27. Mott-Schottky plots of FDM-71-ABC in 0.1 M Na_2SO_4 aqueous solution.

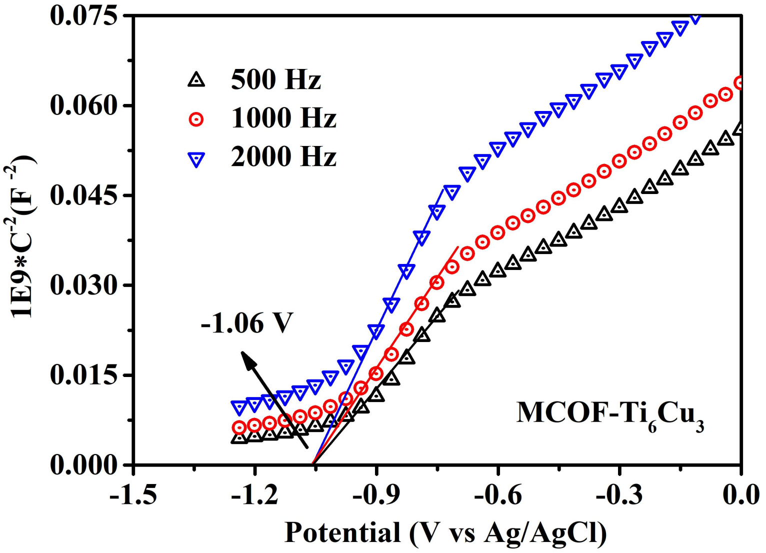

Supplementary Figure 28. Mott-Schottky plots of MCOF-Ti_6Cu_3 in 0.1 M Na_2SO_4 aqueous solution.

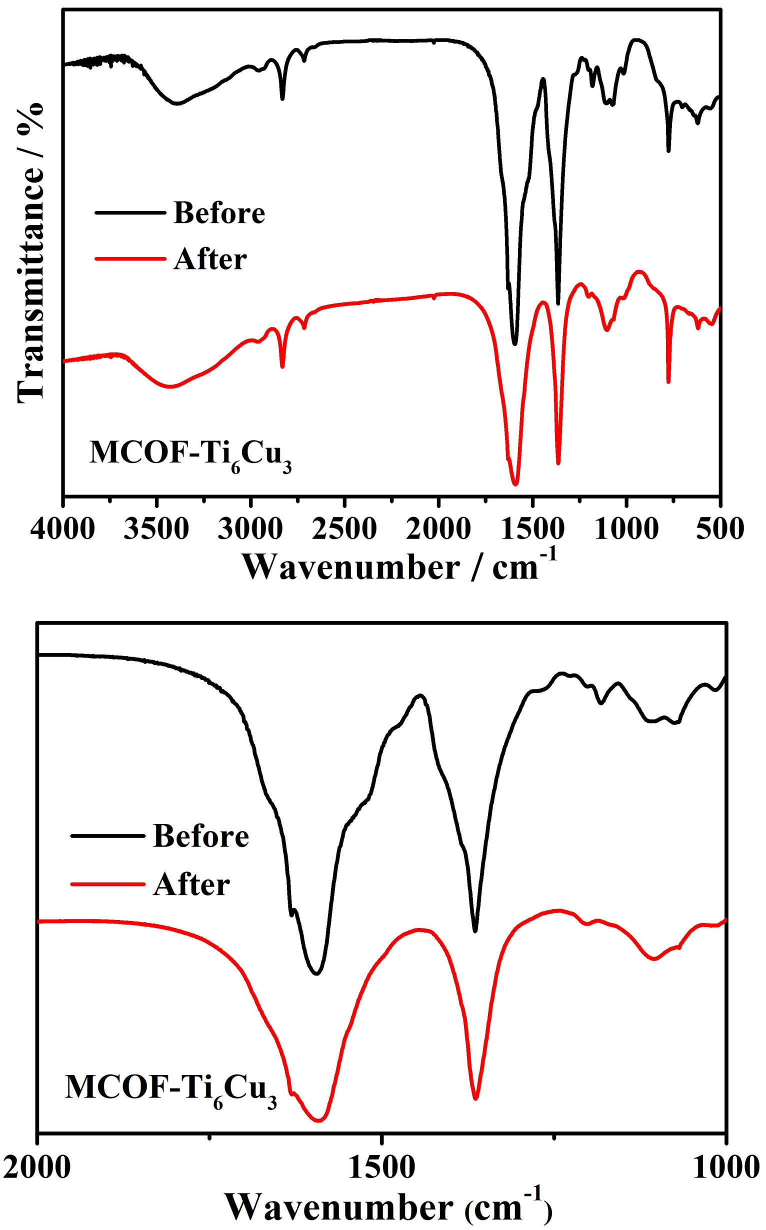

Supplementary Figure 29. The IR spectrum of MCOF-Ti_6Cu_3 powder with before and after photocatalytic experiment. The bottom is an enlarged view of the top.

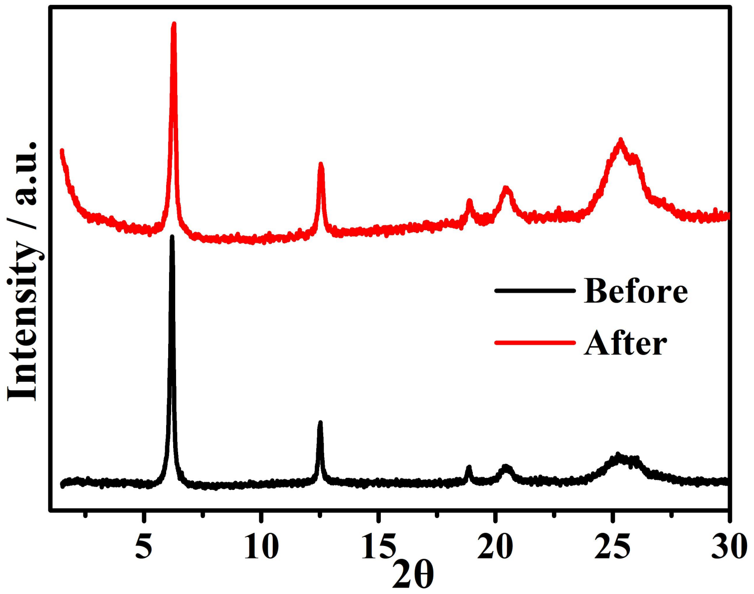

Supplementary Figure 30. The XRD pattern of MCOF-Ti_6Cu_3 powder with before and after photocatalytic experiments.

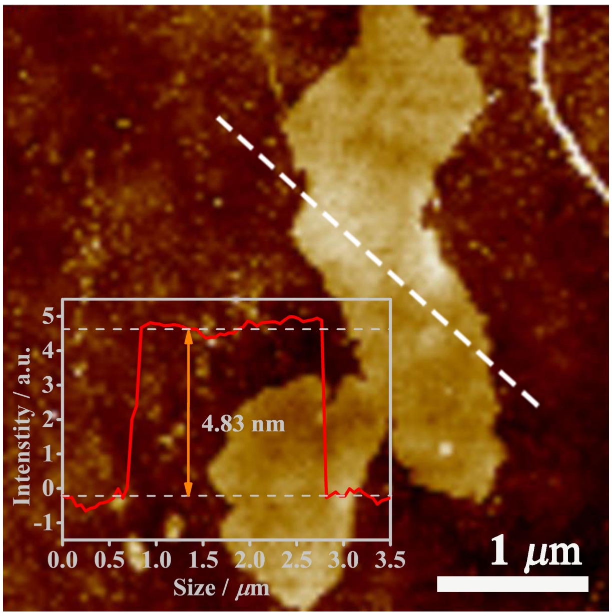

Supplementary Figure 31. AFM image of MCOF-Ti_6Cu_3 after high frequency ultrasound 30 min with H_2O.

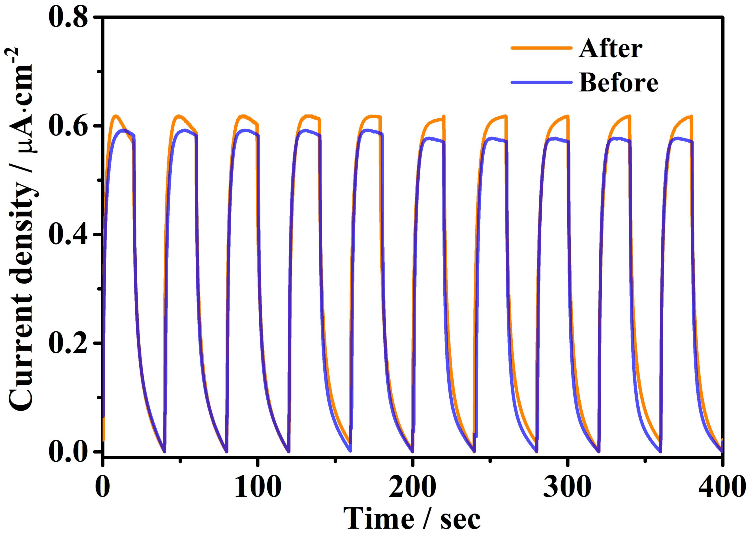

Supplementary Figure 32. Transient photocurrent responses of MCOF-Ti_6Cu_3 samples before and after stripping.

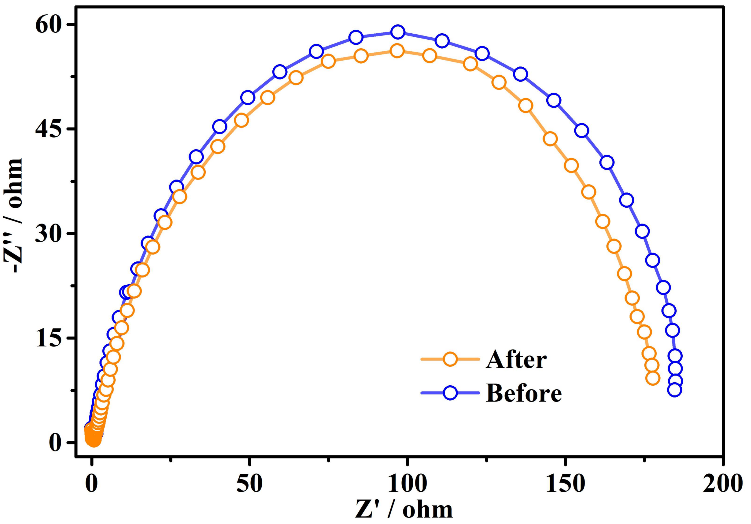

Supplementary Figure 33. Electrochemical impedance spectroscopy of MCOF-Ti_6Cu_3 samples before and after stripping.

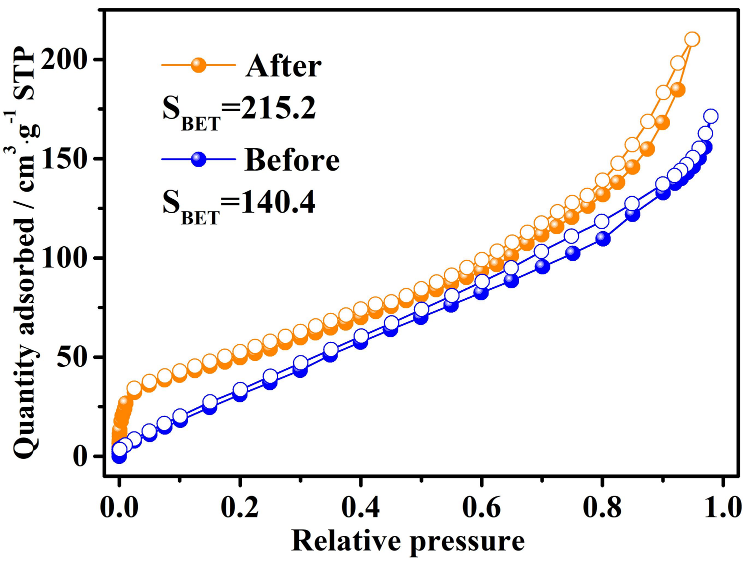

Supplementary Figure 34. N_2 adsorption isotherms of MCOF-Ti_6Cu_3 samples at 77 K before and after stripping.

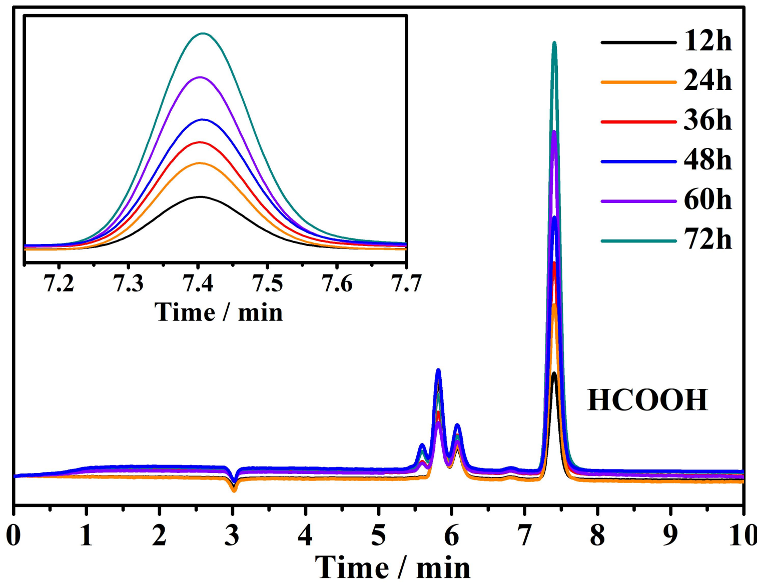

Supplementary Figure 35. The IC curvesas a function of time for MCOF-Ti_6Cu_3.

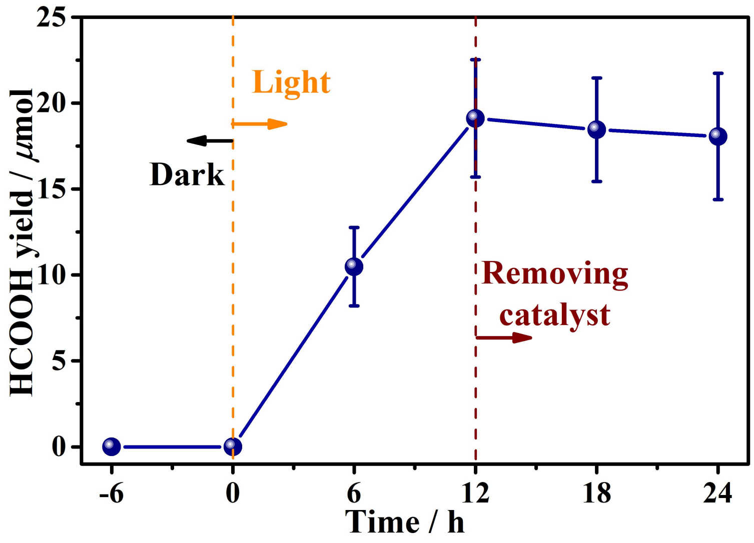

Supplementary Figure 36. Heterogeneity test (the test for removing the catalyst from the reaction system after 12 h of running the photocatalytic reaction). The yield decreased after removing the catalyst. This is probably because that a new round of 20 min CO_2 bubbling took away a small amount of product in solution.

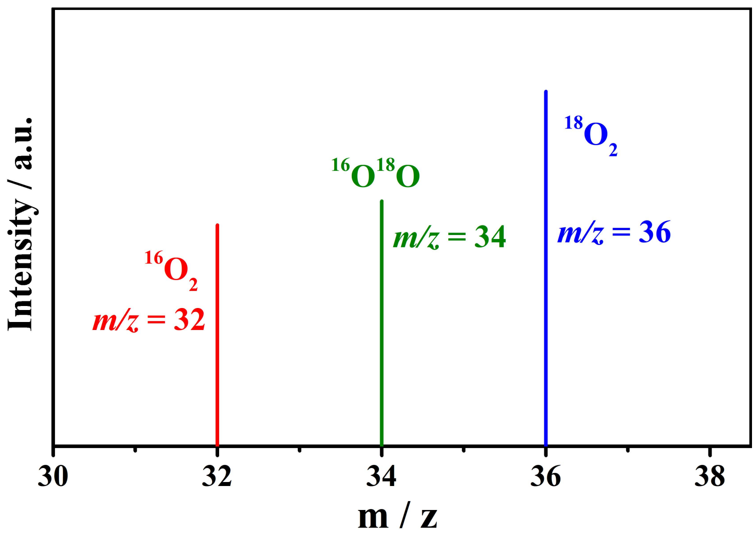

Supplementary Figure 37. GC-Mass spectra of MCOF-Ti_6Cu_3 in the photocatalytic reaction of $H_2{}^{18}O$.

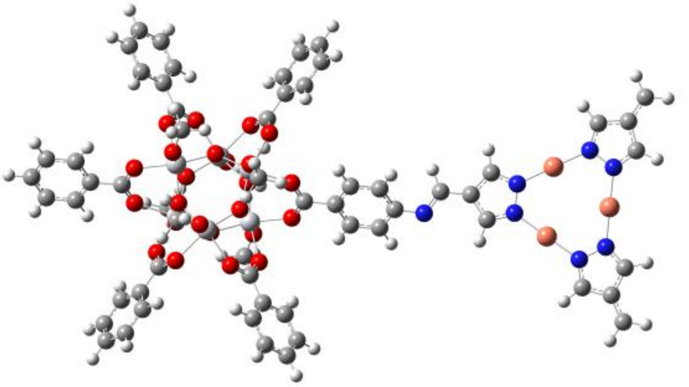

Supplementary Figure 38. Computational model image for MCOF-Ti_6Cu_3 with a cluster model.

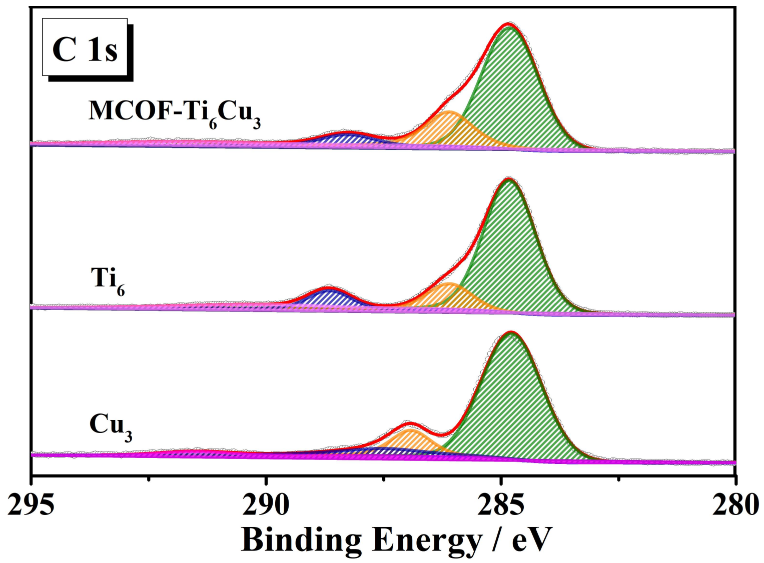

Supplementary Figure 39. X-ray photoelectron spectroscopy (XPS) of C 1*s* for **Cu_3**, **Ti_6** and MCOF-Ti_6Cu_3.

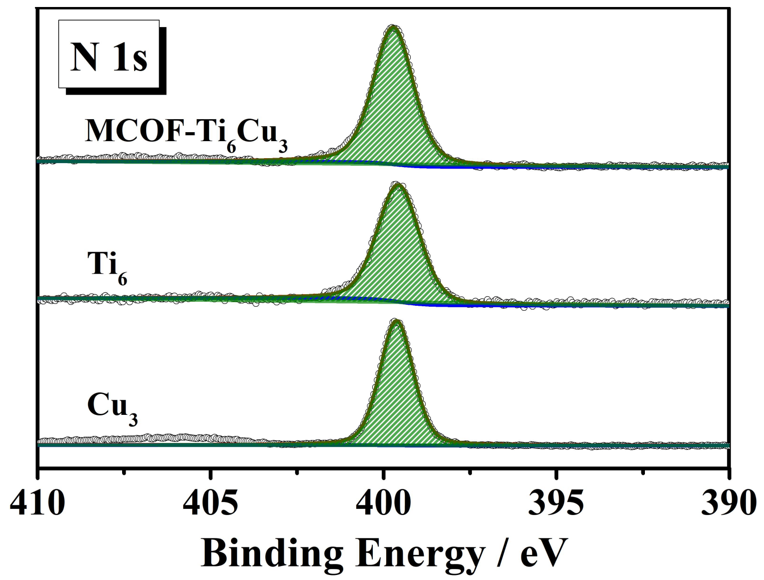

Supplementary Figure 40. X-ray photoelectron spectroscopy (XPS) of N 1*s* for **Cu_3**, **Ti_6** and MCOF-Ti_6Cu_3.

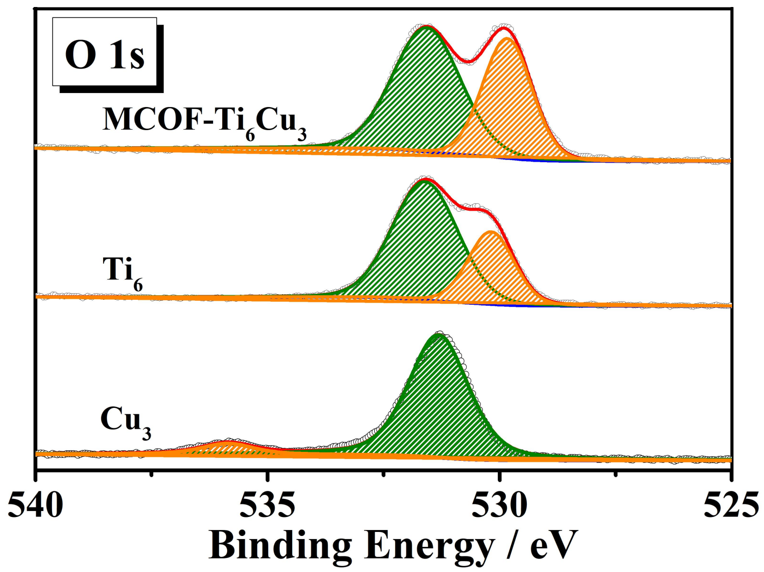

Supplementary Figure 41. X-ray photoelectron spectroscopy (XPS) of O 1*s* for **Cu_3**, **Ti_6** and MCOF-Ti_6Cu_3.

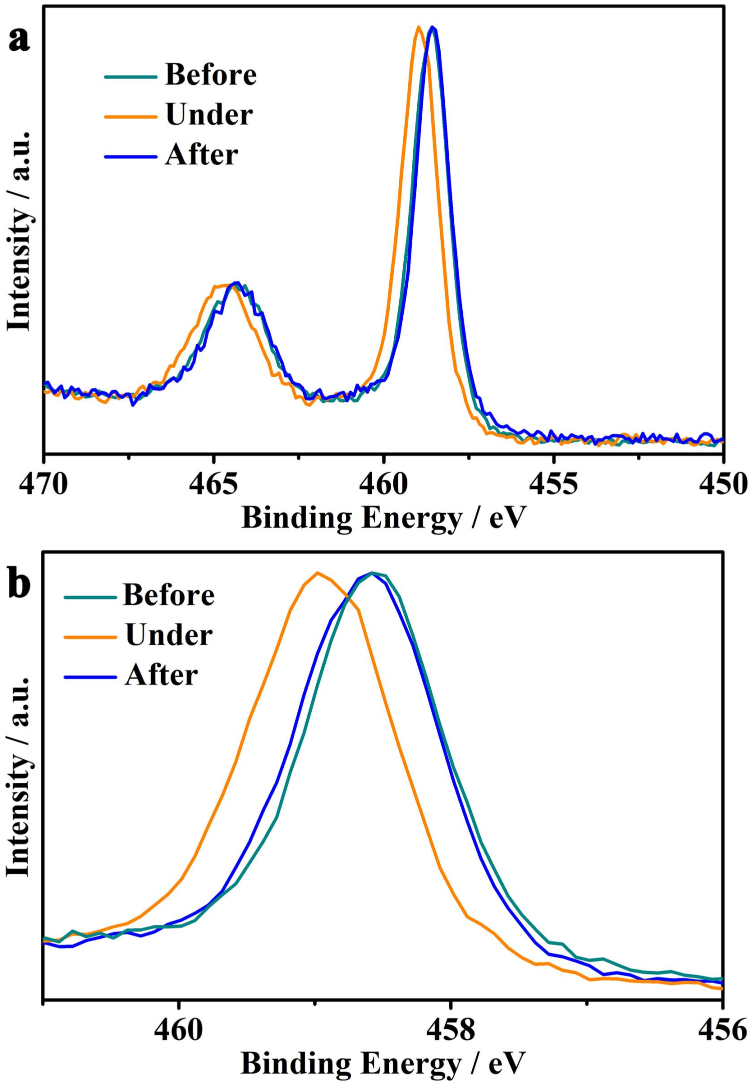

Supplementary Figure 42. *In-situ* XPS spectra. **b** is an enlarged view of **a**.

The Ti 2*p* for the simulated solar-driven CO_2 reduction process over MCOF-Ti_6Cu_3. Before: before the photocatalytic reaction started; Under: in the photocatalytic reaction; After: after the photocatalytic reaction.

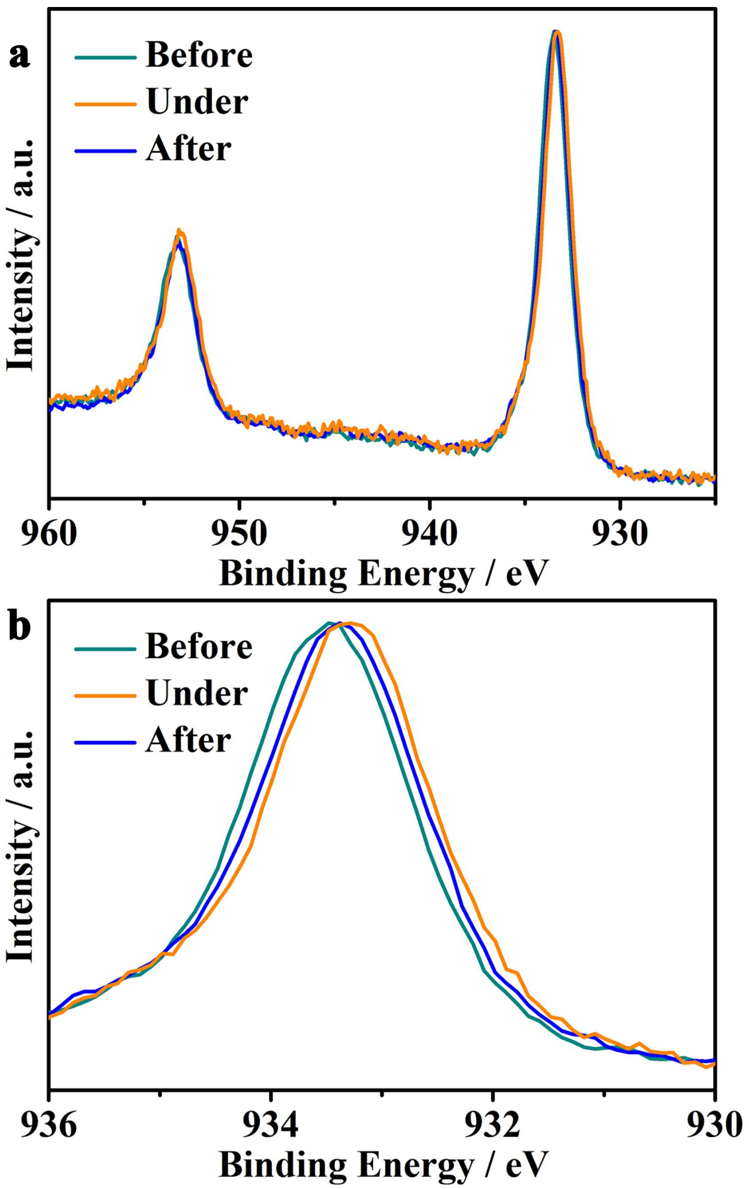

Supplementary Figure 43. *In-situ* XPS spectra. **b** is an enlarged view of **a**.

The Cu 2*p* for the simulated solar-driven CO_2 reduction process over MCOF-Ti_6Cu_3. Before: before the photocatalytic reaction started; Under: in the photocatalytic reaction; After: after the photocatalytic reaction.

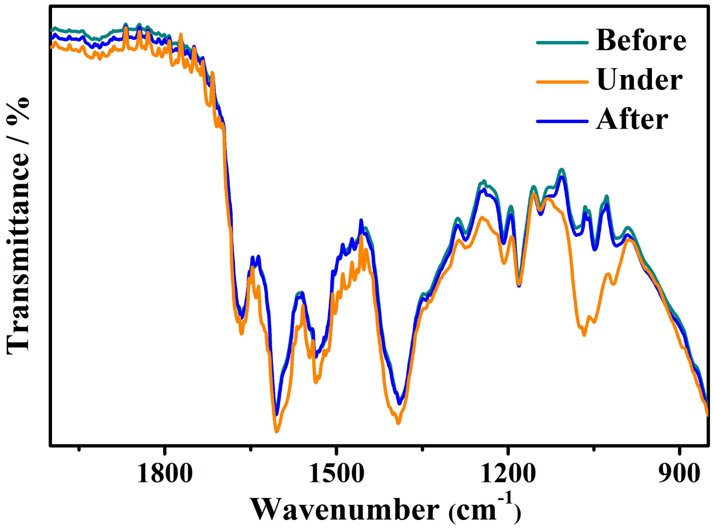

Supplementary Figure 44. *In-situ* DRIFTS spectra for the simulated solar-driven CO_2 reduction process over MCOF-Ti_6Cu_3.

Before: before the photocatalytic reaction started; Under: in the photocatalytic reaction; After: after the photocatalytic reaction.

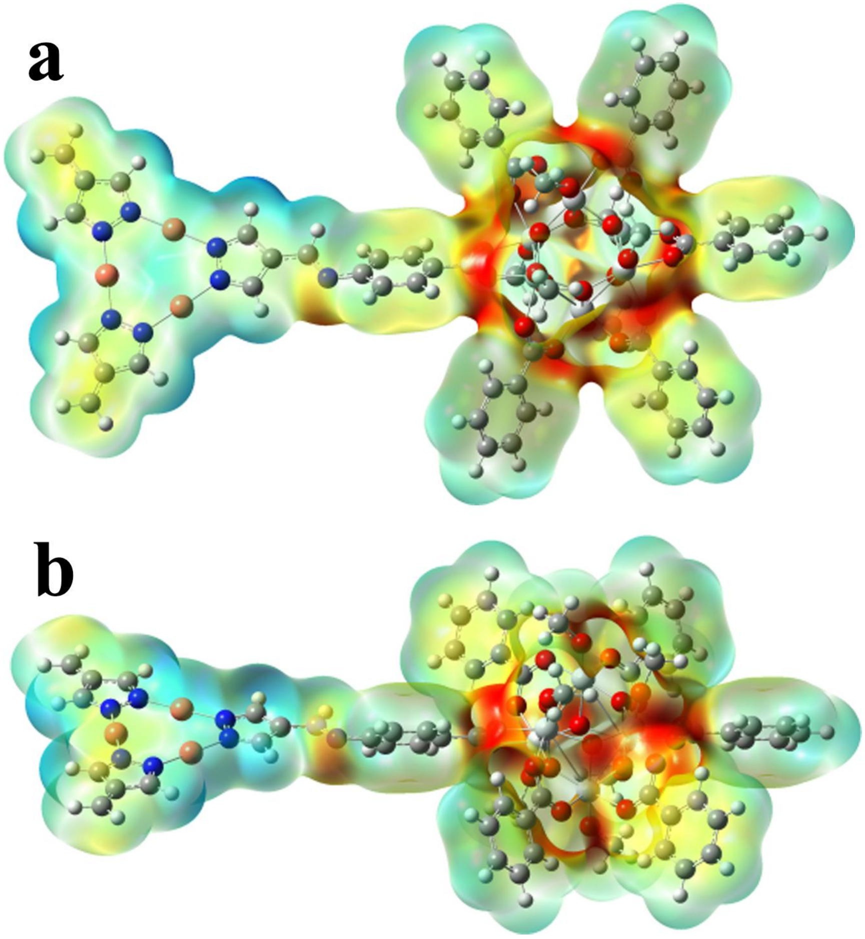

Supplementary Figure 45. Electrostatic potential pattern of MCOF-Ti_6Cu_3. **a** top view and **b** side view.

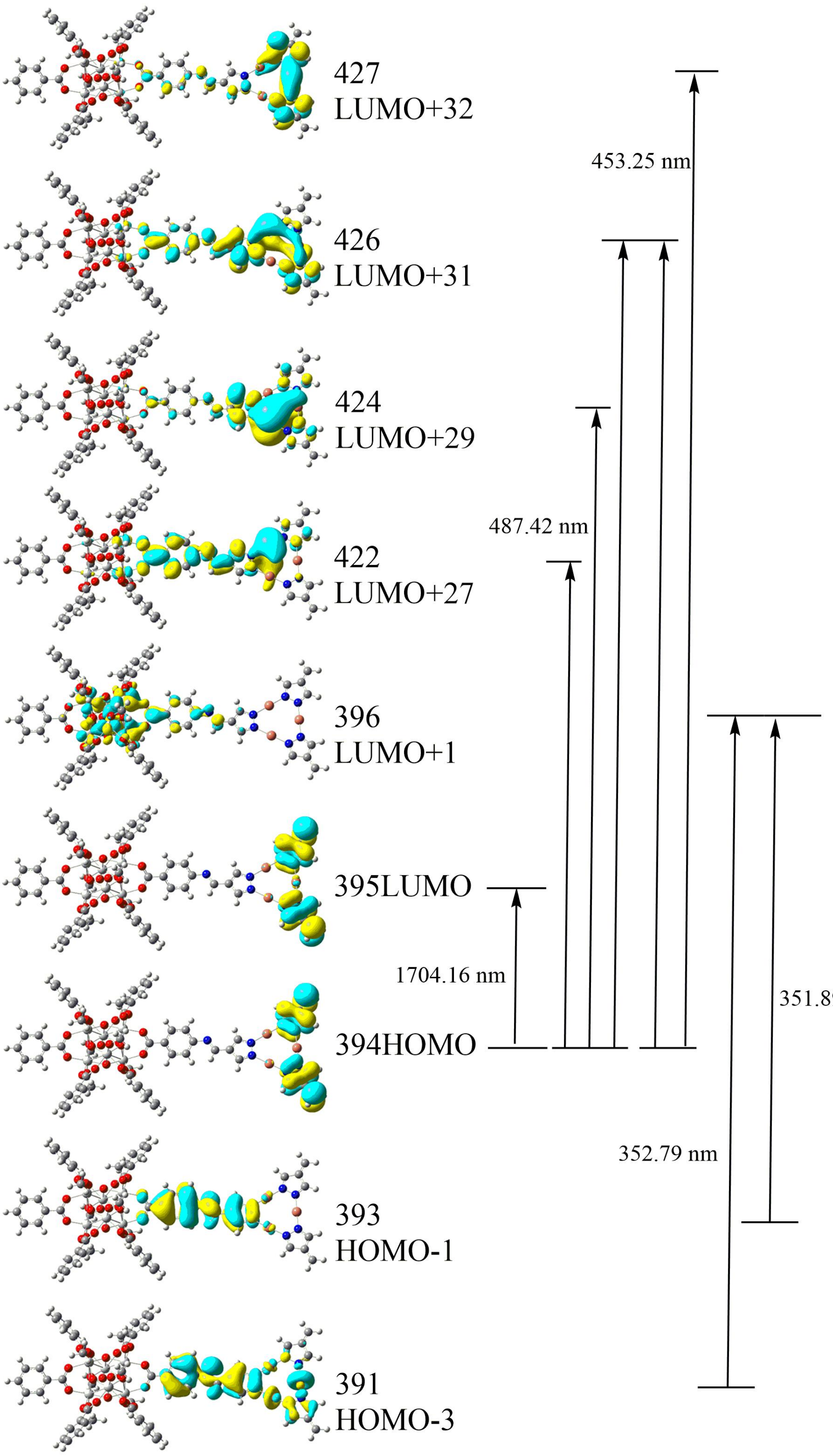

Supplementary Figure 46. The molecular orbital composition analysis of MCOF-Ti_6Cu_3.

According to the DFT calculation results (see Supplementary Figure 35), visible and ultraviolet lights correspond to multiple excitation modes: The visible light (wavelengths of 487.42 nm and 453.25 nm; ultraviolet light (352.79 nm and 351.89 nm) . The 487.42 nm excitation accounts for a larger portion, corresponding the calculated E_g value of 2.54 eV. Therefore, the experimental E_g value obtained by MCOF-Ti_6Cu_3 in Mott-Schottky measurements is 2.25 eV, which is consistent with the optical excitation mode corresponding to the calculated 487.42 nm. The main reason for the discrepancy is the simplified model selected by the Gaussian program, rather than an ordered infinite plane structure. Another reason is that the computational model is gaseous and the experimental material is solid state.

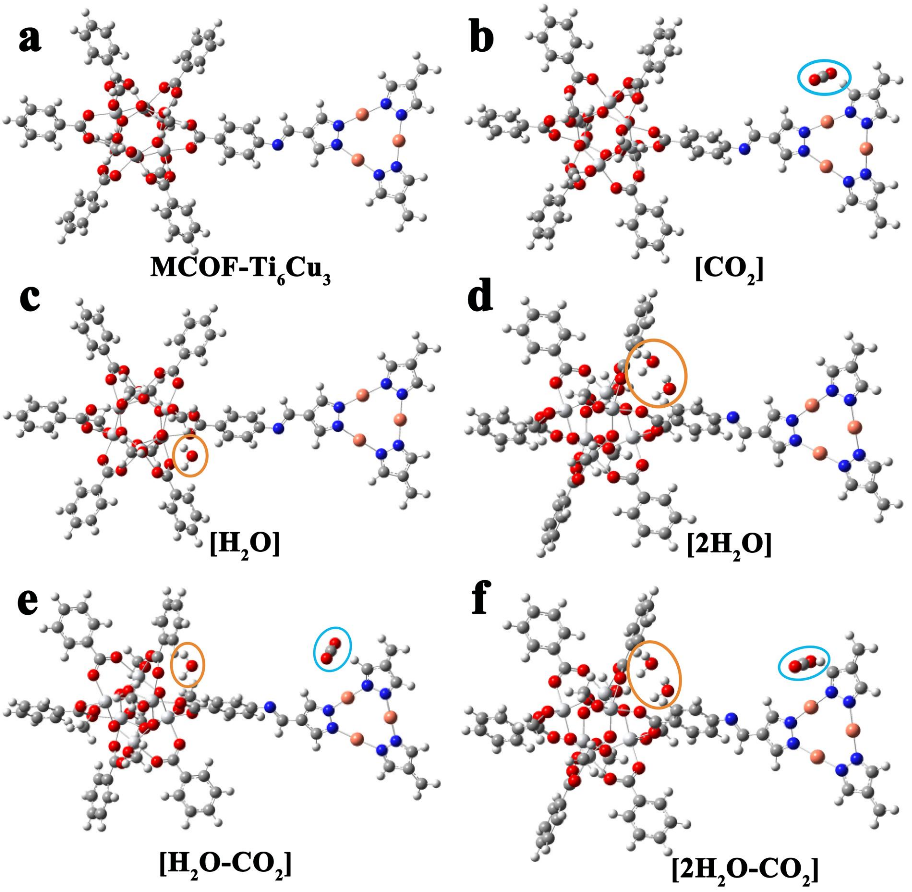

Supplementary Figure 47. The model plots of CO_2 and H_2O at adsorption sites on MCOF-Ti_6Cu_3. **a** MCOF-Ti_6Cu_3, **b** [CO_2], **c** [H_2O], **d** [2H_2O], **e** [H_2O-CO_2], **f** [2H_2O-CO_2].

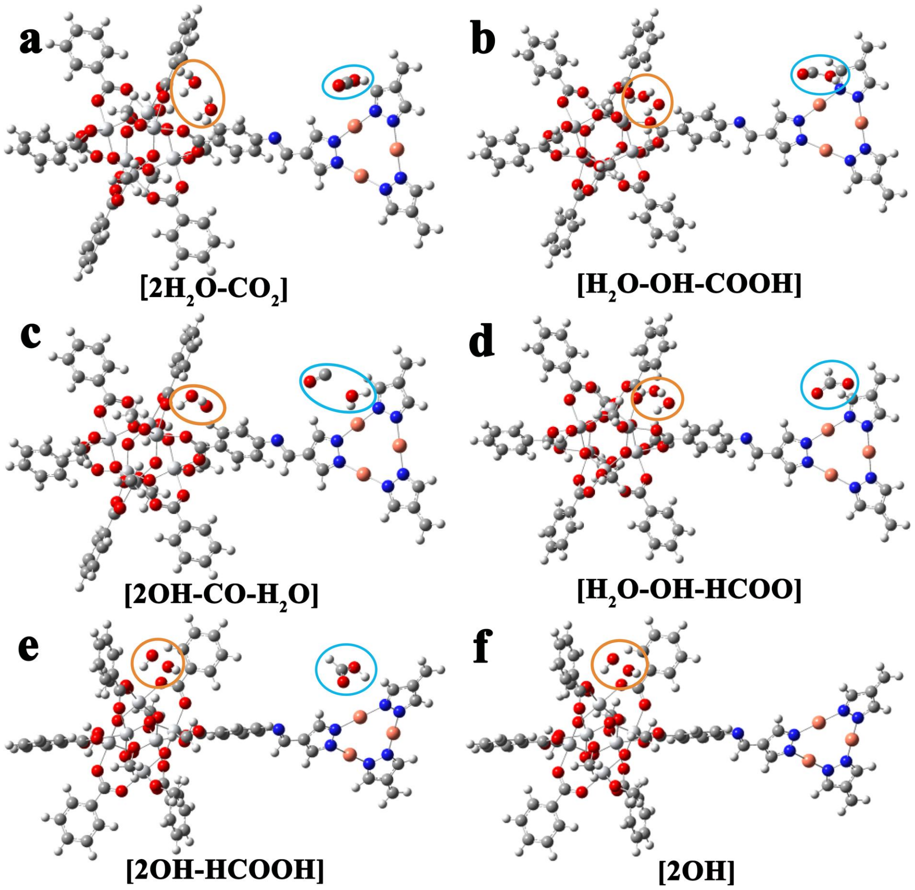

Supplementary Figure 48. The model plots of partial reaction processes on MCOF-Ti_6Cu_3. **a** [$2H_2O$-CO_2], **b** [H_2O-OH-COOH], **c** [2OH-CO-H_2O], **d** [H_2O-OH-HCOO], **e** [2OH-HCOOH], **f** [2OH].

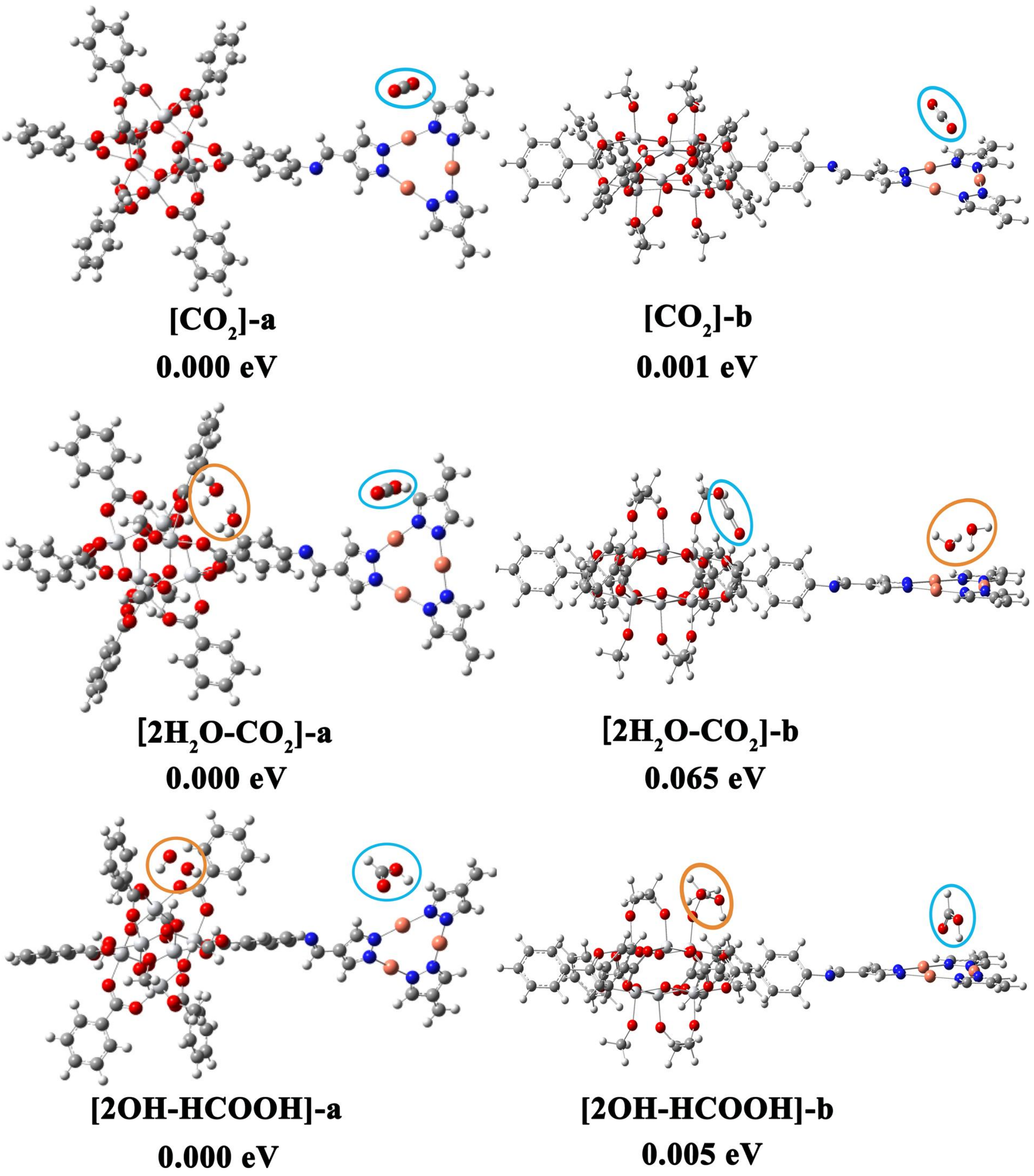

Supplementary Figure 49. The model plots of some adsorption sites on MCOF-Ti_6Cu_3. **a** [CO_2]-a, **b** [CO_2]-b, **c** [$2H_2O$-CO_2]-a, **d** [$2H_2O$-CO_2]-b, **e** [2OH-HCOOH]-a, **f** [2OH-HCOOH]-b.

The electron of [CO_2]-b, [$2H_2O$-CO_2]-b and [2OH-HCOOH]-b are relative to [CO_2]-a, [$2H_2O$-CO_2]-a and [2OH-HCOOH]-a, respectively. The corresponding energy is almost equal, confirming that the selected models are reasonable.

Supplementary Tables

Supplementary Table 1. Fractional atomic coordinates for the structure of ABC stacking calculated with the Materials Studio v19.1 modeling program.

R_3 (146) Trigonal			
$a = b = 29.1420$ Å, $c = 18.9759$ Å			
$\alpha = \beta = 90°$ and $\gamma = 120°$			
Atom	x/a	y/b	z/c
C1	0.0987	0.22303	0.96718
C2	0.14346	0.25286	0.92518
C3	0.17348	0.30774	0.93322
C4	0.15876	0.33355	0.98256
C5	0.11348	0.30386	1.02404
C6	0.0838	0.24883	1.01659
N7	0.77953	0.17716	0.90636
C8	0.17547	0.41916	1.02024
C9	0.89264	0.76687	0.95083
C10	0.84557	0.73317	0.98671
C11	0.81844	0.6787	0.9724
C12	0.8386	0.65762	0.92268
C13	0.88615	0.69117	0.88853
C14	0.91266	0.74541	0.9019
N15	0.19791	0.79772	0.99137
C16	0.7615	0.5714	0.91219
O17	0.57532	0.3215	0.1705
O18	0.59934	0.41135	0.35512
Ti19	0.58709	0.32219	0.26557
C20	0.56261	0.40065	0.31304
O21	0.65334	0.38339	0.26718
O22	0.5603	0.3658	0.27357
Ti23	0.65224	0.39538	0.36537
O24	0.64915	0.4054	0.46047
O25	0.52286	0.22963	0.35665
C26	0.49139	0.2327	0.31577
O27	0.51866	0.26969	0.27507
O28	0.59977	0.32387	0.36405
N29	0.76623	0.39104	0.713
C30	0.81827	0.42558	0.70683
Cu31	0.68743	0.28025	0.71386
N32	0.75802	0.34224	0.71503
C33	0.8045	0.34313	0.71142
C34	0.84323	0.39581	0.70625
N35	0.67939	0.43098	0.8843

C36	0.68397	0.47882	0.89352
Cu37	0.73758	0.37496	0.88387
N38	0.72682	0.43567	0.88287
C39	0.76415	0.48677	0.89026
C40	0.73761	0.51485	0.89673
C41	0.36358	0.76865	0.82192
C42	0.37273	0.73779	0.87866
C43	0.35573	0.81135	0.8553
C44	0.38465	0.60421	0.45686
C45	0.38941	0.55961	0.4237
C46	0.38563	0.64116	0.40042

Supplementary Table 2. Fractional atomic coordinates for the of the structure of AB stacking calculated with the Materials Studio v19.1 modeling program.

$P6_3$ (173) hexagonal			
$a = b = 28.8837$ Å, $c = 16.0155$ Å			
$\alpha = \beta = 90°$ and $\gamma = 120°$			
Atom	x/a	y/b	z/c
O1	0.74533	0.41028	0.07135
C2	0.79063	0.14591	0.19907
C3	0.97871	0.51864	0.20905
N4	1.0586	0.5218	0.26175
O5	0.80663	0.38037	0.29336
C6	0.7518	0.41562	-0.00739
O7	0.66573	0.27595	0.3008
Ti8	0.73749	0.40334	0.18515
C9	0.83698	0.41667	0.24516
C10	0.97421	0.44849	0.29736
O11	0.72331	0.3327	0.18758
O12	0.80817	0.42433	0.19572
Ti13	0.7361	0.33216	0.30341
C14	0.89247	0.44335	0.24728
C15	0.92285	0.49147	0.2039
C16	1.00469	0.49706	0.2558
C17	0.75028	0.33408	0.49586
C18	0.77913	0.21975	0.24434
C19	0.83868	0.16695	0.2444
C20	0.76145	0.17266	0.19811
N21	0.86847	0.14273	0.24587
O22	0.71441	0.24012	0.19515
C23	0.9185	0.42223	0.29427
O24	0.75697	0.28274	0.29361
O25	0.74377	0.33299	0.41717
C26	0.75029	0.24642	0.24481
C27	0.82667	0.24025	0.2902
C28	0.85661	0.21445	0.28912
N29	0.1006	0.08503	0.21157
C30	0.14832	0.13034	0.21054
Cu31	0.04837	-0.03017	0.21406
N32	0.10752	0.04325	0.2164
C33	0.15995	0.05968	0.22131
C34	0.18646	0.11498	0.21713
C35	0.24121	0.14932	0.22071
N36	0.77407	0.39804	-0.25596
C37	0.82647	0.43433	-0.25388

Cu38	0.69719	0.2853	-0.25815
N39	0.7674	0.34942	-0.26029
C40	0.81522	0.35215	-0.26368
C41	0.85318	0.40579	-0.25917
C42	0.90803	0.42671	-0.25986
C43	0.23942	0.62097	1.04041
C44	0.46055	0.75392	1.03458
C45	0.62063	0.38005	-0.05252
C46	0.5393	0.29638	-0.04599

Supplementary Table 3 The analysis of Ti-to-Cu ratio of MCOF-Ti_6Cu_3.

Method	Ti / %	Cu / %	Ti / Cu
Sim	12.84	17.05	0.753
ICP	13.28	17.11	0.776
EDS	10.17	11.15	0.912
XPS	11.42	13.79	0.828

The Ti/Cu ratio in MCOF-Ti_6Cu_3 measured by ICP (0.776) is consistent with the theoretical one (0.753), confirming the chemical composition of the structural model. However, the Ti/Cu ratios obtained by EDS and XPS show slight discrepancy. This is probably due to the sample being not homogenous during the EDS/XPD tests.

Supplementary Table 4. Fitted lifetimes of the fluorescence decay profiles from Fig. 2d.

	A_1%	T_1 / ns	A_2%	T_2 / ns	τ / ns
MCOF-Ti_6Cu_3	18.87	142.74	81.13	2303.09	1895.33±257.97
MOF901	21.75	118.67	78.25	1597.32	1275.66±145.61
FDM-71-ABC	22.59	133.52	77.41	2116.28	1668.45±89.24

Supplementary Table 5 Summary of photocatalysts toward CO_2 Reduction to HCOOH.

Photocatalyst	Solvent	Sacrificial electron donor	Light source	Products (μmol·g^{-1}·h^{-1})	Ref
Eu-Ru(phen)$_3$-MOF	MeCN	TEOA	300W Xe lamp (420 < λ ≤ 800 nm)	94	6
COF-367-Co	MeCN	TEA	300W Xe lamp (380 < λ ≤ 800 nm)	97.63	7
Eubpy-Ru-40%-CuCl/pyridine	MeCN/ H_2O	TIPA	300W Xe lamp (400 < λ ≤ 800 nm)	304.0	8
PCN-222	MeCN	TEOA	300W Xe lamp (420 < λ ≤ 800nm)	60	9
NNU-28	MeCN	TEOA	300W Xe lamp (420 < λ ≤ 800nm)	52.8	10
NH_2-C@Cu_2O	H_2O	Na_2SO_3	300W Xe lamp AM 1.5G	138.65	11
AD-MOF-2	H_2O	TIPA	300W Xe lamp (420 < λ ≤ 800nm)	443.2	12
SiC	NaOH solution	No	300W Xe lamp (420 < λ ≤ 800nm)	12	13
TiO_2/Cu(II) phthalocyanine	H_2O	No	300W Xe lamp (420 < λ ≤ 800nm)	26.05	14
NNU-31-Zn	H_2O	No	300W Xe lamp (420 < λ ≤ 800 nm)	26.3	15
Cu_2O-Pt/SiC/IrO_x	H_2O	No	300W Xe lamp 420 nm < λ	896.6	16
MCOF-Ti_6Cu_3	H_2O	No	300W Xe lamp AM 1.5G	169.8	This work

Supplementary Table 6. The free energy of CO_2 and H_2O on MCOF-Ti_6Cu_3 with different model.

Model	Ground State Δ*E* (eV)	Excited State Δ*E* (eV)
[MCOF-Ti_6Cu_3]	0.000	0.000
[MCOF-Ti_6Cu_3]-[CO_2]	0.151	-0.012
[MCOF-Ti_6Cu_3]-[H_2O]	-0.050	-0.420
[MCOF-Ti_6Cu_3]-[$2H_2O$]	-0.308	-0.964
[MCOF-Ti_6Cu_3]-[H_2O-CO_2]	0.097	-0.436
[MCOF-Ti_6Cu_3]-[$2H_2O$-CO_2]	-0.076	-0.980

Supplementary Table 7. The free energy of partial reaction processes on MCOF-Ti_6Cu_3.

Model	Ground State ΔE (eV)	Excited State ΔE (eV)
[MCOF-Ti_6Cu_3]	0.000	0.000
[MCOF-Ti_6Cu_3]-[$2H_2O$-CO_2]	-0.076	-0.980
[MCOF-Ti_6Cu_3]-[H_2O-OH-COOH]	3.451	2.547
[MCOF-Ti_6Cu_3]-[2OH-CO-H_2O]	4.178	3.274
[MCOF-Ti_6Cu_3]-[H_2O-OH-HCOO]	3.884	2.980
[MCOF-Ti_6Cu_3]-[2OH-HCOOH]	3.288	2.383
[MCOF-Ti_6Cu_3]-[2OH]	3.530	2.625

Supplementary Notes

Supplementary Note 1: Materials and General Methods.

The powder X-ray diffraction (PXRD) spectra were recorded on a D/max 2500 VL/PC X-ray diffractometer (Rigaku SmartLab, Japan) with Cu Kα radiation (λ = 1.5418 Å) at 45 kV, 200 mA. Diffraction intensity data for 2θ from 1.5 ~ 30° were collected at the scanning speed of 5 deg/min with 2θ step increment of 0.01°. Fourier-transform infrared (FTIR) and *in-situ* FT-IR spectra were obtained from KBr pellets in a wavelength ranging from 4000-500 cm^{-1} on a Thermo Nicolet IS50 FTIR spectrometer. X-ray photoelectron spectroscopy (XPS) were recorded using a Escalab 250Xi instrument from Thermo Scientific equipped with an Al Kα microfocused X-ray source and the C 1s peak at 284.6 eV as internal standard. The *in-situ* XPS were recorded using United States-Thermo Fisher SCIENTIFIC ESCALAB 250Xi. Nitrogen gas porosimetry measurements were performed on a automatic volumetric adsorption equipment using a (ASIQM0G002-3) and porosity analyzer after the samples were outgassed under a vacuum at 120 ℃ for 12 h. Apparent surface areas were determined using BET method. Pore size distributions of COFs were caculated using nonlocal density functional theory (NL-DFT) model. Emission lifetime measurements were performed on photon technology international quanta master/time master TM 400 phosphorescence/fluorescence Spectrofluoromete. TEM images were recorded on a JEM-2010 transmission electron microscope at an accelerating voltage of 200 kV. SEM and EDS images were recorded on XL-30 ESEM-FEG Scanning Electron Microscope. Thermogravimetric analysis of COFs powder samples was performed on a Diamond TG/DTA/DSC Thermal Analyzer System (Perkin-Elmer, USA) with heating rate of 10 ℃ min^{-1} from room temperature to 1000 ℃ under air atmosphere. The UV-vis absorption spectra of COFs were recorded on a Shimadzu UV-2450 spectrophotometer. The pH was carried out with a pH meter which was calibrated with buffer reagent of mixed phosphate (6.86, INESA Scientific Instrument Co.). The gas product in photocatalytic reaction was measured by Agilent Gas Chromatography. The liquid products in liquid phase of the reactor were measured using IC (Dionex Aquion RFIC, Thermo Scientific. America) to analyze HCOOH.

Supplementary Note 2: Photo-product detection

The gaseous products were sampled by an off-line sampling syringe (0.5 mL) and then analysed by gas chromatography. To detect the yield of CO gas, gas (500 μL) in the middle of the test tube was collected using an off-line sampling syringe and injected into a gas chromatography system equipped with an FID detector using argon as the carrier gas. Retention time of CO was 1.9 min. To detect the formation of H_2 and O_2, the gas (500 μL) of the test tube was transferred using an off-line sampling syringe and analysed by gas chromatography with TCD detector through a 5 Å molecular sieve column, with ultra-pure argon acting as both carrier and reference gas. Retention time of H_2 and O_2 were 1.9 min and 4.3 min. The volumes of the CO, H_2 or O_2 produced were calculated by comparing the integrated areas of the signals of CO, H_2 or O_2 with a calibration curve. The injector and detector temperatures were kept to be 60 °C.

The liquid products in the liquid phase of the reactor were measured using ion chromatography (IC, Dionex Aquion RFIC, Thermo Scientific. America) to analyze $HCOO^-$. The flow velocity of the IC mobile phase was 1 mL/min, and the sampling volume of the automatic injection syringe was 25 μL (the injection needle needs to be washed three times with deionized water between different standards, and the needle is rinsed in water before it withdraws samples). The solution obtained after photocatalysis was filtered with a filter head (0.22 μm) and a syringe was used to obtain a clear solution of the filtrate. 1 ml of the filtrate is collected and diluted 100 times with a 100 ml volumetric flask. By comparing the peak area of the liquid-phase product with the standard curve, the volume of the liquid-phase product $HCOO^-$ can be calculated.

Supplementary Note 3: Isotopic labeling control experiments

^{13}C : In a quartz reactor (50 mL), catalyst (100 mg) was dispersed in ultra-pure water (10 mL). After sonication for about 15 minutes, the quartz tube was then flash frozen at 77 K (liquid N_2 bath) and degassed by three freeze-pump-thaw cycles, eventually filled with $^{13}CO_2$ (standard atmospheric pressure).

^{18}O : In a quartz reactor (10 mL), catalyst (10 mg) was dispersed in $H_2{}^{18}O$ (1 mL, the purity of $H_2{}^{18}O$ is 97 %, purchased from J&K Scientific). This mixture system was bubbled with pure CO_2 gas for 10 min.

The temperature of the reaction solution was maintained at 25 °C controlled by a circulating water bath during the reaction. Then, the system is irradiated under simulated sunlight using a PLS-SXE300 Xe lamp with an AM 1.5 cut-off filter.

Supplementary Note 4: Single layer sample preparation

In a 50 ml centrifuge tube, 30 mg of MCOF-Ti_6Cu_3 was dispersed in the 30 mL of DMF. 30 min high frequency ultrasound was carried out by ultrasonic cell pulverizer (VOSHIN-1000W, Wuxi Voshin Instruments Manufacturing Co.,LTD.). The ultrasonic power was set to 450W, the random horn was Φ6, and the working temperature was 0 °C controlled by an ice water bath. After ultrasound, the sample was separated by filtration and washed with THF by using a Soxhlet extractor for 24 hours to remove DMF. Finally, the product was evacuated at 120 °C under vacuum overnight to yield activated sample.

Supplementary Note 5: Band gap calculation

The E_g of samples were obtained from the UV-vis spectra with Tauc plot.

$$(\alpha hv)^{\frac{1}{2}} = A(hv - E_g)$$

Here, α is absorption coefficient, h is Planck constant, v is frequency, A is constant, E_g is band gap. Further processing of the data by Kubelka-Munk theory and Einstein photochemical equivalence law.

$$F(R) = (1-R)^2/2R$$

$$hv = hc / \lambda$$

Among these parameters, R is the absorbance, which can be obtained from the UV-vis diffuse reflectance spectra. λ is the wavelength of light, c is the speed of light. The curve is obtained by taking *hv* (eV) as the X axis and F(R) as the Y axis. E_g is obtained by the X axis value of intersection of the tangent of the F(R)-*E* curve and the line with y = 0 (As shown in Supplementary Figs. 23 - 25).

Supplementary Note 6: Computational Methods

All the elements of the model are as follows: Carbon (grey), Nitrogen (blue), Oxygen (red), Copper (orange), Titanium (silver) and Hydrogen (white). The calculation model is constructed by a finite cluster structure, and all calculations were performed by the Gaussian 16 software package. The ground states of MCOF-Ti_6Cu_3 were geometrically optimized by using B3LYP method of DFT for the level with a Ti, Cu/LanL2dz, other elements/6-31G* basis sets. Electrostatic potential (ESP) analysis, time-dependent DFT (TDDFT) calculations, and orbital composition were also performed at the same level. In order to obtain their corresponding excited state energies, TDDFT calculations were performed on the basis of optimized ground state configurations.

We adopt finite cluster calculations for the MCOF-Ti_6Cu_3 structure to reduce the computation time effectively, and the long range interaction for each local center is neglected. Under the condition of a fixed COF ring, the structure was optimized with the H, H_2O, and CO_2 added to the skeleton in a solvent-free state to find the best binding position of ground states. This was achieved including [MCOF-Ti_6Cu_3- CO_2], [MCOF-Ti_6Cu_3-H_2O], [MCOF-Ti_6Cu_3-2H_2O], [MCOF-Ti_6Cu_3-H_2O-CO_2], [MCOF-Ti_6Cu_3-2H_2O-CO_2], [MCOF-Ti_6Cu_3-H_2O-OH-COOH], [MCOF-Ti_6Cu_3-OH-OH-CO-H_2O], [MCOF-Ti_6Cu_3-H_2O-OH-HCOO], [MCOF-Ti_6Cu_3-OH- OH-HCOOH], [MCOF-Ti_6Cu_3-OH-OH] (MCOF-Ti_6Cu_3 will no longer be written in the latter part of the article, only highlighting the different). The vibrational frequency calculations are performed at the same level, and the obtained configurations are all positive values indicating that they could exist stably[17-19].

Supplementary Note 7: Cartesian Coordinates of Optimized Structures

MCOF-Ti_6Cu_3

Atom	x	y	z
N	-13.9902646	-2.5877663	0.3003173
C	-14.1823996	-3.8979123	0.4616493
Cu	-15.3398796	-0.1128563	-0.0407247
N	-15.2286396	-1.9775053	0.1971073
C	-16.1691896	-2.9232413	0.2967123
C	-15.5784366	-4.2129503	0.4720013
C	-16.1875986	-5.4516123	0.6155233
O	3.6879824	-2.0524283	-3.0366757
C	-4.4970176	0.3016127	-0.7959937
C	0.5264154	-7.3673463	-1.1666937
O	1.2324584	-3.0863893	0.8657903
C	4.6679314	-2.0685453	-4.0476027
O	1.9732434	0.7153927	1.0134563
Ti	3.5429294	-1.9817193	-1.2631647
C	1.5264264	-3.8800063	-0.0765647
C	-0.7560016	-6.7726133	0.8001443
O	2.0545114	-0.9157933	-0.9581987
O	2.3695034	-3.6181323	-0.9935657
Ti	1.7904374	-1.1193203	1.2236743
C	0.8450364	-5.2071363	-0.1209247
C	1.1652304	-6.1293513	-1.1274067
C	-0.4337176	-7.6902863	-0.2037037
C	2.2735764	-2.2823793	3.9505183
C	-2.3407736	-0.3405073	0.1063323
C	-5.1503746	-0.4898893	0.1692683
C	-3.1090576	0.3733937	-0.8247047
N	-6.5435676	-0.6251053	0.2321643
O	-0.3112956	0.4460237	-0.8287007
C	-0.1194246	-5.5337193	0.8429583
O	-0.2078456	-0.9078353	0.9578853
O	1.8007474	-1.3716993	2.9862173
C	-0.8566056	-0.2602593	0.0729193
C	-2.9883596	-1.1421953	1.0608883
C	-4.3734486	-1.2275113	1.0822373
O	5.1414124	1.3625417	-2.9679197
C	7.1712044	-7.0260033	-1.1350137
C	11.3221234	1.1804407	-1.0956637
O	7.2578264	-0.4522143	0.8466603
C	4.6690174	2.2718277	-3.9336267
O	3.5957494	-1.7127783	0.9223573
Ti	5.1488144	1.1113697	-1.2048997
C	7.7996214	0.2497907	-0.0579537
C	11.4444194	-0.3256303	0.7973183
O	4.9707434	-0.7235003	-0.9938727
O	7.1533444	0.8988187	-0.9415307
Ti	5.2739074	-0.9637823	1.1771683
C	9.2899134	0.3246897	-0.0958417
C	9.9304824	1.1132197	-1.0622127
C	12.0800084	0.4621857	-0.1665837
C	6.0242804	-0.1068233	3.9571603
C	6.6603994	-4.8821333	-0.1323157
C	8.1680044	-7.2344443	-0.1775877
C	6.4181754	-5.8536293	-1.1138577
O	4.9782154	-3.4593633	-1.0083347
C	10.0531074	-0.3956433	0.8339633
O	6.0913244	-2.7899283	0.8209653
O	5.4829024	-0.9224483	2.9450713
C	5.8535404	-3.6266593	-0.1081137
C	7.6602794	-5.0946583	0.8274023
C	8.4110964	-6.2685333	0.8029293
N	-10.9188806	0.8584157	-0.0716607
C	-9.6706186	1.3456837	-0.1088357
Cu	-12.4078836	-1.5570263	0.2036763
N	-10.8358006	-0.5045443	0.1031913
C	-9.5429576	-0.8410383	0.1706683

C	-8.7404076	0.3053647	0.0409093
C	-7.2956516	0.4013327	0.0566243
O	1.4603524	0.9139217	-2.9285597
C	7.7032644	6.7614297	-0.7826577
C	-1.4425126	6.2787737	-0.8056337
O	1.9656074	3.4524107	1.0254543
C	0.9217994	0.0962307	-3.9406437
O	4.8889454	0.9083667	0.9762263
Ti	1.6660214	0.9544507	-1.1603287
C	1.0939004	3.6219017	0.1222403
C	-0.2125466	7.0283177	1.1415913
O	3.3468544	1.7040987	-0.9043657
O	0.8546484	2.7852667	-0.8061927
Ti	3.3998094	1.9728697	1.2813823
C	0.2930744	4.8817537	0.1418923
C	-0.6986526	5.1003377	-0.8248447
C	-1.2009586	7.2430297	0.1769133
C	2.2765934	2.0589757	4.0666073
C	6.1021154	5.1967537	0.1396903
C	7.3845164	7.6781137	0.2232313
C	7.0649664	5.5234057	-0.8257627
O	5.7127034	3.0764417	-0.8471457
C	0.5338454	5.8515697	1.1253623
O	4.5741874	3.6103367	1.0108513
O	3.2558204	2.0455497	3.0549943
C	5.4185704	3.8705427	0.0949743
C	5.7853444	6.1180407	1.1480783
C	6.4260604	7.3550617	1.1878453
N	-15.4612156	1.7511777	-0.2784297
C	-16.5135186	2.5676167	-0.4031757
Cu	-12.6105626	1.6998157	-0.2119907
N	-14.3102116	2.5167297	-0.3498497
C	-14.6665416	3.7916117	-0.5170737
C	-16.0907096	3.9234387	-0.5639637
C	-16.8514946	5.0732297	-0.7242427
H	-13.3415216	-4.5709743	0.5643703
H	-17.2182286	-2.6655833	0.2419413
H	-5.0844516	0.8301237	-1.5414377
H	0.7760134	-8.0806433	-1.9475407
H	4.1780864	-2.1747483	-5.0234187
H	5.3499084	-2.9141483	-3.8911907
H	5.2382414	-1.1325503	-4.0236877
H	-1.5037856	-7.0231623	1.5476953
H	1.9120484	-5.8605863	-1.8666787
H	-0.9311846	-8.6561833	-0.2359437
H	3.3695994	-2.3010223	3.9341193
H	1.8894254	-3.2872673	3.7333063
H	1.9267524	-1.9692373	4.9430203
H	-2.6018856	0.9779907	-1.5692157
H	-0.3584106	-4.8077193	1.6126203
H	-2.3877256	-1.6980553	1.7727673
H	-4.8842866	-1.8527543	1.8080273
H	6.9814574	-7.7777393	-1.8964027
H	11.8165754	1.7921167	-1.8454537
H	5.0128834	1.9552787	-4.9260957
H	5.0561914	3.2763527	-3.7199357
H	3.5730664	2.2934397	-3.9150437
H	12.0338884	-0.8850543	1.5187063
H	9.3257394	1.6628757	-1.7754197
H	13.1652954	0.5157487	-0.1940377
H	7.0900024	0.0694957	3.7627903
H	5.9118504	-0.6105103	4.9251953
H	5.4952344	0.8531157	3.9796833
H	8.7545004	-8.1494203	-0.1952367
H	5.6387024	-5.6753933	-1.8468237
H	9.5422154	-1.0036443	1.5728253
H	7.8338594	-4.3342003	1.5811093
H	9.1855004	-6.4313223	1.5476473
H	-9.4970876	2.4058627	-0.2395787

H	-9.2336776	-1.8673763	0.3072813
H	-6.8755846	1.4094907	-0.0767247
H	8.4494804	7.0122357	-1.5317157
H	-2.2098586	6.4467377	-1.5565547
H	1.4522514	-0.8629423	-3.9611657
H	1.0351184	0.5990077	-4.9090457
H	-0.1439486	-0.0814343	-3.7476497
H	-0.0238106	7.7787267	1.9045453
H	-0.8708316	4.3411307	-1.5801597
H	-1.7817276	8.1617537	0.1902733
H	1.5947274	2.9052547	3.9131713
H	2.7670714	2.1622897	5.0424273
H	1.7059144	1.1232727	4.0404873
H	7.8833174	8.6433167	0.2557123
H	7.3010474	4.7981827	-1.5970667
H	1.3071934	5.6686317	1.8636333
H	5.0396534	5.8492527	1.8884823
H	6.1791774	8.0675557	1.9702913
H	-17.5213726	2.1760377	-0.3754587
H	-13.9175996	4.5682347	-0.5983407
H	-16.3835276	6.0444247	-0.8358047
H	-17.9343816	5.0328977	-0.7423307
H	-17.2666916	-5.5520493	0.6054393
H	-15.6007236	-6.3539923	0.7417373

[$2H_2O$-CO_2]

Atom	x	y	z
N	13.8726423	-2.1575820	-0.6699909
C	14.0871783	-3.4502290	-0.9195089
Cu	15.1796363	0.3137710	-0.1732709
N	15.1005783	-1.5324350	-0.5354189
C	16.0573003	-2.4516970	-0.7045619
C	15.4886093	-3.7377320	-0.9609619
C	16.1188843	-4.9525330	-1.1910459
O	-3.8238457	-2.0387840	2.8546461
C	4.3388423	0.4767320	0.6849301
C	-0.8268007	-7.3629070	0.6522931
O	-1.3913217	-2.9357650	-1.0853799
C	-4.7297187	-2.0386010	3.9305851
O	-2.1281687	0.8758450	-1.0739189
Ti	-3.6981947	-1.8962100	1.0883211
C	-1.6842447	-3.7684050	-0.1786049
C	0.5466723	-6.6473940	-1.2101979
O	-2.1784457	-0.8306340	0.8397611
O	-2.4977207	-3.5276470	0.7741021
Ti	-1.9292057	-0.9479490	-1.3524109
C	-1.0445547	-5.1149380	-0.2203799
C	-1.4278507	-6.1062860	0.6941521
C	0.1606193	-7.6343530	-0.2987759
C	-2.3944367	-2.0156770	-4.1231299
C	2.1881023	-0.1533090	-0.2364339
C	4.9980393	-0.2420630	-0.3319199
C	2.9504423	0.5192590	0.7294871
N	6.3929743	-0.3445910	-0.4157599
O	0.1519223	0.5731620	0.7327031
C	-0.0538907	-5.3907170	-1.1734369
O	0.0576133	-0.7443330	-1.0797129
O	-1.9464717	-1.1316420	-3.1227819
C	0.7058063	-0.1033290	-0.1864379
C	2.8413293	-0.8864530	-1.2408489
C	4.2273623	-0.9410360	-1.2798899
O	-5.3172777	1.3991500	2.9032711
C	-7.2745827	-6.9658590	0.7770011
C	-11.4851327	1.2134120	1.0047161
O	-7.4034847	-0.3270700	-0.9757999
C	-4.8793097	2.3184580	3.8757031
O	-3.7388077	-1.5532900	-1.0625879
Ti	-5.3101757	1.1901820	1.1333161
C	-7.9531157	0.3425940	-0.0505679

C	-11.5904897	-0.2303620	-0.9372949
O	-5.1209307	-0.6406550	0.8786451
O	-7.3135257	0.9671570	0.8544701
Ti	-5.4213977	-0.8115030	-1.3214159
C	-9.4436857	0.4054440	-0.0157239
C	-10.0929897	1.1571990	0.9739091
C	-12.2347777	0.5208120	0.0498081
C	-6.1425537	0.1162130	-4.0868099
C	-6.7745777	-4.7869710	-0.1530259
C	-8.2586297	-7.1540970	-0.1976599
C	-6.5331517	-5.7862000	0.8005411
O	-5.1081007	-3.3812170	0.7783821
C	-10.1986287	-0.2893850	-0.9713869
O	-6.2249227	-2.6550160	-1.0264059
O	-5.6131897	-0.7242190	-3.0880759
C	-5.9805767	-3.5237940	-0.1294149
C	-7.7620267	-4.9790590	-1.1298309
C	-8.5008637	-6.1605370	-1.1503719
N	10.7425003	1.1983470	-0.0475609
C	9.4857773	1.6585280	0.0312081
Cu	12.2724593	-1.1653740	-0.4932859
N	10.6827103	-0.1513330	-0.3118899
C	9.3955723	-0.5067800	-0.3917359
C	8.5734623	0.6132200	-0.1802599
C	7.1268893	0.6823410	-0.1780389
O	-1.6332837	1.0200540	2.8522891
C	-7.9136197	6.8251640	0.8539641
C	1.2826993	6.3883700	0.8518281
O	-2.1412517	3.6110070	-1.0237809
C	-1.1269877	0.4781410	4.0503241
O	-5.0474457	1.0484960	-1.0543409
Ti	-1.8226587	1.0580570	1.0975281
C	-1.2665127	3.7599890	-0.1191359
C	0.0313203	7.1917460	-1.0600569
O	-3.5167787	1.7952270	0.8516591
O	-1.0267177	2.9034550	0.7901921
Ti	-3.5609017	2.1313670	-1.3213099
C	-0.4650967	5.0191510	-0.1135149
C	0.5376953	5.2105660	0.8475981
C	1.0308183	7.3792950	-0.1011379
C	-2.4299477	2.3072040	-4.1017489
C	-6.2933127	5.3007540	-0.1017109
C	-7.5952607	7.7731370	-0.1226229
C	-7.2655297	5.5915770	0.8658761
O	-5.8898107	3.1574990	0.8287151
C	-0.7158187	6.0154300	-1.0676939
O	-4.7495737	3.7508280	-1.0098579
O	-3.4087347	2.2501280	-3.0909169
C	-5.5991957	3.9797280	-0.0906289
C	-5.9771027	6.2533510	-1.0808349
C	-6.6275137	7.4858710	-1.0892919
N	15.2692603	2.1594250	0.1892011
C	16.3075703	2.9848520	0.3628421
Cu	12.4194753	2.0596080	0.1382441
N	14.1051903	2.8967540	0.3203051
C	14.4396723	4.1635800	0.5722841
C	15.8615493	4.3183810	0.6189551
C	16.6025753	5.4684970	0.8528021
H	13.2554473	-4.1277060	-1.0587949
H	17.1019683	-2.1786080	-0.6407669
H	4.9224443	0.9668210	1.4589621
H	-1.1285447	-8.1306610	1.3592801
H	-4.1750547	-2.0793260	4.8757241
H	-5.3884957	-2.9131540	3.8600821
H	-5.3327947	-1.1244730	3.8992641
H	1.3170693	-6.8574120	-1.9469229
H	-2.2167637	-5.8974790	1.4091901
H	0.6295243	-8.6142830	-0.3286219
H	-3.4904237	-2.0411880	-4.1285749

H	-2.0077827	-3.0241280	-3.9286429
H	-2.0306677	-1.6702660	-5.0985979
H	2.4378113	1.0582120	1.5187911
H	0.2374453	-4.6095820	-1.8670379
H	2.2454823	-1.4160370	-1.9763319
H	4.7436943	-1.5129200	-2.0447509
H	-7.0860487	-7.7386500	1.5172171
H	-11.9863747	1.7964700	1.7725131
H	-5.1937957	1.9725280	4.8679691
H	-5.3194507	3.3044640	3.6797071
H	-3.7862677	2.3979130	3.8436731
H	-12.1735847	-0.7697690	-1.6787659
H	-9.4948337	1.6874400	1.7070861
H	-13.3204617	0.5657850	0.0752301
H	-7.2136617	0.2755930	-3.9089169
H	-6.0051067	-0.3580770	-5.0661819
H	-5.6228737	1.0811860	-4.0699219
H	-8.8359797	-8.0747930	-0.2148619
H	-5.7649257	-5.6239310	1.5488881
H	-9.6813787	-0.8690480	-1.7283689
H	-7.9356457	-4.1973090	-1.8614019
H	-9.2653057	-6.3075580	-1.9084369
H	9.2939893	2.7042200	0.2337711
H	9.1076503	-1.5280330	-0.5949119
H	6.6891303	1.6709400	0.0261211
H	-8.6670477	7.0482020	1.6045311
H	2.0592863	6.5346490	1.5976761
H	-0.4978237	-0.3900320	3.8237641
H	-1.9653677	0.1793660	4.6921621
H	-0.5365377	1.2452400	4.5671661
H	-0.1651627	7.9627630	-1.8001339
H	0.7190103	4.4305110	1.5791161
H	1.6125113	8.2974790	-0.0962089
H	-1.7537367	3.1514490	-3.9164989
H	-2.9217357	2.4435540	-5.0727389
H	-1.8537877	1.3746900	-4.1099949
H	-8.1015747	8.7348890	-0.1306569
H	-7.5014387	4.8427230	1.6143031
H	-1.4971667	5.8534250	-1.8024269
H	-5.2240517	6.0124300	-1.8233659
H	-6.3808627	8.2227340	-1.8488399
H	17.3220963	2.6148090	0.3021161
H	13.6773793	4.9186900	0.7115261
H	16.1179283	6.4208720	1.0338611
H	17.6861363	5.4472550	0.8609921
H	17.1996783	-5.0335360	-1.1951019
H	15.5474003	-5.8548130	-1.3754949
O	0.1896283	-2.1004120	2.4006921
H	-0.4810027	-1.6771490	1.8384921
H	-0.3292977	-2.7220960	2.9533131
O	-1.6900417	-3.8389780	3.6582641
H	-2.4076767	-3.1950970	3.5012291
H	-1.6976407	-4.3488630	2.8324611
C	10.6288173	-3.9307880	1.4096681
O	10.6846013	-3.7732690	0.2489441
O	10.5751283	-4.0884400	2.5644721

[H_2O-OH-COOH]

Atom	x	y	z
N	13.7673886	-2.1639287	-0.6689522
C	13.9830306	-3.4576427	-0.9121492
Cu	15.0720446	0.3114013	-0.1863182
N	14.9947036	-1.5367207	-0.5389392
C	15.9522946	-2.4557347	-0.7044812
C	15.3848026	-3.7436727	-0.9536682
C	16.0162066	-4.9589567	-1.1782282
O	-3.9260394	-2.0497587	2.8742898
C	4.2320236	0.4663763	0.6830438
C	-0.9246624	-7.3792147	0.6961318

O	-1.4961714	-2.9616477	-1.0634832
C	-4.8297964	-2.0411997	3.9517508
O	-2.2373734	0.8491293	-1.0706582
Ti	-3.8013254	-1.9127757	1.1075408
C	-1.7871394	-3.7899807	-0.1521462
C	0.4459356	-6.6716517	-1.1715042
O	-2.2836684	-0.8474987	0.8517648
O	-2.5997664	-3.5452367	0.8002288
Ti	-2.0366824	-0.9757167	-1.3400542
C	-1.1459544	-5.1359797	-0.1877572
C	-1.5271074	-6.1230837	0.7322458
C	0.0620196	-7.6543847	-0.2546302
C	-2.5036844	-2.0582207	-4.1047762
C	2.0809936	-0.1708117	-0.2327112
C	4.8909236	-0.2568487	-0.3308532
C	2.8436246	0.5075453	0.7289218
N	6.2858826	-0.3582117	-0.4157142
O	0.0450506	0.5582723	0.7349608
C	-0.1560244	-5.4154897	-1.1404922
O	-0.0497434	-0.7685637	-1.0706042
O	-2.0556324	-1.1685797	-3.1094492
C	0.5986966	-0.1222687	-0.1813292
C	2.7339546	-0.9083297	-1.2340952
C	4.1200036	-0.9615297	-1.2743912
O	-5.4226984	1.3890913	2.9073398
C	-7.3727544	-6.9888897	0.8259528
C	-11.5924244	1.1866303	1.0165878
O	-7.5111954	-0.3592857	-0.9605602
C	-4.9847134	2.3138493	3.8745818
O	-3.8452134	-1.5817527	-1.0451462
Ti	-5.4173054	1.1710993	1.1384678
C	-8.0605744	0.3144653	-0.0381492
C	-11.6982654	-0.2671567	-0.9179112
O	-5.2263064	-0.6608687	0.8929228
O	-7.4207054	0.9443693	0.8629808
Ti	-5.5289944	-0.8433347	-1.3059062
C	-9.5511774	0.3757913	-0.0019752
C	-10.2002524	1.1318463	0.9845258
C	-12.3423254	0.4883083	0.0660568
C	-6.2542014	0.0695503	-4.0751852
C	-6.8762714	-4.8142087	-0.1157352
C	-8.3576574	-7.1832217	-0.1466442
C	-6.6326514	-5.8082817	0.8426518
O	-5.2103334	-3.4018647	0.8066428
C	-10.3063744	-0.3247667	-0.9532442
O	-6.3300234	-2.6861137	-1.0005902
O	-5.7227734	-0.7651687	-3.0727622
C	-6.0836934	-3.5500207	-0.0994492
C	-7.8645754	-5.0124067	-1.0904522
C	-8.6020794	-6.1948157	-1.1041462
N	10.6340396	1.1915463	-0.0602082
C	9.3768766	1.6506893	0.0176008
Cu	12.1661996	-1.1726957	-0.4955702
N	10.5755136	-0.1595217	-0.3175782
C	9.2886886	-0.5168487	-0.3941922
C	8.4655276	0.6032743	-0.1875202
C	7.0188796	0.6707583	-0.1840492
O	-1.7383304	1.0139413	2.8542138
C	-8.0275144	6.8016083	0.8332458
C	1.1692926	6.3752993	0.8231568
O	-2.2535364	3.5844943	-1.0344632
C	-1.2300944	0.4787303	4.0544358
O	-5.1568234	1.0185473	-1.0487282
Ti	-1.9296804	1.0427733	1.0994918
C	-1.3779734	3.7390893	-0.1315592
C	-0.0851124	7.1674773	-1.0914172
O	-3.6249154	1.7767423	0.8517398
O	-1.1361954	2.8874843	0.7818618
Ti	-3.6718154	2.1017363	-1.3228642

C	-0.5779954	4.9991773	-0.1332522
C	0.4256336	5.1966403	0.8257628
C	0.9152256	7.3610583	-0.1345752
C	-2.5441294	2.2646693	-4.1054172
C	-6.4065134	5.2741903	-0.1164312
C	-7.7113174	7.7449463	-0.1485182
C	-7.3779964	5.5688373	0.8507348
O	-5.9995304	3.1361733	0.8244728
C	-0.8309104	5.9902873	-1.0922242
O	-4.8620004	3.7214113	-1.0183662
O	-3.5217354	2.2116383	-3.0932232
C	-5.7108724	3.9540293	-0.0993762
C	-6.0924744	6.2221363	-1.1007532
C	-6.7443064	7.4538543	-1.1147782
N	15.1599536	2.1589833	0.1666318
C	16.1975056	2.9864703	0.3349088
Cu	12.3102296	2.0556553	0.1193408
N	13.9951836	2.8956423	0.2952608
C	14.3284906	4.1641193	0.5404008
C	15.7502406	4.3207773	0.5847058
C	16.4902046	5.4729173	0.8118608
H	13.1516256	-4.1350557	-1.0565802
H	16.9967416	-2.1795427	-0.6445492
H	4.8160876	0.9622093	1.4529958
H	-1.2214134	-8.1420757	1.4106108
H	-4.2692954	-2.0561107	4.8936858
H	-5.4740914	-2.9275767	3.8983718
H	-5.4466754	-1.1369337	3.9041378
H	1.2163526	-6.8844857	-1.9075732
H	-2.2902734	-5.8907297	1.4670478
H	0.5333736	-8.6334397	-0.2797512
H	-3.5996584	-2.0827227	-4.1102532
H	-2.1179844	-3.0656257	-3.9035422
H	-2.1390334	-1.7186937	-5.0819572
H	2.3316976	1.0538243	1.5137758
H	0.1323806	-4.6398847	-1.8417922
H	2.1382156	-1.4410787	-1.9674262
H	4.6362406	-1.5369567	-2.0365292
H	-7.1820074	-7.7577997	1.5696188
H	-12.0934804	1.7728673	1.7820848
H	-5.2811924	1.9628783	4.8704698
H	-5.4406874	3.2938823	3.6847188
H	-3.8932954	2.4083363	3.8285268
H	-12.2815374	-0.8111937	-1.6558512
H	-9.6019094	1.6662753	1.7144988
H	-13.4280384	0.5320553	0.0925408
H	-7.3251094	0.2293343	-3.8964222
H	-6.1182094	-0.4100087	-5.0522022
H	-5.7351104	1.0349473	-4.0644942
H	-8.9338814	-8.1047367	-0.1586642
H	-5.8623544	-5.6416797	1.5879528
H	-9.7892914	-0.9079877	-1.7075992
H	-8.0399334	-4.2347047	-1.8259342
H	-9.3670924	-6.3466657	-1.8607252
H	9.1843546	2.6972013	0.2155588
H	9.0041066	-1.5431327	-0.5757732
H	6.5802656	1.6601243	0.0151178
H	-8.7803904	7.0275733	1.5834848
H	1.9462856	6.5264313	1.5676118
H	-1.3478354	-0.6103517	4.0733318
H	-1.7705204	0.9227553	4.8997518
H	-0.1649524	0.7268363	4.1374458
H	-0.2834064	7.9345203	-1.8351232
H	0.6081546	4.4209673	1.5616178
H	1.4957726	8.2799773	-0.1348742
H	-1.8692714	3.1112523	-3.9258602
H	-3.0372304	2.3945003	-5.0766212
H	-1.9663174	1.3331493	-4.1090092
H	-8.2187714	8.7060533	-0.1609192

H	-7.6122324	4.8235203	1.6032008
H	-1.6129684	5.8236793	-1.8251682
H	-5.3400204	5.9782683	-1.8429292
H	-6.4993894	8.1870983	-1.8783872
H	17.2127836	2.6170293	0.2749048
H	13.5655266	4.9193203	0.6766728
H	16.0053776	6.4265263	0.9888718
H	17.5744056	5.4551023	0.8195988
H	17.0974226	-5.0401557	-1.1824592
H	15.4459836	-5.8634147	-1.3577422
O	-0.0258774	-2.2299117	2.1406268
H	-0.7706114	-1.7440687	1.7033588
O	-1.4938984	-3.0336717	4.1352028
H	-2.3252834	-2.8626917	3.6558718
H	-0.8616594	-3.2519617	3.4223318
C	11.3484476	-3.6644647	1.6267488
O	12.5451946	-3.3424427	2.0832788
O	10.8748156	-3.5172197	0.5344228
H	13.0814066	-2.9499917	1.3475508

[H_2O-OH-HCOO]

Atom	x	y	z
N	13.7706017	-2.0824163	-0.5373465
C	13.9921107	-3.3781463	-0.7638405
Cu	15.0594517	0.4048547	-0.0778785
N	14.9932347	-1.4477933	-0.4083515
C	15.9562947	-2.3639383	-0.5572955
C	15.3966197	-3.6574363	-0.7937175
C	16.0352437	-4.8723973	-1.0000765
O	-3.9435363	-2.0141503	2.9060665
C	4.2140977	0.5159907	0.7294695
C	-0.9036643	-7.3546473	0.8098275
O	-1.4871803	-2.9617783	-1.0067595
C	-4.8533633	-1.9970353	3.9783085
O	-2.2471873	0.8448597	-1.0645895
Ti	-3.8096163	-1.8980953	1.1384895
C	-1.7791413	-3.7803933	-0.0869965
C	0.4738507	-6.6630263	-1.0586915
O	-2.2958563	-0.8284503	0.8781305
O	-2.5982923	-3.5281433	0.8577995
Ti	-2.0359573	-0.9820933	-1.3105585
C	-1.1311093	-5.1234903	-0.1026105
C	-1.5125153	-6.1012093	0.8272555
C	0.0896767	-7.6364273	-0.1320215
C	-2.4821143	-2.1005483	-4.0643965
C	2.0714117	-0.1431023	-0.1903355
C	4.8822397	-0.2162193	-0.2718515
C	2.8252757	0.5507457	0.7671495
N	6.2781347	-0.3116053	-0.3477415
O	0.0264957	0.5874917	0.7570645
C	-0.1344863	-5.4096183	-1.0463615
O	-0.0516123	-0.7617133	-1.0326555
O	-2.0440323	-1.1966173	-3.0775415
C	0.5886277	-0.1013773	-0.1477635
C	2.7336157	-0.8894823	-1.1790035
C	4.1201147	-0.9362113	-1.2109675
O	-5.4573823	1.4172657	2.8888075
C	-7.3542363	-6.9951443	0.8991465
C	-11.6153433	1.1608047	0.9665195
O	-7.5154993	-0.3885843	-0.9689565
C	-5.0294053	2.3559307	3.8470935
O	-3.8430933	-1.5935693	-1.0182935
Ti	-5.4410023	1.1777617	1.1227875
C	-8.0733653	0.2935917	-0.0579025
C	-11.7031473	-0.3169633	-0.9506325
O	-5.2395653	-0.6560853	0.9007015
O	-7.4416803	0.9376357	0.8389965
Ti	-5.5290253	-0.8668563	-1.2973785
C	-9.5644333	0.3478697	-0.0307385

C	-10.2227603	1.1126247	0.9428425
C	-12.3564423	0.4471937	0.0204505
C	-6.2432193	0.0085637	-4.0815765
C	-6.8632633	-4.8296363	-0.0662705
C	-8.3327023	-7.2062573	-0.0764455
C	-6.6200943	-5.8107193	0.9055225
O	-5.2095303	-3.3978123	0.8480005
C	-10.3108123	-0.3680113	-0.9775475
O	-6.3226133	-2.7097693	-0.9740165
O	-5.7132873	-0.8111973	-3.0661025
C	-6.0770593	-3.5613813	-0.0610395
C	-7.8450983	-5.0446583	-1.0439545
C	-8.5766493	-6.2308343	-1.0472885
N	10.6165057	1.2641827	0.0128625
C	9.3566677	1.7179167	0.0781355
Cu	12.1631077	-1.0978463	-0.3850025
N	10.5660157	-0.0903733	-0.2283715
C	9.2815327	-0.4548703	-0.3075935
C	8.4516657	0.6635117	-0.1192505
C	7.0047217	0.7237737	-0.1245965
O	-1.7709603	1.0599967	2.8606805
C	-8.0773133	6.7909577	0.7343535
C	1.1214047	6.4107327	0.7804225
O	-2.2771033	3.5803457	-1.0619015
C	-1.2668113	0.5420117	4.0701485
O	-5.1675223	0.9998727	-1.0609045
Ti	-1.9526403	1.0664857	1.1046905
C	-1.4073873	3.7503277	-0.1561165
C	-0.1261653	7.1732097	-1.1506055
O	-3.6500553	1.7888557	0.8386175
O	-1.1665063	2.9111417	0.7689705
Ti	-3.6863853	2.0870817	-1.3400205
C	-0.6136633	5.0143017	-0.1687705
C	0.3835897	5.2284747	0.7933065
C	0.8678267	7.3834577	-0.1906725
C	-2.5439533	2.2217487	-4.1180675
C	-6.4434743	5.2602357	-0.1875995
C	-7.7602973	7.7238347	-0.2570935
C	-7.4218053	5.5617677	0.7704985
O	-6.0311843	3.1359147	0.7815915
C	-0.8661083	5.9923687	-1.1411635
O	-4.8862633	3.7043527	-1.0619295
O	-3.5269373	2.1761507	-3.1107315
C	-5.7414103	3.9438897	-0.1505665
C	-6.1286263	6.1976797	-1.1816725
C	-6.7864613	7.4258467	-1.2143535
N	15.1362317	2.2570187	0.2529615
C	16.1687147	3.0916917	0.4168695
Cu	12.2873637	2.1388147	0.1911575
N	13.9671267	2.9893327	0.3661345
C	14.2927697	4.2623607	0.5976055
C	15.7134547	4.4266817	0.6478675
C	16.4464207	5.5852047	0.8650265
H	13.4164517	-3.7745753	-1.6103195
H	16.9994887	-2.0887953	-0.4737825
H	4.7912137	1.0199197	1.4997355
H	-1.1981393	-8.1089963	1.5344525
H	-4.2957583	-2.0206483	4.9219345
H	-5.5079973	-2.8761533	3.9212855
H	-5.4606163	-1.0858303	3.9306055
H	1.2511957	-6.8803773	-1.7865085
H	-2.2743633	-5.8585933	1.5598225
H	0.5670717	-8.6131473	-0.1417195
H	-3.5780753	-2.1292143	-4.0780785
H	-2.0950653	-3.1042353	-3.8469955
H	-2.1117053	-1.7734333	-5.0441255
H	2.3042047	1.0960137	1.5463835
H	0.1566397	-4.6384753	-1.7513635
H	2.1430917	-1.4385373	-1.9042775

H	4.6431407	-1.5209913	-1.9615045
H	-7.1633053	-7.7541313	1.6529945
H	-12.1236873	1.7535877	1.7222485
H	-5.3379323	2.0203157	4.8447905
H	-5.4810493	3.3342727	3.6375225
H	-3.9372563	2.4468827	3.8126925
H	-12.2796783	-0.8732313	-1.6848815
H	-9.6303333	1.6581267	1.6694495
H	-13.4426013	0.4856137	0.0405365
H	-7.3157843	0.1673617	-3.9104965
H	-6.1011763	-0.4825523	-5.0522795
H	-5.7277273	0.9760987	-4.0813405
H	-8.9042323	-8.1309233	-0.0805685
H	-5.8538243	-5.6303733	1.6518025
H	-9.7857943	-0.9579533	-1.7211245
H	-8.0194243	-4.2764953	-1.7896725
H	-9.3366833	-6.3959543	-1.8063145
H	9.1543837	2.7652557	0.2625355
H	9.0054437	-1.4843163	-0.4855985
H	6.5600507	1.7124337	0.0647215
H	-8.8355783	7.0223087	1.4776215
H	1.8932207	6.5748907	1.5277065
H	-1.3331063	-0.5521623	4.0833115
H	-1.8450253	0.9620437	4.9035925
H	-0.2158163	0.8388247	4.1756675
H	-0.3242933	7.9304587	-1.9045405
H	0.5645867	4.4617417	1.5388865
H	1.4434587	8.3056447	-0.1987835
H	-1.8723633	3.0724027	-3.9441765
H	-3.0316433	2.3394687	-5.0938805
H	-1.9630863	1.2920687	-4.1082715
H	-8.2725463	8.6822237	-0.2840785
H	-7.6556593	4.8239267	1.5304175
H	-1.6434843	5.8119427	-1.8758395
H	-5.3706753	5.9476347	-1.9161775
H	-6.5409753	8.1510807	-1.9855535
H	17.1860727	2.7272947	0.3672015
H	13.5250507	5.0145317	0.7204345
H	15.9537847	6.5370907	1.0272565
H	17.5303347	5.5709067	0.8787495
H	17.1167827	-4.9469743	-0.9681385
H	15.4647017	-5.7785853	-1.1699765
O	0.1341267	-2.2852983	2.1430745
H	-0.5849403	-1.8233583	1.6704835
O	-1.3515763	-2.8413903	4.1974885
H	-2.2056833	-2.7595983	3.7389865
H	-0.6923063	-2.8248043	3.4342535
C	12.1125017	-4.2486593	0.5226685
O	13.4340407	-4.2617773	0.3950175
O	11.3223937	-3.5919573	-0.1306885
H	11.8047297	-4.9364523	1.3268725

[2OH-CO]

Atom	x	y	z
N	13.7395906	-2.1611427	-0.4496452
C	13.9560966	-3.4589047	-0.6701692
Cu	15.0428516	0.3197333	0.0071488
N	14.9665836	-1.5337307	-0.3171762
C	15.9244686	-2.4563207	-0.4583252
C	15.3577266	-3.7473737	-0.6929362
C	15.9897006	-4.9667667	-0.8919722
O	-3.9878864	-1.9668607	2.9113118
C	4.1947826	0.5017663	0.7635498
C	-0.9726944	-7.3362837	0.8477428
O	-1.5201414	-2.9463117	-0.9869782
C	-4.9035764	-1.9444107	3.9787018
O	-2.2559224	0.8647883	-1.0617402
Ti	-3.8463354	-1.8613757	1.1435678
C	-1.8215074	-3.7598127	-0.0657122

C	0.4177936	-6.6599507	-1.0167532
O	-2.3240234	-0.8013817	0.8866018
O	-2.6435144	-3.4991397	0.8743528
Ti	-2.0549814	-0.9644627	-1.3002972
C	-1.1818654	-5.1070017	-0.0735872
C	-1.5737374	-6.0789937	0.8579388
C	0.0232116	-7.6276287	-0.0884662
C	-2.4954404	-2.0897147	-4.0522292
C	2.0522846	-0.1471367	-0.1639042
C	4.8629396	-0.2381867	-0.2320722
C	2.8060466	0.5453603	0.7946408
N	6.2585466	-0.3425897	-0.3010582
O	0.0076306	0.5996273	0.7713818
C	-0.1827044	-5.4027147	-1.0116682
O	-0.0706624	-0.7553997	-1.0139242
O	-2.0562844	-1.1850437	-3.0665322
C	0.5696096	-0.0959637	-0.1283862
C	2.7143576	-0.9011587	-1.1468402
C	4.1006696	-0.9566887	-1.1721832
O	-5.4807464	1.4713383	2.8746158
C	-7.4212244	-6.9360097	0.9057408
C	-11.6312184	1.2466953	0.9245678
O	-7.5322694	-0.3352377	-0.9863192
C	-5.0513264	2.4106933	3.8315788
O	-3.8673234	-1.5634047	-1.0143042
Ti	-5.4576854	1.2254733	1.1095458
C	-8.0900534	0.3536533	-0.0802812
C	-11.7194224	-0.2372857	-0.9877642
O	-5.2666864	-0.6102957	0.8948628
O	-7.4584964	0.9968983	0.8172828
Ti	-5.5472814	-0.8269967	-1.3037852
C	-9.5808594	0.4173803	-0.0602572
C	-10.2388704	1.1896973	0.9075508
C	-12.3723954	0.5343983	-0.0224282
C	-6.2430944	0.0428813	-4.0943562
C	-6.9121694	-4.7770627	-0.0649882
C	-8.3964644	-7.1444417	-0.0736512
C	-6.6796854	-5.7561987	0.9113718
O	-5.2537794	-3.3523547	0.8519348
C	-10.3273284	-0.2971597	-1.0080102
O	-6.3539934	-2.6638617	-0.9776572
O	-5.7230444	-0.7765827	-3.0735452
C	-6.1180374	-3.5137557	-0.0605532
C	-7.8907944	-4.9893887	-1.0464752
C	-8.6297684	-6.1709557	-1.0490462
N	10.6050226	1.2072193	0.0742228
C	9.3477766	1.6690833	0.1319878
Cu	12.1382286	-1.1654857	-0.3082242
N	10.5472316	-0.1476667	-0.1624182
C	9.2607406	-0.5045917	-0.2466482
C	8.4371216	0.6197073	-0.0658822
C	6.9905896	0.6890053	-0.0782492
O	-1.7965564	1.0908553	2.8649248
C	-8.0568094	6.8536833	0.6890878
C	1.1390216	6.4159293	0.7793598
O	-2.2686404	3.6004023	-1.0688132
C	-1.3012894	0.5740103	4.0785448
O	-5.1752044	1.0381303	-1.0722102
Ti	-1.9700274	1.0922473	1.1081098
C	-1.4020814	3.7681363	-0.1595902
C	-0.0947584	7.1793653	-1.1601302
O	-3.6616314	1.8243123	0.8315758
O	-1.1707844	2.9307413	0.7695498
Ti	-3.6859804	2.1150313	-1.3482442
C	-0.6003734	5.0270523	-0.1729892
C	0.3937336	5.2383753	0.7929638
C	0.8960736	7.3867773	-0.1963212
C	-2.5298514	2.2326743	-4.1213902
C	-6.4283734	5.3094823	-0.2198512

C	-7.7293394	7.7810343	-0.3041452
C	-7.4092234	5.6205433	0.7326108
O	-6.0339514	3.1860683	0.7587168
C	-0.8421504	6.0032293	-1.1499842
O	-4.8769394	3.7407693	-1.0814352
O	-3.5177644	2.1968153	-3.1184942
C	-5.7347804	3.9888993	-0.1749172
C	-6.1030284	6.2414013	-1.2157382
C	-6.7529664	7.4735483	-1.2558052
N	15.1297416	2.1725333	0.3318528
C	16.1666856	3.0012963	0.4975998
Cu	12.2805066	2.0719923	0.2571538
N	13.9647536	2.9125693	0.4369768
C	14.2973196	4.1843443	0.6654738
C	15.7187626	4.3399273	0.7217838
C	16.4579846	5.4945913	0.9382828
H	13.1253166	-4.1328587	-0.8326802
H	16.9688086	-2.1825857	-0.3898202
H	4.7719776	1.0116993	1.5295678
H	-1.2757594	-8.0855097	1.5737108
H	-4.3555524	-2.0064827	4.9261718
H	-5.5882904	-2.7983137	3.8988548
H	-5.4793724	-1.0130117	3.9456638
H	1.1949046	-6.8853937	-1.7419762
H	-2.3275774	-5.8270797	1.5962048
H	0.4934926	-8.6075227	-0.0935492
H	-3.5913394	-2.1154307	-4.0665402
H	-2.1108664	-3.0939547	-3.8332852
H	-2.1232464	-1.7648527	-5.0316362
H	2.2869666	1.1096793	1.5620878
H	0.1115226	-4.6395747	-1.7242642
H	2.1247156	-1.4423827	-1.8789192
H	4.6240966	-1.5432997	-1.9208102
H	-7.2389634	-7.6933307	1.6632998
H	-12.1392444	1.8455893	1.6755548
H	-5.3629644	2.0787353	4.8295038
H	-5.4998004	3.3895743	3.6190798
H	-3.9588914	2.4995913	3.7983608
H	-12.2959474	-0.7921487	-1.7229552
H	-9.6471114	1.7348373	1.6349668
H	-13.4582684	0.5799213	-0.0077142
H	-7.3156054	0.2067583	-3.9292392
H	-6.0977334	-0.4519947	-5.0623862
H	-5.7229244	1.0077733	-4.0934842
H	-8.9738434	-8.0653197	-0.0770162
H	-5.9164034	-5.5793387	1.6615108
H	-9.8033254	-0.8928067	-1.7477382
H	-8.0575364	-4.2230817	-1.7957932
H	-9.3872934	-6.3337737	-1.8108772
H	9.1538236	2.7187163	0.3115698
H	8.9759196	-1.5293447	-0.4372922
H	6.5511976	1.6822283	0.0987148
H	-8.8170354	7.0923703	1.4279088
H	1.9085796	6.5780843	1.5292538
H	-1.1852794	-0.5134267	4.0031218
H	-2.0024054	0.8152513	4.8869208
H	-0.3292784	1.0332953	4.2981128
H	-0.2843924	7.9348873	-1.9177642
H	0.5671656	4.4741303	1.5428228
H	1.4778466	8.3048863	-0.2051682
H	-1.8533744	3.0789683	-3.9463812
H	-3.0125304	2.3514693	-5.0992582
H	-1.9557604	1.2989383	-4.1067032
H	-8.2353464	8.7424373	-0.3368912
H	-7.6521184	4.8873213	1.4941628
H	-1.6170074	5.8258563	-1.8880232
H	-5.3434944	5.9847663	-1.9463062
H	-6.4993464	8.1943493	-2.0283832
H	17.1816196	2.6300853	0.4528918

H	13.5340426	4.9424673	0.7809648
H	15.9720596	6.4507993	1.0939978
H	17.5414416	5.4735563	0.9561118
H	17.0705686	-5.0475807	-0.8867802
H	15.4193026	-5.8720337	-1.0648302
O	-0.9034124	-3.1130547	3.6575858
H	-0.7547364	-3.3729497	2.7296088
O	-2.1200354	-3.8516047	3.9501098
H	-2.7854974	-3.2322617	3.5797688
C	10.5030166	-4.3926857	-1.7560082
O	11.0437476	-3.1558557	1.0488138
O	9.4637666	-3.9534127	-1.8976172
H	11.9083276	-3.3930147	1.4215988
H	10.6263056	-2.5927367	1.7202018

[2OH-HCOOH]

Atom	x	y	z
N	13.7176238	-2.1168807	-0.6664894
C	13.9341508	-3.4082667	-0.9208254
Cu	15.0207728	0.3544853	-0.1599044
N	14.9445758	-1.4904497	-0.5290834
C	15.9027178	-2.4076517	-0.7013454
C	15.3360218	-3.6935407	-0.9628354
C	15.9681748	-4.9065317	-1.1972834
O	-3.9802792	-2.0373897	2.8518326
C	4.1794278	0.4982053	0.6947596
C	-0.9746252	-7.3488587	0.6304076
O	-1.5450272	-2.9159947	-1.0906724
C	-4.8866712	-2.0429077	3.9273496
O	-2.2875022	0.8944533	-1.0650394
Ti	-3.8542992	-1.8881077	1.0860676
C	-1.8370602	-3.7524997	-0.1871724
C	0.3984898	-6.6242627	-1.2288244
O	-2.3359752	-0.8193527	0.8421346
O	-2.6512612	-3.5165627	0.7661266
Ti	-2.0857332	-0.9279957	-1.3503704
C	-1.1953652	-5.0979267	-0.2338144
C	-1.5775592	-6.0932897	0.6768186
C	0.0135508	-7.6152387	-0.3212994
C	-2.5483522	-1.9858717	-4.1252954
C	2.0299688	-0.1315057	-0.2298124
C	4.8400658	-0.2157547	-0.3245574
C	2.7909478	0.5385183	0.7389476
N	6.2351838	-0.3159077	-0.4082524
O	-0.0076482	0.5882823	0.7412946
C	-0.2039432	-5.3686217	-1.1875294
O	-0.0993302	-0.7224677	-1.0761414
O	-2.1020692	-1.1049777	-3.1214314
C	0.5475798	-0.0839017	-0.1801934
C	2.6846548	-0.8598717	-1.2367494
C	4.0707808	-0.9122617	-1.2754654
O	-5.4788782	1.3979673	2.9128856
C	-7.4230352	-6.9617927	0.7541586
C	-11.6457372	1.2103393	1.0112796
O	-7.5610732	-0.3166017	-0.9735014
C	-5.0426362	2.3142243	3.8889656
O	-3.8945582	-1.5370797	-1.0635364
Ti	-5.4708012	1.1957243	1.1421546
C	-8.1120412	0.3487383	-0.0459474
C	-11.7482312	-0.2262127	-0.9362324
O	-5.2787552	-0.6338477	0.8806146
O	-7.4737152	0.9708053	0.8616986
Ti	-5.5781452	-0.7967987	-1.3201934
C	-9.6027152	0.4092573	-0.0114354
C	-10.2535012	1.1562953	0.9807936
C	-12.3939992	0.5202613	0.0534656
C	-6.2996242	0.1403373	-4.0823264
C	-6.9258952	-4.7786587	-0.1674064
C	-8.4064362	-7.1477837	-0.2215854

C	-6.6833562	-5.7811397	0.7824566
O	-5.2618532	-3.3739877	0.7699636
C	-10.3562722	-0.2830537	-0.9700164
O	-6.3790582	-2.6425987	-1.0324854
O	-5.7693972	-0.7030967	-3.0865824
C	-6.1337682	-3.5144107	-0.1387014
C	-7.9126932	-4.9684967	-1.1453114
C	-8.6497782	-6.1509757	-1.1706164
N	10.5822888	1.2320353	-0.0325364
C	9.3248588	1.6900593	0.0474946
Cu	12.1158878	-1.1277437	-0.4866454
N	10.5245978	-0.1167087	-0.3020044
C	9.2380088	-0.4737567	-0.3836964
C	8.4141668	0.6442173	-0.1682864
C	6.9674918	0.7111973	-0.1663554
O	-1.7943112	1.0244983	2.8618766
C	-8.0824532	6.8278803	0.8831776
C	1.1145008	6.4046563	0.8829066
O	-2.3046402	3.6293833	-1.0045324
C	-1.2876682	0.4787933	4.0580416
O	-5.2070372	1.0627243	-1.0459244
Ti	-1.9830802	1.0688783	1.1072006
C	-1.4304622	3.7762223	-0.0989954
C	-0.1373412	7.2134333	-1.0263974
O	-3.6781932	1.8044773	0.8634796
O	-1.1897472	2.9166003	0.8071696
Ti	-3.7219922	2.1487913	-1.3082144
C	-0.6309082	5.0365333	-0.0882924
C	0.3712368	5.2257803	0.8739246
C	0.8615168	7.3988173	-0.0663914
C	-2.5902512	2.3368403	-4.0875334
C	-6.4595382	5.3094973	-0.0776514
C	-7.7651252	7.7800203	-0.0896844
C	-7.4325482	5.5952143	0.8906596
O	-6.0532242	3.1633253	0.8447946
C	-0.8827412	6.0360553	-1.0387824
O	-4.9131702	3.7653043	-0.9910784
O	-3.5693332	2.2744873	-3.0773014
C	-5.7634762	3.9894643	-0.0713144
C	-6.1443652	6.2662663	-1.0530344
C	-6.7965902	7.4978493	-1.0570664
N	15.1075358	2.1988793	0.2095986
C	16.1445618	3.0251733	0.3867666
Cu	12.2579218	2.0950543	0.1571746
N	13.9423298	2.9339893	0.3430536
C	14.2748468	4.2003433	0.5999626
C	15.6964768	4.3570613	0.6477646
C	16.4357158	5.5073733	0.8862556
H	13.1015558	-4.0774287	-1.0937794
H	16.9470788	-2.1334727	-0.6368094
H	4.7625888	0.9899283	1.4681046
H	-1.2701522	-8.1162867	1.3402986
H	-4.3304532	-2.1299267	4.8680446
H	-5.5723512	-2.8943377	3.8310086
H	-5.4624772	-1.1109397	3.9237246
H	1.1696348	-6.8303317	-1.9660814
H	-2.3252612	-5.8610137	1.4276756
H	0.4853228	-8.5938677	-0.3546384
H	-3.6443182	-2.0119177	-4.1318864
H	-2.1617152	-2.9948767	-3.9335814
H	-2.1837662	-1.6372657	-5.0993924
H	2.2776878	1.0827593	1.5246096
H	0.0829128	-4.5875957	-1.8835584
H	2.0895048	-1.3839297	-1.9768374
H	4.5886868	-1.4792077	-2.0428504
H	-7.2329752	-7.7372187	1.4912086
H	-12.1481552	1.7896173	1.7811906
H	-5.3436122	1.9563893	4.8812066
H	-5.4957692	3.2968423	3.7055666

H	-3.9508182	2.4070743	3.8474016
H	-12.3302552	-0.7638187	-1.6798674
H	-9.6562862	1.6843883	1.7162816
H	-13.4797772	0.5634413	0.0787136
H	-7.3710472	0.2977103	-3.9042824
H	-6.1614072	-0.3300237	-5.0635404
H	-5.7813782	1.1060463	-4.0618584
H	-8.9823562	-8.0693427	-0.2426534
H	-5.9138762	-5.6214207	1.5301016
H	-9.8377522	-0.8592557	-1.7287664
H	-8.0871752	-4.1842417	-1.8740164
H	-9.4136542	-6.2963347	-1.9296344
H	9.1311998	2.7348043	0.2540286
H	8.9592758	-1.4995537	-0.5759124
H	6.5279268	1.6988553	0.0394136
H	-8.8365892	7.0469163	1.6342266
H	1.8903548	6.5496013	1.6298286
H	-1.1745662	-0.6068167	3.9562546
H	-1.9800022	0.7024043	4.8789566
H	-0.3125022	0.9310293	4.2781756
H	-0.3347632	7.9870463	-1.7635154
H	0.5525138	4.4435453	1.6030926
H	1.4417448	8.3179103	-0.0576494
H	-1.9153152	3.1814773	-3.8990644
H	-3.0817642	2.4760423	-5.0582904
H	-2.0127112	1.4051923	-4.0990404
H	-8.2729312	8.7410243	-0.0943234
H	-7.6676152	4.8430133	1.6359856
H	-1.6636952	5.8754983	-1.7742404
H	-5.3907982	6.0290773	-1.7962454
H	-6.5508552	8.2379373	-1.8137974
H	17.1597278	2.6570273	0.3251476
H	13.5112798	4.9537523	0.7411626
H	15.9495058	6.4583023	1.0706296
H	17.5193108	5.4877953	0.8943316
H	17.0490428	-4.9855377	-1.2006254
H	15.3981028	-5.8081667	-1.3886264
O	-0.8926282	-3.2068937	3.5478646
H	-0.7471572	-3.4448447	2.6135146
O	-2.1085322	-3.9518507	3.8268766
H	-2.7751272	-3.3225187	3.4759296
C	10.9896438	-3.8104707	1.3555716
O	12.1993768	-3.7527317	1.9189616
O	10.7165438	-3.4414767	0.2323446
H	10.2643708	-4.2436607	2.0576896
H	12.8276708	-3.3793567	1.2627536

[2OH]

Atom	x	y	z
N	14.0225338	-2.5376164	-0.4999797
C	14.2131808	-3.8370704	-0.7330487
Cu	15.3746728	-0.0867444	-0.0230917
N	15.2616308	-1.9351594	-0.3643577
C	16.2011448	-2.8749344	-0.5163627
C	15.6089198	-4.1524784	-0.7616877
C	16.2166328	-5.3820254	-0.9734137
O	-3.6907712	-2.0301814	2.9032483
C	4.5338138	0.2989356	0.7597873
C	-0.7849302	-7.4376814	0.7830063
O	-1.2507642	-3.0209754	-1.0096077
C	-4.6036002	-1.9999034	3.9728943
O	-1.9124442	0.8042316	-1.0472877
Ti	-3.5509222	-1.9109754	1.1362403
C	-1.5659522	-3.8370274	-0.0952497
C	0.6145148	-6.7711224	-1.0783047
O	-2.0088302	-0.8784064	0.8856473
O	-2.3807502	-3.5692174	0.8490753
Ti	-1.7476202	-1.0262734	-1.3032817
C	-0.9526482	-5.1962574	-0.1170997

C	-1.3613862	-6.1691014	0.8062543
C	0.2031458	-7.7395704	-0.1581447
C	-2.2156782	-2.1170324	-4.0645307
C	2.3771508	-0.2995334	-0.1687497
C	5.1853548	-0.4445184	-0.2441837
C	3.1462568	0.3692176	0.7944553
N	6.5785198	-0.5753714	-0.3173897
O	0.3493818	0.4780536	0.7781073
C	0.0385938	-5.5025604	-1.0601707
O	0.2409888	-0.8584804	-1.0195227
O	-1.7569362	-1.2302984	-3.0714827
C	0.8958298	-0.2198974	-0.1293677
C	3.0223608	-1.0570704	-1.1601607
C	4.4072748	-1.1392914	-1.1891867
O	-5.1165582	1.4365646	2.9019023
C	-7.2243212	-6.9127624	0.8594783
C	-11.2743322	1.3496556	0.9639333
O	-7.2109602	-0.2937134	-0.9709387
C	-4.6669342	2.3584306	3.8665613
O	-3.5706602	-1.5925344	-1.0187167
Ti	-5.1020042	1.2067776	1.1345753
C	-7.7533302	0.3973996	-0.0572687
C	-11.3954102	-0.1144244	-0.9618907
O	-4.9471962	-0.6302744	0.9024173
O	-7.1074922	1.0198526	0.8447823
Ti	-5.2365852	-0.8209614	-1.2975007
C	-9.2425692	0.4898886	-0.0332407
C	-9.8833942	1.2657806	0.9432013
C	-12.0312162	0.6607586	0.0120623
C	-5.9212302	0.0882686	-4.0782727
C	-6.6754222	-4.7551504	-0.0923207
C	-8.2054922	-7.0930614	-0.1195717
C	-6.4599662	-5.7476844	0.8743703
O	-4.9877082	-3.3715454	0.8339963
C	-10.0047902	-0.2011414	-0.9858777
O	-6.0781792	-2.6447684	-0.9865997
O	-5.4150642	-0.7506304	-3.0663097
C	-5.8568632	-3.5076054	-0.0779717
C	-7.6600572	-4.9392564	-1.0734947
C	-8.4218742	-6.1061694	-1.0853477
N	10.9550948	0.8861356	0.0623383
C	9.7071928	1.3717766	0.1272633
Cu	12.4410618	-1.5122324	-0.3456217
N	10.8704668	-0.4651054	-0.1867367
C	9.5771188	-0.7961494	-0.2711747
C	8.7758658	0.3421656	-0.0782517
C	7.3309508	0.4396786	-0.0866187
O	-1.4404932	0.9846856	2.8802453
C	-7.5920592	6.8880976	0.7723563
C	1.5936888	6.2709506	0.8375113
O	-1.8719722	3.5395236	-1.0289257
C	-0.9528142	0.4470166	4.0878763
O	-4.8278192	1.0343456	-1.0494657
Ti	-1.6176072	1.0058396	1.1239213
C	-1.0004012	3.6819006	-0.1201697
C	0.3709038	7.0762726	-1.0919777
O	-3.2952332	1.7731776	0.8580703
O	-0.7834752	2.8315376	0.8006213
Ti	-3.3185192	2.0846386	-1.3188947
C	-0.1743912	4.9250736	-0.1237117
C	0.8256708	5.1080236	0.8418833
C	1.3676118	7.2553966	-0.1285527
C	-2.1661672	2.2056676	-4.0934677
C	-5.9958822	5.3210986	-0.1546097
C	-7.2487102	7.8181366	-0.2129697
C	-6.9684862	5.6422556	0.8029423
O	-5.6407692	3.1813856	0.8032903
C	-0.3991962	5.9148356	-1.0910437
O	-4.4770692	3.7306486	-1.0342747

O	-3.1524762	2.1796526	-3.0886897
C	-5.3280092	3.9869286	-0.1235297
C	-5.6545742	6.2557726	-1.1425407
C	-6.2805102	7.5006336	-1.1696757
N	15.4982638	1.7609146	0.3185743
C	16.5514748	2.5677886	0.4896643
Cu	12.6474598	1.7164426	0.2494953
N	14.3481118	2.5224276	0.4332493
C	14.7058298	3.7853126	0.6727933
C	16.1301448	3.9127206	0.7272883
C	16.8921378	5.0507356	0.9528063
H	13.3715658	-4.5027294	-0.8716917
H	17.2504748	-2.6216864	-0.4482697
H	5.1223548	0.7895566	1.5297413
H	-1.1009422	-8.1875984	1.5027003
H	-4.0556362	-2.0908294	4.9180473
H	-5.3107592	-2.8341284	3.8815843
H	-5.1547732	-1.0533234	3.9551203
H	1.3854878	-7.0049294	-1.8074347
H	-2.1085972	-5.9095164	1.5486043
H	0.6542468	-8.7283324	-0.1733847
H	-3.3118872	-2.1201464	-4.0775237
H	-1.8515352	-3.1308544	-3.8550127
H	-1.8381212	-1.7911764	-5.0415617
H	2.6399218	0.9360106	1.5685323
H	0.3460218	-4.7386604	-1.7663547
H	2.4206728	-1.5799224	-1.8957797
H	4.9172838	-1.7289264	-1.9446967
H	-7.0552422	-7.6804944	1.6095733
H	-11.7690272	1.9512566	1.7216233
H	-4.9818972	2.0226956	4.8621673
H	-5.0976332	3.3475276	3.6648843
H	-3.5731262	2.4272256	3.8309173
H	-11.9841662	-0.6511044	-1.7008767
H	-9.2796322	1.7925076	1.6743103
H	-13.1159642	0.7272196	0.0296923
H	-6.9906072	0.2692066	-3.9106627
H	-5.7851972	-0.3991484	-5.0514327
H	-5.3842452	1.0438406	-4.0680107
H	-8.8006862	-8.0024694	-0.1301537
H	-5.6918582	-5.5926954	1.6244163
H	-9.4940212	-0.7999134	-1.7323097
H	-7.8134872	-4.1628824	-1.8152567
H	-9.1840482	-6.2471104	-1.8469017
H	9.5349178	2.4233546	0.3161933
H	9.2667188	-1.8132634	-0.4630477
H	6.9117048	1.4392526	0.1022583
H	-8.3459382	7.1346016	1.5150983
H	2.3677368	6.4111706	1.5872053
H	-0.8608812	-0.6420334	4.0030733
H	-1.6458972	0.6967776	4.9005543
H	0.0297398	0.8829716	4.3079873
H	0.1943278	7.8424166	-1.8420637
H	0.9857188	4.3336716	1.5843183
H	1.9670338	8.1621236	-0.1301317
H	-1.4727702	3.0367676	-3.9119457
H	-2.6484712	2.3433226	-5.0690467
H	-1.6105622	1.2607086	-4.0890497
H	-7.7359912	8.7894506	-0.2356267
H	-7.2240302	4.9068216	1.5581963
H	-1.1789722	5.7594696	-1.8288617
H	-4.9017342	5.9912596	-1.8772147
H	-6.0145572	8.2235476	-1.9361067
H	17.5588918	2.1772526	0.4400313
H	13.9577378	4.5568836	0.7981113
H	16.4251658	6.0144776	1.1195303
H	17.9749738	5.0082736	0.9687633
H	17.2956228	-5.4839914	-0.9695947
H	15.6286978	-6.2755474	-1.1482947

O	-0.6313192	-3.2525564	3.6362443
H	-0.4844992	-3.5106874	2.7075033
O	-1.8643472	-3.9668934	3.9201223
H	-2.5154322	-3.3295424	3.5548973

[CO_2]

Atom	x	y	z
N	13.7241862	2.9564945	0.2272202
C	13.8946452	4.2657765	0.4167562
Cu	15.1180082	0.5143285	-0.1810648
N	14.9725912	2.3690815	0.1115772
C	15.8973222	3.3278415	0.2319362
C	15.2853252	4.6035905	0.4345572
C	15.8738682	5.8485875	0.6057932
O	-3.9630088	2.1113245	-2.9955278
C	4.2766332	-0.1119235	-0.8591098
C	-0.8935918	7.4464925	-1.0334708
O	-1.5071548	3.1125095	0.9149812
C	-4.9485678	2.1310535	-4.0009298
O	-2.1732848	-0.7054375	0.9879542
Ti	-3.8072868	2.0072225	-1.2246168
C	-1.8212138	3.9193335	-0.0094758
C	0.4103052	6.8368205	0.9145772
O	-2.2969218	0.9642615	-0.9489108
O	-2.6640938	3.6600375	-0.9274048
Ti	-2.0246658	1.1278825	1.2350862
C	-1.1654718	5.2598925	-0.0302138
C	-1.5084848	6.1959475	-1.0161938
C	0.0652872	7.7682475	-0.0688378
C	-2.5154898	2.2243425	3.9880982
C	2.1137792	0.4603865	0.0739662
C	4.9196092	0.6756155	0.1163622
C	2.8905622	-0.2162165	-0.8776558
N	6.3095902	0.8432715	0.1698052
O	0.0951072	-0.3530485	-0.8615368
C	-0.2023128	5.5853475	0.9353512
O	-0.0244438	0.9611245	0.9534712
O	-2.0296208	1.3438545	3.0024622
C	0.6316592	0.3467095	0.0503832
C	2.7506392	1.2579015	1.0392092
C	4.1334362	1.3758145	1.0506922
O	-5.3510028	-1.3311705	-2.9897978
C	-7.5333988	6.9752175	-0.9772768
C	-11.5239468	-1.3050185	-1.0835478
O	-7.4808018	0.3636645	0.8723192
C	-4.8662178	-2.2106935	-3.9766928
O	-3.8428168	1.6917795	0.9557132
Ti	-5.3535788	-1.1161635	-1.2220418
C	-8.0140778	-0.3294755	-0.0441248
C	-11.6643718	0.1569915	0.8424242
O	-5.2097888	0.7171615	-0.9740728
O	-7.3604558	-0.9473315	-0.9444778
Ti	-5.5051788	0.9064775	1.2032202
C	-9.5028798	-0.4321615	-0.0759558
C	-10.1336758	-1.2117685	-1.0558978
C	-12.2901948	-0.6218195	-0.1350888
C	-6.2255368	-0.0195115	3.9693802
C	-6.9748118	4.8225535	-0.0199128
C	-8.5296508	7.1443525	-0.0115648
C	-6.7568428	5.8181145	-0.9828478
O	-5.2689668	3.4516385	-0.9320388
C	-10.2744708	0.2529885	0.8732962
O	-6.3591968	2.7236245	0.8886672
O	-5.7037438	0.8251005	2.9709272
C	-6.1428828	3.5832995	-0.0245418
C	-7.9741958	4.9956695	0.9482002
C	-8.7485908	6.1543855	0.9504752
N	10.7153522	-0.5298915	-0.2020398
C	9.4788752	-1.0466735	-0.2355308

Cu	12.1570912	1.9042805	0.1091652
N	10.6023412	0.8276495	-0.0043838
C	9.3027552	1.1325765	0.0807462
C	8.5262272	-0.0305825	-0.0603278
C	7.0844952	-0.1610925	-0.0327798
O	-1.6794008	-0.8135675	-2.9602898
C	-7.7995768	-6.8204275	-0.9008578
C	1.3418902	-6.1604475	-0.9584538
O	-2.1139578	-3.4408925	0.9435022
C	-1.1584228	0.0324295	-3.9581888
O	-5.0856848	-0.9532245	0.9611102
Ti	-1.8740088	-0.8933945	-1.1921128
C	-1.2433878	-3.5745915	0.0331982
C	0.1351782	-6.9742375	0.9775012
O	-3.5388318	-1.6797855	-0.9433268
O	-1.0253608	-2.7149045	-0.8792158
Ti	-3.5745078	-1.9945675	1.2371532
C	-0.4174248	-4.8182725	0.0239562
C	0.5744922	-4.9970585	-0.9507268
C	1.1237182	-7.1492675	0.0049632
C	-2.4349928	-2.1180485	4.0142202
C	-6.2222328	-5.2451855	0.0442422
C	-7.4579338	-7.7512075	0.0843562
C	-7.1845858	-5.5700985	-0.9222908
O	-5.8778998	-3.0984685	-0.9014278
C	-0.6346688	-5.8126335	0.9882022
O	-4.7189098	-3.6485005	0.9391092
O	-3.4199128	-2.1006865	3.0082042
C	-5.5636388	-3.9058085	0.0227522
C	-5.8824908	-6.1806415	1.0318922
C	-6.4999258	-7.4299475	1.0500132
N	15.2737942	-1.3383225	-0.4818498
C	16.3366722	-2.1317545	-0.6555068
Cu	12.4178472	-1.3395685	-0.3987098
N	14.1336132	-2.1163295	-0.5800738
C	14.5062532	-3.3765065	-0.8138618
C	15.9317982	-3.4843285	-0.8794388
C	16.7079932	-4.6133575	-1.1018528
H	13.0427672	4.9224495	0.5339272
H	16.9504992	3.0887075	0.1727232
H	4.8698232	-0.6110875	-1.6200608
H	-1.1608408	8.1704955	-1.7984618
H	-4.4661878	2.2689945	-4.9764918
H	-5.6461428	2.9592575	-3.8218668
H	-5.5003098	1.1837115	-3.9955248
H	1.1571362	7.0864915	1.6633732
H	-2.2539268	5.9279135	-1.7571158
H	0.5440942	8.7439445	-0.0839108
H	-3.6117358	2.2208275	3.9777062
H	-2.1531248	3.2412035	3.7900242
H	-2.1567868	1.8977375	4.9720012
H	2.3913172	-0.8175015	-1.6301718
H	0.0545582	4.8486445	1.6889142
H	2.1433092	1.7850425	1.7670792
H	4.6356792	1.9985595	1.7845942
H	-7.3624158	7.7456515	-1.7242858
H	-12.0108178	-1.9096965	-1.8438998
H	-5.2233018	-1.8815325	-4.9603548
H	-5.2308518	-3.2272475	-3.7809968
H	-3.7699648	-2.2095245	-3.9653908
H	-12.2603588	0.6891425	1.5789322
H	-9.5225588	-1.7341385	-1.7840258
H	-13.3743948	-0.6956775	-0.1580298
H	-7.2896128	-0.2087185	3.7781242
H	-6.1146678	0.4659605	4.9468572
H	-5.6813198	-0.9712025	3.9689222
H	-9.1345258	8.0474505	-0.0083658
H	-5.9774328	5.6702785	-1.7226078
H	-9.7711318	0.8544895	1.6225892

H	-8.1289178	4.2169785	1.6872582
H	-9.5225918	6.2866065	1.7016382
H	9.3329422	-2.1091205	-0.3795008
H	8.9709002	2.1489515	0.2375162
H	6.6872222	-1.1762815	-0.1820678
H	-8.5454598	-7.0698675	-1.6507028
H	2.1093462	-6.2975395	-1.7155078
H	-1.7049068	0.9827915	-3.9582798
H	-1.2670578	-0.4533215	-4.9357758
H	-0.0950668	0.2242375	-3.7655538
H	-0.0354338	-7.7437715	1.7255342
H	0.7283632	-4.2192555	-1.6909218
H	1.7227502	-8.0562605	-0.0027548
H	-1.7385788	-2.9480465	3.8387862
H	-2.9180698	-2.2515615	4.9900662
H	-1.8816798	-1.1716455	4.0056262
H	-7.9385668	-8.7260015	0.0999442
H	-7.4386558	-4.8339155	-1.6773238
H	-1.4085108	-5.6604005	1.7328952
H	-5.1375488	-5.9130425	1.7734792
H	-6.2352598	-8.1534155	1.8164132
H	17.3389622	-1.7269125	-0.6178378
H	13.7655222	-4.1567215	-0.9272098
H	16.2532982	-5.5843345	-1.2605748
H	17.7900042	-4.5560945	-1.1258688
H	16.9511612	5.9670425	0.5986762
H	15.2721472	6.7381455	0.7516482
C	11.5972912	-4.1205045	1.7768462
O	11.2944042	-4.0830645	0.6445022
O	11.8969262	-4.1593425	2.9037742

[CO_2]-b

Atom	x	y	z
N	13.7607301	2.5537201	0.0676577
C	13.9645431	3.8633921	0.2269687
Cu	15.0944011	0.0700841	-0.3043393
N	14.9933831	1.9315941	-0.0308253
C	15.9420331	2.8697501	0.0687397
C	15.3630101	4.1655291	0.2393567
C	15.9839521	5.3986991	0.3837727
O	-3.9741079	2.0275391	-3.0536773
C	4.2426961	-0.3170049	-0.9154113
C	-0.7809719	7.3549271	-1.2751533
O	-1.4585839	3.0911421	0.8025807
C	-4.9704719	2.0385551	-4.0485453
O	-2.1998199	-0.7083909	0.9928267
Ti	-3.8018439	1.9710521	-1.2820773
C	-1.7664089	3.8773431	-0.1415803
C	0.5301611	6.7756581	0.6773227
O	-2.3101109	0.9061611	-0.9908553
O	-2.6234259	3.6085531	-1.0435543
Ti	-2.0129139	1.1279391	1.1853797
C	-1.0851269	5.2036221	-0.2069753
C	-1.4197599	6.1177021	-1.2161823
C	0.1934891	7.6852091	-0.3291923
C	-2.4550089	2.3119061	3.9103997
C	2.1006671	0.3356701	0.0127597
C	4.9108541	0.4823511	0.0330417
C	2.8543741	-0.3874169	-0.9230693
N	6.3050861	0.6163001	0.0747347
O	0.0567431	-0.4564149	-0.8854943
C	-0.1063159	5.5375501	0.7397827
O	-0.0188049	0.9124521	0.8910667
O	-1.9964309	1.3942571	2.9457737
C	0.6159651	0.2567701	0.0020087
C	2.7629781	1.1452041	0.9503977
C	4.1483411	1.2291651	0.9506007
O	-5.4278419	-1.3852519	-2.9345893
C	-7.4235709	7.0198351	-1.1408653

C	-11.5798959	-1.1837349	-0.9699753
O	-7.4854389	0.4623141	0.8961947
C	-4.9699239	-2.3015319	-3.9006743
O	-3.8217959	1.7206261	0.9062707
Ti	-5.4089889	-1.1201529	-1.1737683
C	-8.0414309	-0.2466909	0.0057687
C	-11.6724649	0.3403171	0.9102707
O	-5.2263639	0.7162051	-0.9803873
O	-7.4090949	-0.9036839	-0.8820233
Ti	-5.4965339	0.9750381	1.1924627
C	-9.5321829	-0.3204879	-0.0089903
C	-10.1878569	-1.1174969	-0.9581233
C	-12.3231219	-0.4559939	-0.0364443
C	-6.2054669	0.1412091	3.9902647
C	-6.8992789	4.8839021	-0.1282433
C	-8.4046889	7.2376871	-0.1694373
C	-6.6716409	5.8467211	-1.1217513
O	-5.2323219	3.4519571	-1.0183893
C	-10.2807029	0.4093311	0.9253387
O	-6.3177499	2.7989261	0.8333277
O	-5.6789879	0.9483501	2.9635887
C	-6.0934779	3.6277041	-0.1061783
C	-7.8834529	5.1058501	0.8454777
C	-8.6331919	6.2804531	0.8230237
N	10.6746991	-0.8751309	-0.2781593
C	9.4253661	-1.3603269	-0.2954123
Cu	12.1675531	1.5433511	-0.0424523
N	10.5951581	0.4894651	-0.1115703
C	9.3032821	0.8283341	-0.0283443
C	8.4981391	-0.3177799	-0.1399493
C	7.0533941	-0.4122619	-0.1034633
O	-1.7467949	-0.9404149	-2.9541363
C	-7.9583899	-6.7656379	-0.6676943
C	1.1814181	-6.2914519	-0.8324003
O	-2.1937269	-3.4450959	1.0286617
C	-1.2213719	-0.1312569	-3.9799383
O	-5.1158719	-0.8991119	1.0017657
Ti	-1.9259769	-0.9660479	-1.1826493
C	-1.3363489	-3.6231789	0.1134677
C	-0.0175379	-7.0223669	1.1410677
O	-3.6031659	-1.7122949	-0.8952883
O	-1.1106219	-2.7945999	-0.8255163
Ti	-3.6232219	-1.9622939	1.2931447
C	-0.5367279	-4.8838499	0.1311217
C	0.4388371	-5.1121779	-0.8497043
C	0.9547251	-7.2468179	0.1622977
C	-2.4564799	-2.0290839	4.0605007
C	-6.3436349	-5.1939409	0.2182827
C	-7.6249789	-7.6742339	0.3407767
C	-7.3206529	-5.5281519	-0.7301993
O	-5.9687919	-3.0816439	-0.7911133
C	-0.7626469	-5.8447779	1.1267607
O	-4.8024679	-3.6011039	1.0536827
O	-3.4521119	-2.0204979	3.0649307
C	-5.6606189	-3.8683139	0.1527377
C	-6.0121529	-6.1071199	1.2293097
C	-6.6523809	-7.3436159	1.2885267
N	15.2081311	-1.7867679	-0.6017013
C	16.2529431	-2.5995649	-0.7914703
Cu	12.3621641	-1.7224949	-0.4569143
N	14.0527481	-2.5456199	-0.6682243
C	14.3981811	-3.8133229	-0.8971353
C	15.8200021	-3.9468859	-0.9932593
C	16.5709881	-5.0914349	-1.2206633
H	13.1293601	4.5435421	0.3292807
H	16.9888271	2.6021421	0.0183407
H	4.8185271	-0.8528169	-1.6646873
H	-1.0417569	8.0619101	-2.0580833
H	-4.4969239	2.1444181	-5.0324073

H	-5.6526459	2.8821661	-3.8825973
H	-5.5372069	1.1007371	-4.0134583
H	1.2890681	7.0319061	1.4116097
H	-2.1775939	5.8433161	-1.9420543
H	0.6909101	8.6504931	-0.3767783
H	-3.5511199	2.3321191	3.9085087
H	-2.0722929	3.3147461	3.6815247
H	-2.0953619	2.0046551	4.9001677
H	2.3356661	-0.9980529	-1.6545853
H	0.1437011	4.8177831	1.5117917
H	2.1734191	1.7081811	1.6659237
H	4.6704711	1.8604061	1.6630327
H	-7.2451449	7.7648381	-1.9115513
H	-12.0860759	-1.8020199	-1.7063983
H	-5.3284939	-1.9920219	-4.8901543
H	-5.3539969	-3.3044129	-3.6740653
H	-3.8738279	-2.3231699	-3.8981983
H	-12.2505599	0.9071211	1.6350917
H	-9.5943559	-1.6744349	-1.6751113
H	-13.4087419	-0.5087859	-0.0470553
H	-7.2741189	-0.0358169	3.8134547
H	-6.0780199	0.6525061	4.9524267
H	-5.6767469	-0.8189149	4.0123937
H	-8.9903209	8.1532461	-0.1854883
H	-5.9041489	5.6612801	-1.8654943
H	-9.7582749	1.0238271	1.6506357
H	-8.0458419	4.3520591	1.6083217
H	-9.3953829	6.4505741	1.5786227
H	9.2486991	-2.4210809	-0.4170893
H	8.9974901	1.8558221	0.1075347
H	6.6306801	-1.4209929	-0.2233983
H	-8.7155779	-7.0223169	-1.4036363
H	1.9361531	-6.4670179	-1.5942863
H	-1.7525409	0.8274861	-4.0020463
H	-1.3465989	-0.6424079	-4.9424723
H	-0.1533859	0.0485241	-3.8018813
H	-0.1947579	-7.7658809	1.9134767
H	0.5996011	-4.3598059	-1.6143193
H	1.5344531	-8.1662209	0.1741397
H	-1.7754939	-2.8744469	3.8984287
H	-2.9307989	-2.1303449	5.0444847
H	-1.8881679	-1.0923469	4.0221977
H	-8.1233589	-8.6390249	0.3884037
H	-7.5679779	-4.8091329	-1.5037873
H	-1.5236909	-5.6544039	1.8758597
H	-5.2555879	-5.8325369	1.9564347
H	-6.3940889	-8.0498149	2.0729937
H	17.2627091	-2.2121879	-0.7784483
H	13.6441811	-4.5843079	-0.9848923
H	16.0962211	-6.0564909	-1.3547703
H	17.6530471	-5.0535849	-1.2694183
H	17.0639841	5.4885081	0.3764557
H	15.4054381	6.3066411	0.5084177
C	12.7169491	0.3183541	3.1154077
O	13.2786811	-0.6104299	2.6760127
O	12.1558491	1.2434661	3.5571817

[2H_2O-CO_2]

Atom	x	y	z
N	13.5107541	-2.3340576	-0.7241300
C	13.7136311	-3.6298746	-0.9667440
Cu	14.8396811	0.1298434	-0.2491130
N	14.7441641	-1.7178026	-0.5995420
C	15.6927231	-2.6456866	-0.7678880
C	15.1125971	-3.9286566	-1.0136430
C	15.7320551	-5.1498426	-1.2393640
O	-4.1661049	-2.0533656	2.8892000
C	4.0050371	0.3840554	0.6628880
C	-1.2225339	-7.4143926	0.7043100

O	-1.7608269	-2.9935556	-1.0576300
C	-5.0664379	-2.0394526	3.9696850
O	-2.4675149	0.8237904	-1.0656260
Ti	-4.0483019	-1.9224966	1.1213820
C	-2.0558579	-3.8184646	-0.1440360
C	0.1470381	-6.7211276	-1.1694340
O	-2.5214949	-0.8705796	0.8586750
O	-2.8624779	-3.5651716	0.8110550
Ti	-2.2844369	-1.0030986	-1.3339370
C	-1.4268239	-5.1700456	-0.1809950
C	-1.8134129	-6.1528076	0.7415330
C	-0.2421479	-7.6994356	-0.2500470
C	-2.7721959	-2.0839976	-4.0957260
C	1.8447071	-0.2345046	-0.2437000
C	4.6533311	-0.3461156	-0.3528680
C	2.6172631	0.4378834	0.7141970
N	6.0469651	-0.4602196	-0.4431260
O	-0.1806909	0.5140194	0.7312320
C	-0.4433799	-5.4595296	-1.1373150
O	-0.2946169	-0.8137886	-1.0726180
O	-2.3121749	-1.1974886	-3.1030810
C	0.3630851	-0.1724396	-0.1864960
C	2.4869821	-0.9788646	-1.2469280
C	3.8723221	-1.0446946	-1.2926350
O	-5.6320369	1.3965484	2.9243500
C	-7.6662459	-6.9654256	0.8592060
C	-11.8107799	1.2482404	1.0581820
O	-7.7516029	-0.3365946	-0.9334910
C	-5.1818549	2.3182424	3.8889140
O	-4.0972189	-1.5923816	-1.0312950
Ti	-5.6356229	1.1767814	1.1556930
C	-8.2911919	0.3430194	-0.0096050
C	-11.9374619	-0.2064246	-0.8744050
O	-5.4621839	-0.6570346	0.9112940
O	-7.6420719	0.9679734	0.8883510
Ti	-5.7751989	-0.8388356	-1.2861570
C	-9.7810209	0.4179094	0.0323920
C	-10.4192999	1.1807934	1.0206800
C	-12.5707429	0.5558234	0.1113310
C	-6.5031059	0.0777564	-4.0534910
C	-7.1537849	-4.7962656	-0.0866470
C	-8.6567139	-7.1517656	-0.1092970
C	-6.9154009	-5.7915676	0.8717800
O	-5.4715119	-3.3981506	0.8277090
C	-10.5463029	-0.2766996	-0.9151740
O	-6.5917569	-2.6740866	-0.9758180
O	-5.9753119	-0.7607676	-3.0523260
C	-6.3497109	-3.5393106	-0.0747780
C	-8.1476949	-4.9864416	-1.0572530
C	-8.8959459	-6.1621206	-1.0668310
N	10.4103771	1.0503524	-0.1063530
C	9.1577511	1.5209714	-0.0240830
Cu	11.9193081	-1.3280936	-0.5453660
N	10.3385701	-0.3003896	-0.3621250
C	9.0482711	-0.6460816	-0.4333200
C	8.2361321	0.4816684	-0.2245190
C	6.7901811	0.5622824	-0.2154130
O	-1.9514629	0.9879214	2.8570580
C	-8.1958269	6.8304484	0.8550520
C	0.9966211	6.3206704	0.8090810
O	-2.4587129	3.5592264	-1.0321680
C	-1.4433579	0.4492954	4.0558090
O	-5.3851939	1.0197254	-1.0323580
Ti	-2.1494879	1.0167644	1.1030770
C	-1.5782159	3.7067544	-0.1328860
C	-0.2581119	7.1223224	-1.1013290
O	-3.8389599	1.7658494	0.8612750
O	-1.3405609	2.8538844	0.7804390
Ti	-3.8915169	2.0891204	-1.3134590

C	-0.7668519	4.9595264	-0.1390190
C	0.2423161	5.1488124	0.8158220
C	0.7477201	7.3077544	-0.1486420
C	-2.7734189	2.2390794	-4.1006050
C	-6.5925229	5.2874474	-0.0994640
C	-7.8749729	7.7699134	-0.1289120
C	-7.5574579	5.5918514	0.8712310
O	-6.2012339	3.1467494	0.8420000
C	-1.0145569	5.9519484	-1.0979930
O	-5.0657429	3.7198284	-1.0059100
O	-3.7474529	2.1959164	-3.0845060
C	-5.9088239	3.9610454	-0.0838130
C	-6.2737939	6.2315374	-1.0859820
C	-6.9144749	7.4691074	-1.0986880
N	14.9457441	1.9768944	0.1016140
C	15.9914211	2.7950964	0.2649400
Cu	12.0950361	1.8993954	0.0656650
N	13.7882251	2.7242244	0.2340840
C	14.1339941	3.9898634	0.4766160
C	15.5572701	4.1336544	0.5151470
C	16.3085501	5.2792514	0.7382070
H	12.8778781	-4.3000886	-1.1205490
H	16.7400551	-2.3788646	-0.7202770
H	4.5974791	0.8786634	1.4274270
H	-1.5256579	-8.1755546	1.4182120
H	-4.5054769	-2.1228036	4.9086600
H	-5.7583709	-2.8877676	3.8875570
H	-5.6373919	-1.1034106	3.9631360
H	0.9116811	-6.9423636	-1.9091540
H	-2.5760549	-5.9129226	1.4749040
H	0.2188461	-8.6834486	-0.2771880
H	-3.8684329	-2.0981436	-4.0976990
H	-2.3959379	-3.0951206	-3.8945280
H	-2.4078329	-1.7499896	-5.0751410
H	2.1134441	0.9963974	1.4959900
H	-0.1517469	-4.6861666	-1.8396530
H	1.8821541	-1.5064146	-1.9764770
H	4.3804711	-1.6242966	-2.0571200
H	-7.4798669	-7.7354266	1.6029840
H	-12.3035589	1.8398104	1.8250170
H	-5.4706709	1.9672614	4.8873190
H	-5.6352209	3.3008474	3.7053810
H	-4.0904059	2.4088714	3.8334270
H	-12.5286249	-0.7458616	-1.6094940
H	-9.8128489	1.7102904	1.7475520
H	-13.6559419	0.6094924	0.1420200
H	-7.5721169	0.2471734	-3.8715470
H	-6.3747509	-0.4032666	-5.0309700
H	-5.9758579	1.0388194	-4.0455670
H	-9.2414339	-8.0680216	-0.1181060
H	-6.1409189	-5.6296356	1.6137850
H	-10.0370949	-0.8652256	-1.6707650
H	-8.3184999	-4.2075826	-1.7925870
H	-9.6654299	-6.3078916	-1.8201450
H	8.9754091	2.5699004	0.1705690
H	8.7486091	-1.6650246	-0.6321310
H	6.3607471	1.5559944	-0.0175160
H	-8.9438469	7.0639524	1.6078920
H	1.7781111	6.4656804	1.5501370
H	-1.8015199	-0.5787146	4.1799790
H	-1.7837049	1.0596584	4.9012970
H	-0.3461719	0.4545654	4.0268500
H	-0.4522709	7.8904304	-1.8450560
H	0.4203631	4.3718324	1.5513820
H	1.3366531	8.2213474	-0.1522360
H	-2.0896389	3.0793534	-3.9245960
H	-3.2689359	2.3728834	-5.0701440
H	-2.2045309	1.3020454	-4.1057280
H	-8.3738479	8.7355534	-0.1403850

H	-7.7953569	4.8490484	1.6250380
H	-1.8010059	5.7912374	-1.8275310
H	-5.5266829	5.9796824	-1.8308870
H	-6.6660949	8.1993484	-1.8641050
H	17.0027171	2.4166104	0.2019570
H	13.3782641	4.7516224	0.6152020
H	15.8325031	6.2362974	0.9180110
H	17.3920671	5.2498074	0.7409010
H	16.8121871	-5.2383596	-1.2551020
H	15.1527581	-6.0487296	-1.4157440
O	12.7668671	-0.3761926	1.7121200
H	11.9302221	-0.2673856	2.1909640
H	13.1674231	-1.2129636	2.0561330
O	13.8355371	-2.8313736	2.3857690
H	13.7426951	-3.2146066	1.4948740
H	14.7948241	-2.7670556	2.5149060
C	-0.3653919	-3.0794276	2.8906860
O	-0.9865879	-3.1732256	3.8777620
O	0.2818571	-2.9899976	1.9212770

[2OH-HCOOH]

Atom	x	y	z
N	13.6247557	-2.3573784	-0.7998227
C	13.8238657	-3.6523304	-1.0501947
Cu	14.9612777	0.1003296	-0.3140897
N	14.8601557	-1.7451154	-0.6746267
C	15.8058937	-2.6744684	-0.8502867
C	15.2218457	-3.9545724	-1.1017857
C	15.8376067	-5.1760454	-1.3358217
O	-4.0430243	-2.0506604	2.8547193
C	4.1293997	0.3776846	0.6236543
C	-1.1184633	-7.4072144	0.6333103
O	-1.6491823	-2.9752104	-1.1026127
C	-4.9410273	-2.0407224	3.9372193
O	-2.3458183	0.8439416	-1.0878497
Ti	-3.9290023	-1.9104324	1.0874003
C	-1.9441633	-3.8042924	-0.1932477
C	0.2487197	-6.7070724	-1.2396127
O	-2.3999493	-0.8610104	0.8270913
O	-2.7480473	-3.5545454	0.7652753
Ti	-2.1680813	-0.9819954	-1.3667867
C	-1.3189843	-5.1575034	-0.2389817
C	-1.7059613	-6.1443194	0.6789053
C	-0.1409733	-7.6894814	-0.3248317
C	-2.6648803	-2.0460734	-4.1334197
C	1.9654397	-0.2301434	-0.2815347
C	4.7735067	-0.3484864	-0.3976147
C	2.7418867	0.4348326	0.6783903
N	6.1666347	-0.4657174	-0.4916457
O	-0.0558113	0.5181606	0.7021373
C	-0.3383163	-5.4441324	-1.1991377
O	-0.1772903	-0.7991344	-1.1088077
O	-2.2003033	-1.1663204	-3.1368807
C	0.4841567	-0.1645564	-0.2206747
C	2.6035197	-0.9705954	-1.2903147
C	3.9885777	-1.0397674	-1.3395077
O	-5.4999163	1.4026486	2.9123753
C	-7.5606263	-6.9423284	0.8051783
C	-11.6831783	1.2808746	1.0592963
O	-7.6326223	-0.3033874	-0.9503037
C	-5.0451723	2.3177626	3.8810553
O	-3.9817743	-1.5681174	-1.0633377
Ti	-5.5080273	1.1927736	1.1425333
C	-8.1683653	0.3724606	-0.0214297
C	-11.8179833	-0.1626384	-0.8810877
O	-5.3399123	-0.6400684	0.8874923
O	-7.5156083	0.9906936	0.8785393
Ti	-5.6583343	-0.8088024	-1.3102067
C	-9.6578963	0.4509976	0.0243313

C	-10.2919673	1.2100096	1.0182933
C	-12.4470643	0.5957396	0.1103073
C	-6.3900113	0.1251296	-4.0707417
C	-7.0445973	-4.7692624	-0.1297027
C	-8.5537383	-7.1206734	-0.1621207
C	-6.8066843	-5.7705194	0.8226173
O	-5.3566393	-3.3806614	0.7886553
C	-10.4271083	-0.2363094	-0.9253747
O	-6.4790003	-2.6436214	-1.0082797
O	-5.8621813	-0.7203474	-3.0754567
C	-6.2372103	-3.5144924	-0.1126267
C	-8.0411693	-4.9514224	-1.0991197
C	-8.7925163	-6.1250754	-1.1135777
N	10.5347287	1.0315756	-0.1562697
C	9.2835267	1.5049816	-0.0685237
Cu	12.0364277	-1.3483324	-0.6119217
N	10.4588207	-0.3175164	-0.4194027
C	9.1674577	-0.6594574	-0.4895947
C	8.3587427	0.4692246	-0.2727037
C	6.9130307	0.5535546	-0.2598907
O	-1.8205873	0.9848096	2.8345353
C	-8.0540793	6.8546856	0.8791973
C	1.1368757	6.3212116	0.8097303
O	-2.3297803	3.5791136	-1.0391307
C	-1.3112193	0.4381746	4.0291183
O	-5.2628953	1.0472896	-1.0469177
Ti	-2.0224503	1.0239666	1.0811933
C	-1.4468933	3.7193206	-0.1410207
C	-0.1200143	7.1367916	-1.0933527
O	-3.7104893	1.7787926	0.8473703
O	-1.2094363	2.8607466	0.7669943
Ti	-3.7670543	2.1143426	-1.3254007
C	-0.6322673	4.9699876	-0.1419867
C	0.3795207	5.1513056	0.8116273
C	0.8884237	7.3142776	-0.1419077
C	-2.6547913	2.2769526	-4.1141717
C	-6.4569573	5.3128676	-0.0875137
C	-7.7329633	7.7987946	-0.1002267
C	-7.4189223	5.6143586	0.8870303
O	-6.0691753	3.1659306	0.8411103
C	-0.8795133	5.9683946	-1.0948497
O	-4.9363143	3.7463606	-1.0061197
O	-3.6266633	2.2306536	-3.0961447
C	-5.7767013	3.9846216	-0.0808007
C	-6.1379603	6.2616196	-1.0694617
C	-6.7754233	7.5009066	-1.0738227
N	15.0729597	1.9451096	0.0466903
C	16.1211387	2.7596576	0.2122403
Cu	12.2219867	1.8752406	0.0167313
N	13.9177007	2.6947016	0.1859373
C	14.2673237	3.9580616	0.4347493
C	15.6910557	4.0979236	0.4708853
C	16.4458287	5.2402956	0.6986443
H	12.9861467	-4.3239354	-1.1842197
H	16.8533677	-2.4118224	-0.7910777
H	4.7243857	0.8654706	1.3904373
H	-1.4175683	-8.1679904	1.3488293
H	-4.3786783	-2.1318794	4.8738623
H	-5.6385113	-2.8834084	3.8491093
H	-5.5046213	-1.1013164	3.9348023
H	1.0115427	-6.9255754	-1.9819387
H	-2.4449203	-5.8998954	1.4345543
H	0.3187967	-8.6738244	-0.3575087
H	-3.7611053	-2.0582344	-4.1320857
H	-2.2897053	-3.0590284	-3.9397537
H	-2.3029313	-1.7065744	-5.1117127
H	2.2413087	0.9884746	1.4656473
H	-0.0476283	-4.6695614	-1.9007717
H	1.9963227	-1.4905564	-2.0234757

H	4.4934587	-1.6159474	-2.1087167
H	-7.3741353	-7.7168174	1.5441213
H	-12.1726303	1.8694106	1.8305353
H	-5.3416063	1.9671526	4.8772313
H	-5.4889223	3.3046476	3.6976253
H	-3.9527843	2.3982896	3.8298363
H	-12.4121593	-0.6963984	-1.6178497
H	-9.6827833	1.7340466	1.7468343
H	-13.5320173	0.6520416	0.1437543
H	-7.4583123	0.2951846	-3.8857447
H	-6.2640693	-0.3505384	-5.0510507
H	-5.8608703	1.0850416	-4.0577607
H	-9.1407883	-8.0353374	-0.1747167
H	-6.0295843	-5.6168694	1.5636223
H	-9.9214273	-0.8219334	-1.6855747
H	-8.2119413	-4.1682394	-1.8298547
H	-9.5639663	-6.2644914	-1.8660207
H	9.1049717	2.5530226	0.1336193
H	8.8635557	-1.6770514	-0.6893257
H	6.4870697	1.5471856	-0.0548247
H	-8.7996813	7.0859446	1.6350853
H	1.9201897	6.4600356	1.5499963
H	-1.2125593	-0.6492254	3.9314143
H	-1.9941043	0.6741036	4.8544763
H	-0.3287373	0.8791026	4.2394143
H	-0.3139053	7.9095736	-1.8322767
H	0.5570817	4.3700966	1.5428073
H	1.4796557	8.2263696	-0.1416467
H	-1.9688643	3.1147446	-3.9350597
H	-3.1521973	2.4170616	-5.0817897
H	-2.0879823	1.3387326	-4.1255647
H	-8.2292593	8.7657896	-0.1051287
H	-7.6571993	4.8682136	1.6374083
H	-1.6680393	5.8141026	-1.8235377
H	-5.3930893	6.0122526	-1.8174377
H	-6.5267473	8.2347606	-1.8356457
H	17.1313817	2.3788406	0.1461063
H	13.5138407	4.7206446	0.5806423
H	15.9725617	6.1974806	0.8847593
H	17.5292307	5.2077926	0.6995733
H	16.9173337	-5.2680404	-1.3466817
H	15.2553897	-6.0729934	-1.5116677
O	-0.9671113	-3.2592584	3.5340073
H	-0.8296123	-3.5040904	2.6002363
O	-2.1907803	-3.9873604	3.8233733
H	-2.8513003	-3.3507344	3.4741573
C	13.2572767	-1.1734644	2.7481763
O	13.5791137	-2.4135694	2.3793973
O	13.0543887	-0.2418244	1.9965273
H	13.1980737	-1.1039614	3.8429963
H	13.6267297	-2.4421764	1.3974743

Supplementary References

1. Hong, K. & Chun, H. Nonporous titanium-oxo molecular clusters that reversibly and selectively adsorb carbon dioxide. *Inorg. Chem.* **52**, 9705-9707 (2013).
2. Li, X. M. *et al.* An imine-linked metal-organic framework as a reactive oxygen species generator. *Angew. Chem. Int. Ed.* **60**, 2534-2540 (2020).
3. Nguyen, H. L. *et al.* A titanium-organic framework as an exemplar of combining the chemistry of metal- and covalent-organic frameworks. *J. Am. Chem. Soc.* **138**, 4330-4333 (2016).
4. Zhou, H. G. *et al.* Acid-triggered interlayer sliding of two-dimensional copper(I)-organic frameworks: more metal sites for catalysis. *Chem. Sci.* **12**, 6280-6286 (2021).
5. Li, L. *et al.* Isoreticular Series of Two-Dimensional Covalent Organic Frameworks with the kgd Topology and Controllable Micropores. *J. Am. Chem. Soc.* **144**, 6475-6482 (2022).
6. Yan, Z. H. *et al.* Photo-generated dinuclear {Eu(II)}$_2$ active sites for selective CO_2 reduction in a photosensitizing metal-organic framework. *Nat. Commun.* **9**, 3353 (2018).
7. Gong, Y. N. *et al.* Regulating photocatalysis by spin-state manipulation of cobalt in covalent organic frameworks. *J. Am. Chem. Soc.* **142**, 16723-16731 (2020).
8. Zhuo, T. C. *et al.* H-bond-mediated selectivity control of formate versus CO during CO_2 photoreduction with two cooperative Cu/X sites. *J. Am. Chem. Soc.* **143**, 6114-6122 (2021).
9. Xu, H. Q. *et al.* Visible-light photoreduction of CO_2 in a metal-organic framework: boosting electron-hole separation via electron trap states. *J. Am. Chem. Soc.* **137**, 13440-13443 (2015).
10. Chen, D. S. *et al.* Highly efficient visible-light-driven CO_2 reduction to formate by a new anthracene-based zirconium MOF via dual catalytic routes. *J. Mater. Chem. A* **4**, 2657-2662 (2016).
11. Zhu, Q.*, et al.* CO_2 reduction to formic acid *via* NH_2-C@Cu_2O photocatalyst *in situ* derived from amino modified Cu-MOF. *J. CO_2 Util.* **54**, 101781 (2021).
12. Li, N. *et al.* Adenine components in biomimetic metal-organic frameworks for efficient CO_2 photoconversion. *Angew. Chem. Int. Ed.* **58**, 5226-5231 (2019).
13. Yang, T. C. *et al.* Photocatalytic reduction of CO_2 with SiC recovered from silicon sludge wastes. *Environ. Technol.* **36**, 2987-2990 (2015).
14. Mele, G. *et al.* Photoreduction of carbon dioxide to formic acid in aqueous suspension: a comparison between phthalocyanine/TiO_2 and porphyrin/TiO_2 catalysed processes. *Molecules* **20**, 396-415 (2015).
15. Dong, L. Z. *et al.* Stable heterometallic cluster-based organic framework catalysts for artificial photosynthesis. *Angew. Chem. Int. Ed.* **59**, 2659-2663 (2020).
16. Wang, Y. *et al.* Direct and indirect Z-scheme heterostructure-coupled photosystem enabling cooperation of CO_2 reduction and H_2O oxidation. *Nat. Commun.* **11**, 3043 (2020).
17. Zhou, J. *et al.* Synergetic effect of H^+ adsorption and ethylene functional groups

of covalent organic frameworks on the CO_2 photoreduction in aqueous solution. *Chem. Commun.* **56**, 7261-7264 (2020).

18. Wang, J. L. et al. Linking the dynamic chemical state of catalysts with the product profile of electrocatalytic CO_2 reduction. *Angew. Chem. Int. Ed.* **60**, 17254-17267 (2021).
19. Kobayashi, K. et al. Selective generation of formamides through photocatalytic CO_2 reduction catalyzed by ruthenium carbonyl compounds. *Angew. Chem. Int. Ed.* **53**, 11813-11817 (2014).
